# Multiparameter persistent homology landscapes identify immune cell spatial patterns in tumors

**DOI:** 10.1073/pnas.2102166118

**Published:** 2021-10-08

**Authors:** Oliver Vipond, Joshua A. Bull, Philip S. Macklin, Ulrike Tillmann, Christopher W. Pugh, Helen M. Byrne, Heather A. Harrington

**Affiliations:** ^a^Mathematical Institute, University of Oxford, Oxford OX2 6GG, United Kingdom;; ^b^Nuffield Department of Medicine Research Building, University of Oxford, Oxford OX3 7FZ, United Kingdom;; ^c^Wellcome Centre for Human Genetics, University of Oxford, Oxford OX3 7BN, United Kingdom

**Keywords:** topological data analysis, digital pathology, histology data, tumor immunology, hypoxia

## Abstract

Highly resolved spatial data of complex systems encode rich and nonlinear information. Quantification of heterogeneous and noisy data—often with outliers, artifacts, and mislabeled points—such as those from tissues, remains a challenge. The mathematical field that extracts information from the shape of data, topological data analysis (TDA), has expanded its capability for analyzing real-world datasets in recent years by extending theory, statistics, and computation. An extension to the standard theory to handle heterogeneous data is multiparameter persistent homology (MPH). Here we provide an application of MPH landscapes, a statistical tool with theoretical underpinnings. MPH landscapes, computed for (noisy) data from agent-based model simulations of immune cells infiltrating into a spheroid, are shown to surpass existing spatial statistics and one-parameter persistent homology. We then apply MPH landscapes to study immune cell location in digital histology images from head and neck cancer. We quantify intratumoral immune cells and find that infiltrating regulatory T cells have more prominent voids in their spatial patterns than macrophages. Finally, we consider how TDA can integrate and interrogate data of different types and scales, e.g., immune cell locations and regions with differing levels of oxygenation. This work highlights the power of MPH landscapes for quantifying, characterizing, and comparing features within the tumor microenvironment in synthetic and real datasets.

## Significance

Quantifying and comparing complex spatial biological datasets is crucial for medical applications and remains an active area of research. As datasets become more heterogeneous and complicated, so must the methods that are used to understand them. Multiparameter topology is built upon the assumption that the shape of data depends on multiple parameters, such as scale, outliers, or other parameters (e.g., cell density and oxygen levels in the case of tumors). A key difficulty encountered in multiparameter persistent homology (MPH) is interpreting and comparing data. The present work uses statistical MPH landscapes to overcome this difficulty and quantifies differences in synthetic data of immune cell infiltration as well as clinical tumor histology data of T cells, macrophages, and hypoxia.

Advances in topological data analysis (TDA), an emerging field of mathematics that studies shape within datasets, offer novel descriptors of spatial data that have the potential to inform histological analysis. Its primary technique, single-parameter persistent homology (1-PH), provides a multiscale topological summary of data and benefits from a rigorous theoretical underpinning (1–3). While 1-PH has proven successful for many types of datasets and applications (4–10), including histology datasets (11–13), and is tolerant to small imperfections in the registration of points, its utility can be diminished when analyzing data with large amounts of noise (e.g., outliers, including points that are misclassified), typical of biological datasets. The case for considering multiscale data with outliers was, in part, a driver of the creation of multiparameter persistent homology (MPH) (14–16), a theoretically and computationally challenging area of active mathematical research (17, 18). Moreover, the need to compare topological summaries and their vectorizations has driven the development of statistical methodologies for 1-PH (19, 20) and, more recently, MPH landscapes (21).

Interest in the distribution of immune cells in tumors relative to areas of hypoxia (reduced oxygen availability) is high because of the associations between immunogenicity, tumor hypoxia, and prognosis (22, 23). The pattern of immune cell infiltration may also be relevant to the focused application of cancer immunotherapy (24) or new opportunities to manipulate oxygen-sensing pathways (25). However, current histopathological practice does not include rigorous analysis of either the extent of tumor hypoxia [which can be assessed in a variety of ways (26)] or the number and distribution of different types of infiltrating immune cells. While international consensus guidelines exist to facilitate visual enumeration of tumor infiltrating leukocytes (27, 28), these have not yet been adopted into routine practice. Furthermore, manual assessment is labor intensive, prone to intraobserver and interobserver variation and cannot fully characterize the complex spatial patterning of these cells. Digital pathology generates high-resolution, multiscale images allowing application of automated methods to describe and quantify the distributions of different (immune) cell types in ways that surpass the limits of human assessment (29, 30). Early work in this area has compared immune cell densities at the tumor outer margin and inner core (31, 32) or applied established spatial statistics, such as those originally developed for ecological data analysis, to histology images (33). While clearly an improvement over subjective qualitative assessment or cell counting, such approaches still fall short of a full description (34).

Motivated by the limitations of current techniques for analyzing cellular spatial patterns in histology data, here we analyze two datasets, one synthetic and one clinical, and compare results obtained from 1-PH, spatial statistics, and MPH (see *SI Appendix* for a video tutorial and ref. 35 for interactive examples). We show that these techniques provide complementary information but that, in contrast to conventional spatial statistics or 1-PH, MPH can both reveal and quantify spatial features in the face of the noise typically found in biological datasets.

## Description of Datasets

### Agent-Based Model.

**Fig. 1.**
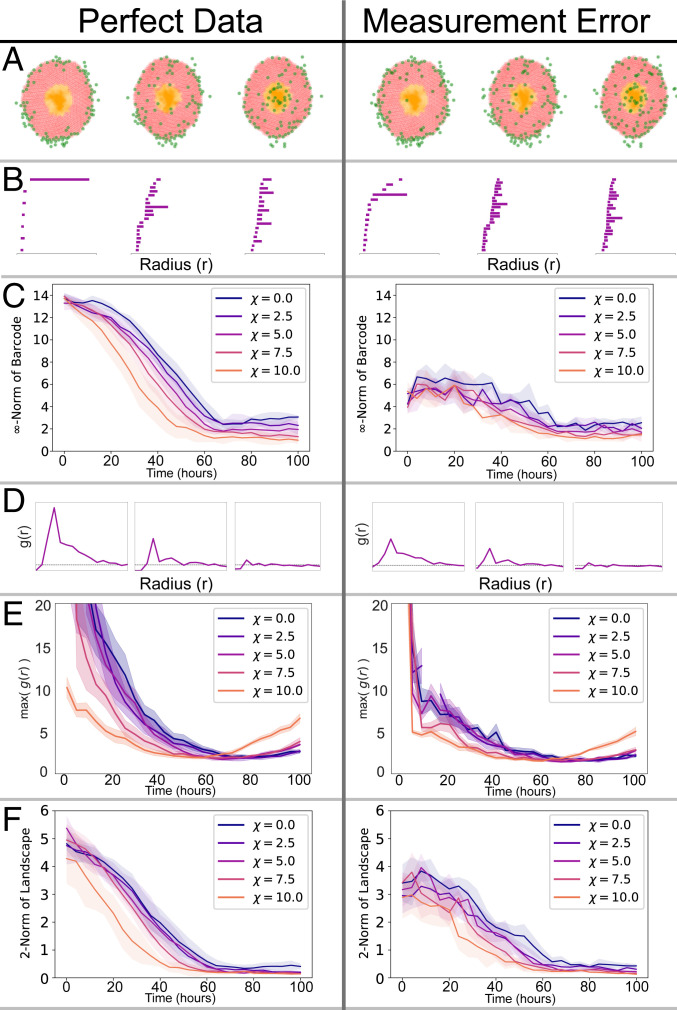
We apply 1-PH, the PCF and MPH to ABM data (*Left*) and to ABM data with falsely registered cells (*Right*). (*A*) Cell distributions at three time points from an ABM simulation. The green cells denote macrophages, the red cells denote viable tumor cells, and the orange cells are necrotic tumor cells. There are ∼100 macrophages and ∼20 falsely registered cells. (*B*) The barcode for each snapshot of the simulation. A bar in the barcode corresponds to a loop formed by the macrophages (green). Longer bars correspond to larger loops. (*C*) 1-PH decay curves tracking the length of the longest bar in the barcode against time for simulations with different chemoattractant sensitivity parameter χ. The curves show the average curve with standard deviation (SD) bands, computed with five simulations for each value of χ. Without noise we separate the distinct chemotaxis parameters. The decay curves are significantly disrupted by the introduction of measurement noise. (*D*) The PCF, g(r) for each snapshot of the simulation. The horizontal dashed line at g(r)=1 is indicative of randomly distributed cells. g(r)>1 suggests clustering of macrophages at distance r from one another; g(r)<1 suggests dispersal. (*E*) Curves tracking the maximum value of the PCF throughout the simulation (mean and SD of five simulations). The curves show that macrophages become less clustered as they begin to infiltrate the spheroid. Curves at high χ show more clustering toward the end of simulations, as macrophages reach the spheroid core and become more clustered again. In the presence of measurement noise, these patterns become less clear and are difficult to interpret. (*F*) MPH decay curves tracking the 2-norm of the MPH landscape against time. The MPH decay curves are less impacted by the measurement error. In *C*, *E*, and *F*, t=0 corresponds to the time at which macrophages are introduced into the simulation.

We generate synthetic data from a two-dimensional (2D), off-lattice, cell-center agent-based model (ABM) that simulates macrophages infiltrating an avascular spheroid in response to a chemotactic gradient (based on ref. 36). Individual tumor cells and macrophages are represented as discrete particles whose behavior is determined by a set of rules. At the spheroid edge, oxygen levels are high, and tumor cells proliferate. As oxygen diffuses into the tumor and is consumed by the cells, its levels decrease, leading to cell death in the core of the spheroid. Under hypoxia, tumor cells release a chemoattractant, which we take to be macrophage colony stimulating factor-1 (CSF-1), that establishes a spatial chemotactic gradient as it diffuses through the spheroid. All cells move in response to the mechanical forces exerted on them by neighboring cells; the macrophages are also subject to chemotactic forces which bias their movement in the direction of increasing chemoattractant levels. A parameter χ describes how sensitive macrophages are to spatial gradients of CSF-1 (when χ = 0, macrophages are insensitive to the chemoattractant). Each simulation is initialized with macrophages distributed around the boundary of a well-developed spheroid (in which the net rate of tumor cell proliferation balances the net rate of tumor cell death). We simulate the ABM for different values of the chemotactic sensitivity parameter χ and record the spatial location of the infiltrating macrophages over time (Fig. 1 *A*, *Left*). Subsequently, we add biologically realistic levels of noise to the data by misclassifying a proportion of the tumor cells as macrophages (Fig. 1 *A*, *Right*).

### From Histology Slides to Point Cloud Data.

Using digitized immunohistochemistry (IHC) images of human head and neck squamous cell carcinoma specimens, we extract the locations of three immune cell types relative to regions of varying oxygen availability present within the tumors using a semiautomated pipeline (34). The cell types studied are cytotoxic T lymphocytes, regulatory T lymphocytes, and macrophages, defined by their respective expression of CD8, FoxP3, and CD68 proteins. The best oxygenated, well-vascularized stromal regions were identified by lack of expression of the epithelial marker pancytokeratin (PanCK). Less well oxygenated areas were identified using a series of hypoxia markers. Regions with an oxygen partial pressure of ∼20 mm Hg were identified by expression of the hypoxia-inducible factor target gene carbonic anhydrase 9 (CAIX). Regions with an oxygen partial pressure of ∼10 mm Hg were identified by staining for adducts of the exogenous hypoxia marker pimonidazole (Pimo). Necrotic regions that are considered virtually anoxic were manually annotated by P.S.M., a pathologist. (See *Materials and Methods* section and *SI Appendix* for further details of IHC protocols, image analysis pipeline, and hypoxia markers.)

## Results

Tumors are heterogeneous, and different regions exhibit variable immune cell densities; their spatial organization opens up avenues for TDA, as we show here.

**Fig. 2.**
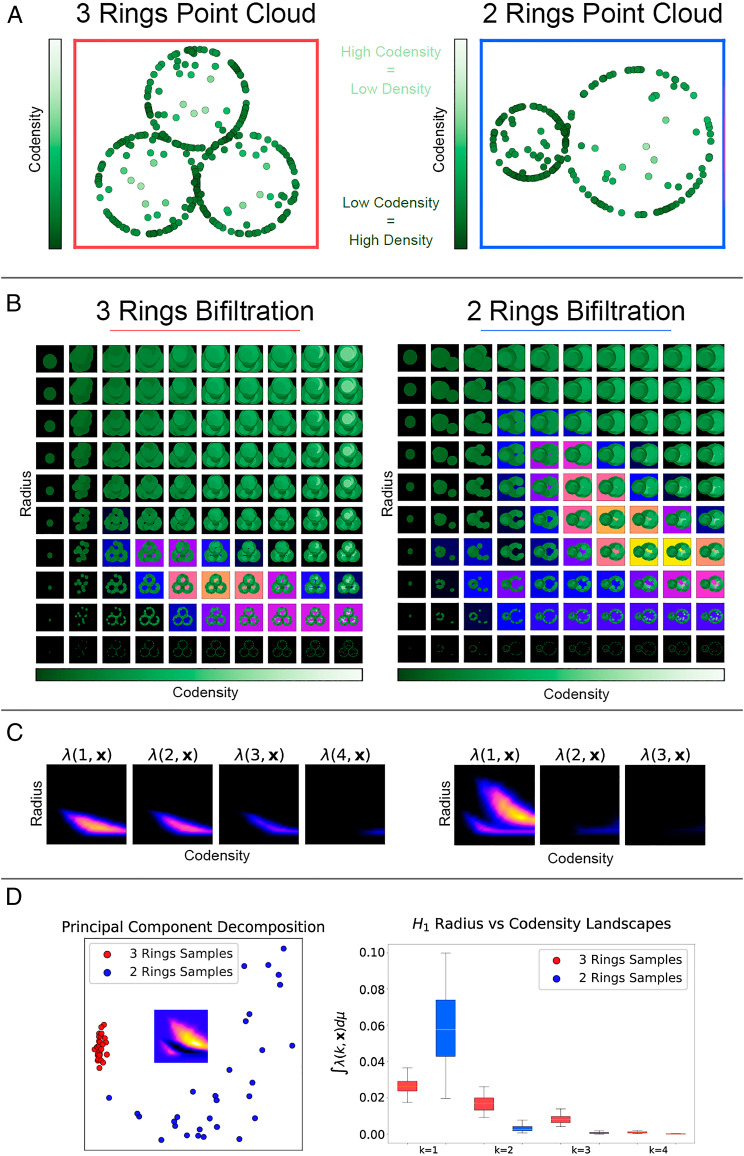
We illustrate the features of point cloud data which may be extracted by MPH and associated landscapes (MPH landscapes). (*A*) Point clouds from rings of different radii, together with measurement noise, to demonstrate that MPH landscapes can detect the size and number of loops in the presence of noise. Eighty points are uniformly sampled from each ring, and 20 points are sampled from the area enclosed by each ring. (*B*) Illustrations of the radius-codensity bifiltrations associated with the example point clouds. Increasing the radius parameter increases the radius of the balls centered at the points of the point cloud. The codensity parameter first admits points from dense regions of the point cloud (dark green) and then includes points from the sparser regions (light green). We highlight the parameter values for which loops are formed in the bifiltration; the strongest signal of a loop is shown in yellow. (*C*) The MPH landscapes for the bifiltrations highlight the regions of the parameter space for which loops are detected. For the 3 Rings point cloud we detect a signal above small radius parameters in $\lambda \left(1,\vec{x}\right),\lambda \left(2,\vec{x}\right)$ and $\lambda \left(3,\vec{x}\right)$ but not $\lambda \left(4,\vec{x}\right)$. These signals correspond to the three small loops of the same scale in the point cloud. For the 2 Rings point cloud we detect a signal above large radius and large codensity parameters and a signal for small radius and small codensity parameters in $\lambda \left(1,\vec{x}\right)$. These signals correspond to the large radius sparsely sampled loop and the small radius densely sampled loop respectively. (*D*) Taking 30 samples of each of the point clouds and treating the output persistence landscapes as high dimensional vectors. PCA of the vectors $\lambda \left(1,\vec{x}\right)$ identifies that the principal difference between the two types of point clouds is the presence of a large radius loop. Taking the norm of the landscape vectors outputs a real value. We plot the distributions of the norms $\|\lambda \left(k,\vec{x}\right)\|,k=1,2,3,4$ for the two point clouds. These distributions identify a large loop in the 2 Rings point clouds and three loops in the 3 Rings point clouds.

1-PH can be used to study spatial distributions of immune cells, giving information that complements traditional statistical methods, such as the pair-correlation function (PCF), as we demonstrate here using data generated by the ABM. Both methods perform well on perfect data but not when (biologically relevant) measurement errors are introduced (Fig. 1 *A*–*E*). The distorting effects of the measurement errors are alleviated when MPH is used instead, as is demonstrated using the ABM data (Fig. 1*F*). Two tailor-made examples (Fig. 2) and an interactive demonstration (35) illustrate interpretability of this mathematically sophisticated method and versatility of the new associated statistical tool: MPH landscapes.

**Fig. 3.**
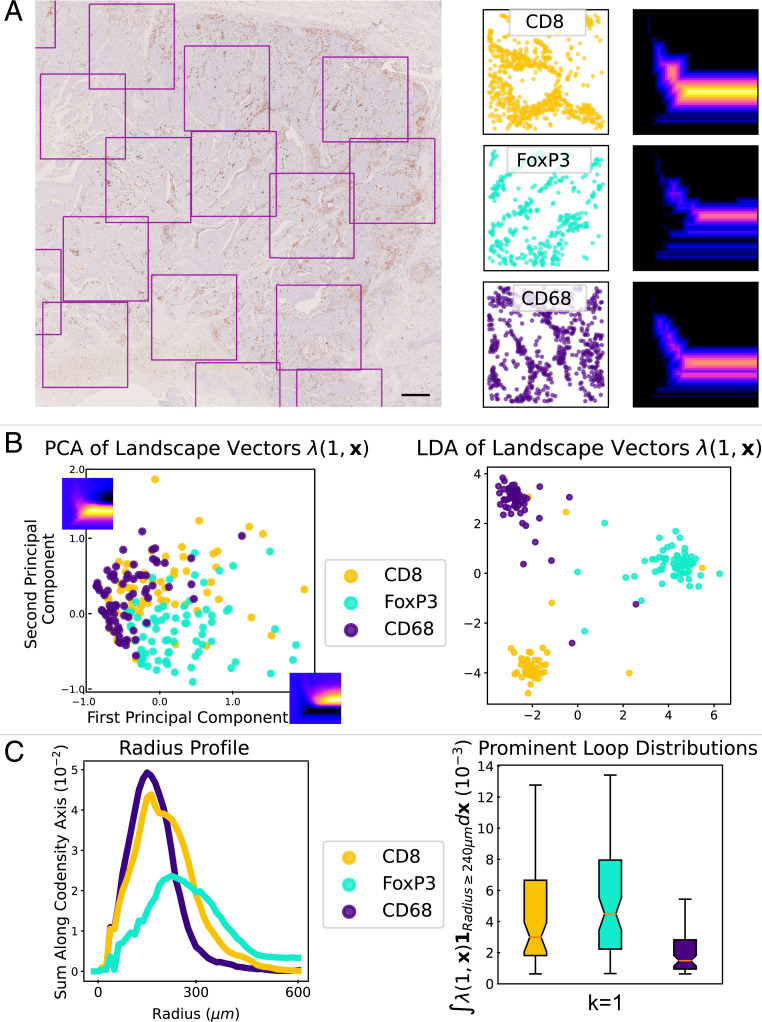
TDA of CD8^+^, FoxP3^+^, and CD68^+^ cell spatial patterning in 1.5 mm × 1.5 mm tumor regions. (*A*) A region of a head and neck tumor IHC slide stained to show CD8^+^ T cells. (Scale bar 500 μm.) 1.5 mm × 1.5 mm regions of interest are highlighted. In this tumor, sufficient tissue was present to sample 67 regions stained for CD8^+^ T cells, 74 regions stained for FoxP3^+^ T cells, and 74 regions stained for CD68^+^ macrophages. For each region of interest we extract a point cloud of immune cell locations. We plot example point clouds and their associated first multiparameter MPH landscape, $\lambda \left(1,\vec{x}\right)$, for the radius-codensity filtration. (*B*) We compute the MPH landscapes for the radius-codensity filtrations associated to each sample. Treating the persistence landscapes as feature vectors we apply PCA to identify the principal difference between the landscapes of the CD8^+^, FoxP3^+^, and CD68^+^ samples. The first and second principal components are plotted on the PCA plot axes. We observe that the T cell samples support loops at large radius parameter values. We use LDA as another dimension reduction technique to visualize the distribution of the landscape vectors and see that the landscape vectors of the three immune cell types can be well separated with a linear classifier. (*C*) We indicate the prevalence of loops for different radius parameters. We sum the average MPH landscapes along the codensity parameter, $\int \lambda \left(1,\vec{x}\right)dx_{\text{codensity}}$, to produce *radius profile* curves for each immune cell type. Again this indicates that the T cell samples support loops with larger radius parameter than the macrophages. We convert the collections of MPH landscapes to real values which detect loops of large radius parameter, by integrating the landscapes over the parameter values with $y_{\text{radius}}\geq 240\mu m$: $\int \lambda \left(1,\vec{x}\right)1_{\text{Radius}\geq 240\mu m}dx_{\text{codensity}}$.

Applying MPH landscapes to a collection of (small) regions of interest across a tumor, distinct spatial patterns for different immune cell types are detected (Fig. 3): in this example, in these regions of interest, T cell (CD8^+^ and FoxP3^+^) distributions tend to form more pronounced voids than macrophage (CD68^+^) distributions, a finding which appears to be more marked in less well oxygenated tumors compared to those with less extensive regions of necrosis, pimonidazole- or CAIX-positivity.

**Fig. 4.**
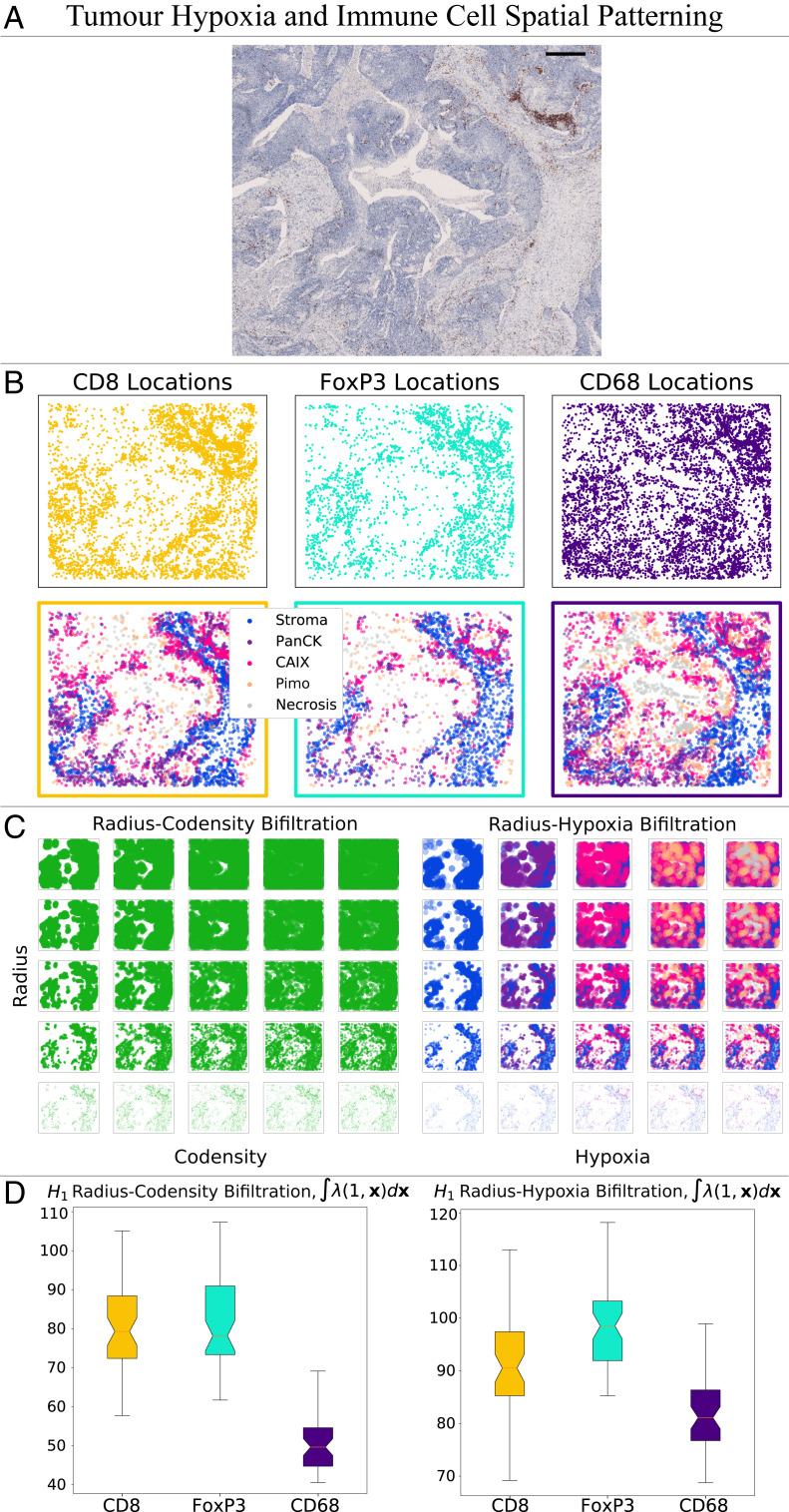
We investigate the distribution of immune cells relative to regions of tumor hypoxia, and compare radius-codensity against radius-hypoxia bifiltrations. (*A*) IHC slide showing CD8^+^ T cells in a head and neck tumor. The region contains a central area of necrosis annotated by a pathologist. Consecutive IHC regions stained for FoxP3^+^ T cells, CD68^+^ macrophages, PanCK (a marker distinguishing tumor from stroma), CAIX (a marker for hypoxia), and Pimo (a marker for more severe hypoxia) were manually aligned. (Scale bar: 500 μm, region size 4.72 mm × 3.96 mm.) (*B*) Point clouds of CD8^+^, CD68^+^, and FoxP3^+^ cells are identified from the regions of interest and colored according to which of five distinct tissue regions they lie in: necrosis (identified via manual annotation), Pimo^+^ (severe hypoxia, identified via positive pimonidazole staining), CAIX^+^ (less extreme hypoxia, identified via positive CAIX staining and negative pimonidazole staining), PanCK^+^ (better oxygenated tumor, identified via positive PanCK staining and negative CAIX and pimonidazole staining), and stroma (PanCK negative tissue). (*C*) This illustrates the similarity between the radius-codensity and the radius-hypoxia bifiltration of one subsample of 1,500 FoxP3^+^ cells from this region. (*D*) To examine the distribution of all three immune cell types in detail we repeatedly sample from the large point cloud of cells surrounding the necrotic region. Taking 50 samples we compare the norm of the MPH landscapes for the radius-codensity bifiltration and the radius-hypoxia bifiltration. The results indicate that these two filtrations give comparable relative values for all three cell types. The radius-codensity and radius-hypoxia bifiltrations both indicate that CD68^+^ cells infiltrate hypoxic regions to a greater extent than CD8^+^ and FoxP3^+^ cells. The data suggest that this cell codensity descriptor is a good proxy for hypoxia level.

Employing MPH landscapes in a different statistical study, over larger tumor areas, suggests a link between immune cell density and oxygen levels; deriving one of the parameters (codensity) automatically from cell distribution data produces a good proxy for tumor hypoxia (Fig. 4).

### Statistical and Topological Analysis of ABMs.

We first analyze the spatial patterns formed by the infiltrating macrophages in the ABM using 1-PH and the PCF from spatial statistics. Both approaches provide more detailed spatial descriptors than cell counting and have been used to analyze histology data (11, 12, 34, 37); we have previously validated the use of PCF to describe macrophage infiltration in human tumors by demonstrating that images grouped semiquantitatively by a pathologist share similar statistics (34). In particular, we quantify the extent to which the macrophages infiltrate the spheroid at each time point in the ABM simulations.

A ubiquitous tool in the field of TDA, 1-PH, has seen a variety of applications to biological data (5, 9, 11, 12, 38–40). Persistent homology encodes nonlinear features of a point cloud in an algebraic object, giving a multiscale, topological summary. In our setting of 2D point clouds, persistent homology can be used to detect loops. 1-PH persistence modules can be visualized as a barcode in which the length of each bar in the barcode reflects the size of a loop detected in the point cloud.

At the beginning of each ABM simulation the macrophages lie on the boundary of the spheroid (Fig. 1*A*). We quantify the macrophage location with perfect simulated data (Fig. 1, *Left*). The 1-PH barcode represents the large loop of macrophages present at the beginning of the simulation with a long bar (Fig. 1*B*). As the simulation advances, the macrophages infiltrate from the boundary to the core of the spheroid and the size of the loop formed by the cells and the corresponding bar in the barcode shrinks.

We now vary the chemoattractant sensitivity parameter χ in our simulations and track the rate at which the length of the longest bar in the barcode in Fig. 1*C* decreases over time. As such, these decay curves quantify the rate at which the macrophages migrate into the center of the spheroid.

Fig. 1*D* shows the PCF g(r) calculated from the macrophage locations at different time steps shown in Fig. 1*A* (see *SI Appendix* for definition). The peak of the PCF at small radii shows that macrophages are initially more likely to be close to other macrophages than if they were randomly distributed. As the simulation advances the peak becomes less pronounced, suggesting that macrophages become less clustered over time in this simulation. Fig. 1*E* tracks the average peak (i.e., maximum value) of the PCF [denoted by maxg(r)] for increasing chemoattractant sensitivity (i.e., different values of χ).

Taken together, Fig. 1 *C* and *E* describe infiltration of macrophages into the spheroid over the simulation (the dynamics described here are consistent with those described in ref. 41). Fig. 1*C* indicates that the chemotaxis sensitivity parameter χ affects the time at which the macrophages penetrate the spheroid boundary. The greater the sensitivity to chemotaxis, the more rapidly the macrophages cross the spheroid boundary, causing both the length of the longest bar in the barcode and the maximum value of the PCF to decrease more quickly. Second, we observe that once the macrophages have penetrated the spheroid, the decay curves in Fig. 1*C* have similar gradients, indicating that the rate at which macrophages proceed to the spheroid core is insensitive to the different chemotaxis sensitivity parameters. In simulations with high chemotaxis sensitivity (large χ), the curves in Fig. 1*E* begin to increase at later time steps as macrophages cluster at the spheroid core. These spatial descriptions give complementary information. In particular, the PCF cannot distinguish between clustering at the simulation start in which macrophages form a loop around the spheroid and clustering at the end in which macrophages are at the spheroid core. This difference can be seen in the 1-PH analysis.

### The Case for MPH.

Our 1-PH analysis of the ABM simulations is sensitive to the location of each macrophage. In the analysis of synthetic data, this sensitivity was an advantage and allowed detection of subtle changes to the model. However, this sensitivity is a less desirable property if we wish to analyze real biological or clinical data. It is likely that in digitized clinical histology samples false positive points will result from inaccuracies during sample preparation and image analysis. With this assumption, we run the same ABM simulation, except at each time step we have ∼100 true macrophages and ∼20 false positives. We investigate the effect of measurement error in the data on 1-PH and PCF (Fig. 1 *A*–*E*, Right). The prominent bars in Fig. 1*B* and the 1-PH decay curves in Fig. 1*C* are significantly diminished by the misidentified cells. Without measurement error the longest bars shorten from ∼13 cell diameters to ∼2 cell diameters and with measurement error from ∼5 cell diameters to ∼2 cell diameters. Similarly, for the PCF analysis, the maximum value of g(r) is decreased in Fig. 1*D*, causing increased noise and overlap in the curves in Fig. 1*E*.

We would like topological summaries for ABM and histology data analyses to be both robust to outlier noise and sensitive to the length scale of the spatial patterns formed by cell point clouds. Our solution is to filter our point clouds by two parameters. Instead of relying on one radial parameter as for 1-PH, we introduce an additional parameter, codensity. The radius parameter serves to detect the size of topological features. The codensity parameter includes cells in dense regions of the point cloud earlier in the filtration and thus adds robustness to noise introduced by less densely arranged outlier cells. MPH is an extension of 1-PH involving a two-parameter filtration, or bifiltration, of data and requiring new theory and computational tools (see *Introducing MPH Landscapes for Applications* and *SI Appendix* for details).

One can view previous 1-PH applications to breast cancer and prostate cancer histology images in refs. 11, 12 as fixing one of our two parameters and varying the other. For example, the technique employed by ref. 12 is analogous to our 1-PH analysis of the ABM data and is notably sensitive to measurement errors. In contrast, the technique of ref. 11 is robust to outlier noise but sacrifices sensitivity to the size of the topological features extracted, in the sense that both small and large loops of the same pixel intensity produce the same topological fingerprint. Furthermore, fixing one of the parameter values requires tuning which is hard to determine. Indeed, the survey article (42) identifies that “…tuning parameters…remains one of the greatest challenges in TDA.” By computing MPH, we circumvent the requirement for tuning and maintain scale information in our methodology. For completeness, we also include in *SI Appendix*, section 3C, a comparison with known 1-PH noise reduction techniques (*SI Appendix*, Tables S2 and S3.)

As a proof of concept, we apply a MPH analysis on the radius-codensity bifiltration of macrophages as the ABM simulations progress. We use MPH landscapes (21) as described in *Introducing MPH Landscapes for Applications*. Like the barcode for 1-PH, the size of the norm of the MPH landscape decreases as the macrophages migrate into the core of the spheroid (Fig. 1*F*). The MPH decay curves are less impacted by the measurement noise, demonstrating that MPH mitigates the impact of mislabeled cells. Hence, without measurement error the landscape norms decay from norm ∼5 to norm ∼0.5 and with measurement error from norm ∼3.5 to norm ∼0.5.

### Introducing MPH Landscapes for Applications.

The topological summaries produced by persistence techniques may be vectorized and, thus, are amenable to traditional data analysis techniques. Persistence landscapes, first introduced in ref. 20, have been widely used as a vectorization for 1-PH (43–45). We have previously extended this work to a vectorization for MPH, facilitating the application of traditional statistical techniques to MPH (21). Real-valued statistics derived from these vectorizations obey a central limit theorem, and hence, it is possible to statistically analyze the topological features of a dataset. Computational feasability and interpretability distinguish MPH landscapes from other vectorization methods for MPH (compare refs. 46, 47).

Here we illustrate how MPH landscapes can detect the number of topological features and their size in the presence of outlier noise (Fig. 2). Fig. 2*A* depicts two example point clouds drawn from distributions that we call “3 Rings” and “2 Rings.” We aim to identify that 3 Rings contains three loops of medium size and that 2 Rings contains one large loop and one small loop. We filter the point clouds by a radius parameter (radius of dots plotted) and codensity parameter (fifth nearest neighbor empirical codensity function detailed in *SI Appendix*). The radius-codensity bifiltration is illustrated in Fig. 2*B*. At parameter value $\left(x_{\text{codensity}},y_{\text{radius}}\right)$ we build the bifiltration at scale parameter $y_{\text{radius}}$ on the points with codensity less than or equal to $x_{\text{codensity}}$. For an a priori unknown range of the $x_{\text{codensity}}$ parameter, the outlier points will be excluded and the loops will be detected.

We use MPH landscapes (21) to vectorize, quantify, and statistically analyze the topological features present in the bifiltered point cloud. The MPH landscapes are a sequence of functions $\lambda \left(k,\vec{x}\right),k=1,2,3\ldots$ (*SI Appendix*, Definition 7). In our setting of two-parameter persistence, we visualize the landscape functions $\lambda \left(k,\vec{x}\right):R^{2}\to R_{\geq 0}$ as a sequence of images, with the color indicating their value. $\lambda \left(k,\vec{x}\right)$ is nonzero only if there are at least k loops present at parameter value $\vec{x}=\left(x_{\text{codensity}},y_{\text{radius}}\right)$. The value $\lambda \left(k,\vec{x}\right)$ indicates the prominence of the $k^{\text{th}}$ most prominent loop at that parameter value: just as long bars in a barcode indicate prominent loops in the point cloud, large landscape values indicate significant loops. We note that it is important that the radius and codensity parameters vary over similar scales (through normalization, if necessary), so that the persistence of topological features is not dominated by the change in one of the parameters. However, there is no need to specify a particular density value.

We depict the MPH landscapes for our examples in Fig. 2*C*. For *2 Rings* we receive a signal above large radius and large codensity parameters and a signal for small radius and small codensity parameters in $\lambda \left(1,\vec{x}\right)$. These signals correspond to one large-radius, sparsely sampled loop and one small-radius, densely sampled loop respectively. For 3 Rings we receive a signal above medium radius parameters in $\lambda \left(1,\vec{x}\right)$, $\lambda \left(2,\vec{x}\right)$, and $\lambda \left(3,\vec{x}\right)$ but not $\lambda \left(4,\vec{x}\right)$. These signals correspond to the three medium loops of the same scale and density in the point cloud.

Qualitative differences in the landscapes can be visualized and tested numerically. We sample 30 point clouds from each of 3 Rings and 2 Rings and compute their MPH landscapes. The MPH landscapes serve as feature vectors for the 60 point clouds. In Fig. 2*D* we plot the 60 feature vectors by their first two principal components together with the first principal component feature vector, illustrating how the landscapes of the two distributions differ and that the feature vectors may be linearly separated.

Taking the norm of the landscape vectors outputs a real value. We plot the distributions of the real values $\left\|\lambda \left(k,\vec{x}\right)\right\|$ for 3 Rings and 2 Rings. The large radius loop in 2 Rings causes the norm of the first landscapes of 2 Rings to be larger than 3 Rings. The three loops of the same size in 3 Rings cause $\left\|\lambda \left(3,\vec{x}\right)\|\gg \|\lambda \left(4,\vec{x}\right)\right\|$ for these landscapes.

Interactive examples of 1-PH and MPH accompanying this work can be found online (35).

### MPH Landscapes Characterize Spatial Patterns of Immune Cells.

Given the prognostic and biological significance of tumor-infiltrating immune cells, their distributions within tumors are of interest to both clinicians and medical researchers. Therefore, we analyze the spatial patterns of CD8^+^, FoxP3^+^, and CD68^+^ cells across whole slide images taken from 16 head and neck tumors. A pathologist (P.S.M.) annotated the tumor regions within each image and nonoverlapping 1.5 mm × 1.5 mm regions of interest were then automatically sampled from within the annotated region until saturation was achieved. Thus, depending upon their size, tumors contained between 2 and 90 regions of interest, our samples. For each sample we construct the radius-codensity bifiltration and compute MPH (see *SI Appendix* for details), using the first MPH landscape ($\lambda \left(1,\vec{x}\right)$) (21) as a feature vector to quantify the spatial patterning. We can then compare the landscape vectors using traditional analysis techniques. An advantage of this approach is that where there are multiple regions of interest (≥50 samples) we can summarize statistically the average behavior across the whole tissue and avoid the risk that biological variation might confound our analysis if we only compared individual samples. Such analysis of a single tumor is summarized in Fig. 3.

Principal component analysis (PCA) of the collection of landscape vectors (Fig. 3*B*) shows that the MPH landscapes capture differences in the spatial patterning of the three cell types. We also observe that CD8^+^ and FoxP3^+^ cell samples contain regions with more prominent voids of larger persistence in both the radius and codensity parameters than the CD68^+^ samples.

We apply linear discriminant analysis (LDA) as a dimension reduction technique (48) to the MPH landscape vectors (Fig. 3*B*). Our LDA projects the MPH landscape vectors into a 2D plane which maximizes the separation between the cell types. We see that the spatial patterning information captured by the MPH landscapes of the three immune cell types is sufficient to separate the samples into clusters corresponding to their cell type.

We test the robustness of the MPH landscape LDA clustering by training an LDA classifier to distinguish the cell types in each sample using the first MPH landscape. For each pair of cell types we make a randomized 80/20 training/test split, train a regularized LDA linear classifier on the training data, and evaluate the classification accuracy on the test data. Repeating this process 100 times we attain average pairwise classification accuracies: CD8^+^ vs. FoxP3^+^ 74.7%, CD8^+^ vs. CD68^+^ 65.3%, and FoxP3^+^ vs. CD68^+^ 86.3% (see tumor $T_{C}$ in *SI Appendix*, Table S10). Using both the first and second MPH landscapes ($\lambda \left(1,\vec{x}\right),\lambda \left(2,\vec{x}\right)$) marginally improves these classification accuracies.

In Fig. 3*C* we plot *radius profiles* for each immune cell type: we compute the mean first MPH landscape across the 1.5 mm × 1.5 mm regions and sum these landscapes along the codensity parameter [$\int \lambda \left(1,\vec{x}\right)dx_{\text{codensity}}$] to produce the radius profile. The radius profile coarsely summarizes the size of the voids formed by each immune cell type in the tumor. We see that the FoxP3^+^ (and to a lesser extent CD8^+^) cells support voids at larger radius than CD68^+^ cells in this tumor.

We also sum the MPH landscapes over the range of large radius parameters so that each sample produces an R-valued statistic, $\int_{y_{\text{radius}}>240}\lambda \left(1,\vec{x}\right)d\vec{x}$, which indicates the presence of large voids formed by the cells. The boxplots in Fig. 3*C* display the distributions of these R-valued statistics for a tumor which showed extensive hypoxia across the panel of markers.

For this specimen, we perform a one-sided permutation test with null hypothesis that the mean of the R-valued statistics coincide for the group of CD8^+^ samples and FoxP3^+^ samples against the group of CD68^+^ samples. The statistical test indicates the mean of the macrophage distribution is less than the means of the CD8^+^ and FoxP3^+^ distributions, which is consistent with our observation that CD8^+^ and FoxP3^+^ cells show voids with large persistence in both the radius and codensity parameters in contrast to the CD68^+^ cells (see tumor $T_{C}$ in *SI Appendix*, Fig. S9 and Tables S4 and S7 ).

Our cohort contains five tumors which are sufficiently large for us to extract ≥50, 1.5 mm × 1.5 mm regions and thus to derive robust statistical summaries of the average behavior of each immune cell type across the tumor as a whole (*SI Appendix*, Tables S4 and S7). These tumors are heterogeneous, showing varying extents of hypoxia. Within all tumors, the loops formed by the FoxP3^+^ cells have larger radii than the other cell types, but this is not significant for the best oxygenated tumor. We summarize the topological analysis and expression of the hypoxia markers for the entire cohort in *SI Appendix*.

### Codensity MPH as a Biomarker for Hypoxia.

We next investigate the relationship between the location of specific immune cells and the local oxygen levels. The location and spatial patterning of cell types can be analyzed in small regions, whereas oxygen levels can change over longer length scales. Alignment of cell locations and oxygen levels at larger regions poses challenges with registration.

In this analysis, we focus on a larger region of interest, manually selected for the presence of central necrosis, containing $∼10^{4}$ immune cells of each type (Fig. 4*A*). Using immunohistochemical and morphological markers we classify the oxygen environment of each cell, in order of increasing hypoxia: *Stroma*, *PanCK*, *CAIX*, *Pimo*, and *Necrosis* (Fig. 4*B*). Analyzing the corresponding large point clouds with MPH techniques poses computational challenges. Therefore, we use a bootstrap resampling technique, taking 50 subsamples of 1,500 points from the large point clouds (49). For each subsampled point cloud we construct radius-codensity and radius-hypoxia bifiltrations (Fig. 4*C*), compute the first MPH landscapes, and then analyze the distribution of the integrals of the MPH landscapes for the three cell types (Fig. 4*D*). Our MPH analysis (of the hypoxic and better oxygenated tumor) shows CD68^+^ cells infiltrate hypoxic tumor regions to a greater extent than the CD8^+^ cells (Fig. 4 and *SI Appendix*, Fig. S12), supporting previous observations (50). In this analysis the distributions of CD8^+^ and FoxP3^+^ T cells are very similar, and for these cell types, the codensity parameter appears to be a good proxy for hypoxia (*SI Appendix*, Fig. S13).

## Discussion

In this paper, we have introduced, implemented, and applied MPH landscapes, a statistical multiparameter topological analysis, to synthetic and clinical data of immune cell–tumor interactions. This tool is robust to noise, outliers, and artifacts, which is desirable for biological datasets. Moreover MPH landscapes provides a quantitative descriptor across multiple length scales and can be integrated with data analysis and machine learning techniques (20). Thus, we can sidestep the standard spatially averaged measures and surpass limitations of 1-PH.

We showcased the power of MPH landscapes on immune cell patterns by computing them on multiple samples of noisy, mislabeled data generated from an ABM, which quantified the effect of chemotaxis on immune cell infiltration. Furthermore, clinical head and neck cancer histology data provided a testbed to highlight the utility of MPH landscapes. Specifically, the LDA classified the cells into clusters corresponding to their cell type, and the MPH landscapes quantified known differences between immune cell patterns and allowed us to investigate the relationship between immune cell location and tumor hypoxia. Although we require more extensive data for statistical statements (e.g., more small samples and a larger cohort), the radius-codensity MPH landscapes appear to be a good proxy for tumor hypoxia. Based on these positive findings, we suggest that future work exploring the relationship between topological analyses, biological mechanisms, clinical/experimental interventions, and outcomes is warranted. Although computational registration of multiple sections cut from the same tissue block is an established methodology to generate multilabeled output images, the work presented in this manuscript would be improved by multiplex imaging of multiple markers on a single tissue section. Our pipeline can be easily adapted to analyze such data and future work will focus on images generated with this approach.

The real advantages and limitations of any technique become apparent once their performance can be evaluated in real-world applications, which is particularly true for MPH landscapes. Despite the increase in computational costs of MPH landscapes compared to 1-PH, we circumvented this by analyzing many small samples (Fig. 3) or subsampling immune cells from a larger region (Fig. 4). Therefore, we highlighted the versatility of MPH landscapes for simultaneously providing statements, on average, about the different densities and shape distributions of tumor-infiltrating immune cells in head and neck cancer. Furthermore, we illustrated that including a second filtration parameter (e.g., either codensity or hypoxia) to the radius parameter enables this technique to overcome anomalies that arise in digital pathology. In future, MPH landscapes may be applied to understand more complex data from a wide range of modeling frameworks (e.g., stochastic models), as well as other experimental modalities.

## Materials and Methods

All point cloud data are available at https://github.com/MultiparameterTDAHistology/SpatialPatterningOfImmuneCells.

### ABM.

All simulations were performed using the open source Chaste framework (51). Simulations are initialized with a well-developed spheroid at its equilibrium size and composition and 100 macrophages distributed randomly in contact with the spheroid edge. We observe the x,y coordinates of the macrophages every 4 time units for a duration of 100 time units. For observations with noise, at each observation, every tumor cell (viable or necrotic) is mislabeled as a macrophage with probability 0.01.

### IHC Data.

The (x,y) coordinates for each immune cell are identified by using an image analysis pipeline, implemented in MATLAB, that combines superpixellation and pathologist-trained support vector machine classifiers to identify positively stained cells from IHC images. We then use a modified watershedding algorithm to identify immune cell centers and extract a 2D point cloud for each image (see https://github.com/JABull1066/ImageAnalysisScripts). This pipeline has been validated against both humans and other image analysis software and identifies immune cells within IHC images with a comparable accuracy to trained pathologists (34).

### Spatial Statistics.

We apply the PCF, g(r), to point cloud data. The PCF identifies clustering or dispersal in point patterns; g(r)>1 implies clustering at length scale r, and g(r)<1 implies dispersal at length scale r. Following ref. 34, we consider the maximum of g(r), maxg(r), as a summary of the PCF which can be interpreted as identifying the maximum intensity of clustering in the point cloud.

### TDA.

1-PH calculations were performed using the Dionysus 2 software package (https://mrzv.org/software/dionysus2/). MPH calculations were performed using the RIVET software package (https://rivet.readthedocs.io/en/latest/) (15). MPH landscapes were computed using https://github.com/OliverVipond/Multiparameter_Persistence_Landscapes (21). All persistence modules were computed over $Z_{2}$ coefficients. Statistical tests were performed using the Python package scipy.stats (https://www.scipy.org/).

## Supplementary Material

Supplementary File

## Data Availability

Anonymized point cloud data have been deposited in GitHub (https://github.com/MultiparameterTDAHistology/SpatialPatterningOfImmuneCells).
