# Supplementary material for: Multiparameter persistent homology landscapes identify immune cell spatial patterns in tumors
Source: Proc Natl Acad Sci U S A. 2021 Oct 8;118(41):e2102166118. doi: 10.1073/pnas.2102166118 (PMC8522280; doi:10.1073/pnas.2102166118)
Supplement: Supplementary File [file pnas.2102166118.sapp.pdf]

PNAS
www.pnas.org

Supplementary Information for

Multiparameter persistent homology landscapes identify immune cell spatial patterns in tumors

Oliver Vipond, Joshua A Bull, Philip S Macklin, Ulrike Tillmann, Christopher W Pugh, Helen M Byrne, Heather A Harrington

To whom correspondence should be addressed. E-mail:ulrike.tillmann@maths.ox.ac.uk, chris.pugh@ndm.ox.ac.uk, byrne@maths.ox.ac.uk, harrington@maths.ox.ac.uk

This PDF file includes:

Supplementary text
Figs. S1 to S13
Tables S1 to S10
References for SI reference citations

Contents

1 Data. 2
A Agent-based Model (ABM) 2
B Head and Neck Clinical Histology Images 8

2 Analysis Techniques. 9
A Spatial Statistics 9
B Topological Techniques 9

3 Data Analysis. 17
A Multiparameter Persistence Examples 17
B Single Parameter Persistence for Simulation Data 19
C Comparison to 1PH Noise Reduction Techniques 21
D Quantifying Immune Cell Infiltration 21

Supporting Information Text

1. Data.

We use two sources of data: synthetic data generated from an agent-based model (ABM) describing macrophage infiltration into avascular tumor spheroids, and clinical data from digitized immunohistochemistry (IHC) slides of human head and neck tumors. We explain below how each dataset was generated. First we introduce the ABM, then we describe the protocol used to stain and image the IHC slides, and outline the image analysis process used to extract point clouds from the digitized IHC images.

A. Agent-based Model (ABM). We use the open source software Chaste (Cancer, Heart and Soft Tissue Environment) (1, 2) to extend a two-dimensional, hybrid ABM for the growth of multicellular tumor spheroids (3) to simulate their infiltration by macrophages. The simulations are based on *in vitro* experiments designed to investigate the effect of chemotaxis on macrophage infiltration into tumor spheroids (4). The key features of our ABM are summarized in Figure S1, which is adapted from (3).

We distinguish two cell types in our ABM: *tumor cells* and *macrophages*. The behavior of the tumor cells is affected by the local concentration of oxygen, ω (see Equation (1)). Macrophages are not directly affected by the local oxygen concentration; they move along spatial gradients of a chemoattractant, c, which is produced by tumor cells under hypoxia (see Equation (2)). For simplicity, we assume that the macrophages do not inhibit or promote tumor cell growth.

We use an off-lattice ABM in which each cell is represented by its cell center. Cell movement is determined by applying a force balance to each cell (Figure S1B-C indicate the forces that act on tumor cells and macrophages respectively). Cell-cell interactions are modeled by assuming that a spring connects the centers of cells within a specified interaction radius (see Equation (8)).

Oxygen and chemoattractant concentrations. Reaction-diffusion equations describe the concentrations of oxygen $\omega(\mathbf{x}, t)$ and a hypoxia-induced chemoattractant $c(\mathbf{x}, t)$. While multiple chemoattractants, such as macrophage colony-stimulating factor 1 (CSF-1) or chemokine ligand 2 (CCL2)(5), may bias macrophage movement, here, without loss of generality, this chemoattractant is taken to be CSF-1. The centers of viable tumor cells act as point sinks for oxygen, modeling oxygen consumption, while the centers of hypoxic tumor cells act as point sources for the diffusible macrophage chemoattractant. As the timescale of diffusion for oxygen and CSF-1 (seconds) is much faster than the timescale of cell proliferation (hours), we make the standard quasi-steady state assumption (see, e.g., (6)) and consider the following dimensionless equations for ω and c:

$$0 = D_\omega \nabla^2 \omega - \kappa\omega \sum_i \delta(\mathbf{x} - \mathbf{x}_i), \quad \text{for } \mathbf{x} \in \Omega. \qquad [1]$$

$$0 = D_c \nabla^2 c + \kappa_c \sum_i \delta(\mathbf{x} - \mathbf{x}_i)\mathcal{H}(\omega_\text{h} - \omega(\mathbf{x}_i)), \quad \text{for } \mathbf{x} \in \Omega. \qquad [2]$$

In Equation (1) and Equation (2), D_ω and D_c are non-negative diffusion coefficients for oxygen and CSF-1 respectively, κ is the rate at which viable cells consume oxygen, κ_c is the rate at which CSF-1 is produced by hypoxic tumor cells and $\mathbf{x}_i$ is the location of viable cell i. We denote by $\delta(\mathbf{x})$ the delta function ($\delta(\mathbf{x}) = 1$ when $\mathbf{x} = 0$; $\delta(\mathbf{x}) = 0$ otherwise),

and Ω is a square domain which fully encloses the spheroid. $\mathcal{H}$ is the Heaviside step function ($\mathcal{H}(\omega_\text{h} - \omega) = 1$ if $\omega < \omega_\text{h}$; $\mathcal{H}(\omega_\text{h} - \omega) = 0$ otherwise).

Equation (1) and Equation (2) are solved subject to Dirichlet boundary conditions ($\omega = \omega_\infty$ on $\delta\Omega$ and $c = 0$ on $\delta\Omega$) and suitable initial conditions ($\omega = \omega_\infty$ and $c = 0$ in Ω at $t = 0$). We assume that oxygen is maintained at a constant level, ω_∞, in the culture medium which surrounds the tumor spheroid and, by continuity, that on the spheroid boundary the oxygen concentration is also maintained at this constant value. In Equation (2), we account for diffusion of CSF-1 and its production by hypoxic tumor cells. We assume that chemoattractant removal at the boundary of the domain is the dominant sink of chemoattractant and, therefore, as stated above, we fix $c = 0$ there. In particular, we neglect natural decay of CSF-1 in the domain of interest; this could be modeled by including a decay term in Equation (2), and replacing the Dirichlet boundary conditions by zero-flux Neumann boundary conditions.

Equation (1) and Equation (2) are solved numerically on a regular tetrahedral 2D finite element mesh spanning Ω. Figure S1A shows the distribution of CSF-1 across the spheroid at the timestep shown, with the highest concentration of chemoattractant colocalizing with hypoxic tumor cells.

Tumor cell phenotypes. We account for the effect the local oxygen concentration has on the behavior of the tumor cells by introducing the following four phenotypes (see also Figure S1D):

- If $\omega > \omega_q$, then a tumor cell **proliferates**.
- If $\omega \leq \omega_\text{q}$, then the tumor cell becomes **quiescent** and immediately pauses its cell cycle (and conversely).
- If $0 \leq \omega_\text{h} \leq \omega \leq \omega_\text{q}$, then the tumor cell immediately becomes **hypoxic** (and conversely). If a cell remains hypoxic for longer than $\tilde{\tau}_i$ hours then it becomes **necrotic** (7).
- **Necrotic** cells are dead, and do not consume oxygen, although they occupy space for a fixed time period, $\bar{\tau}$ hours, before being removed from the simulation.

Tumor cell proliferation and death. Each viable tumor cell contains two subcellular variables: its *cell cycle time* T_i determines when it proliferates; its *hypoxia time* $\tilde{T}_i$ determines whether it has been hypoxic for long enough to become necrotic. Both subcellular variables increase at rates which depend on the local oxygen concentration. Pseudocode describing how the cell cycle is updated is presented in Algorithm 1.

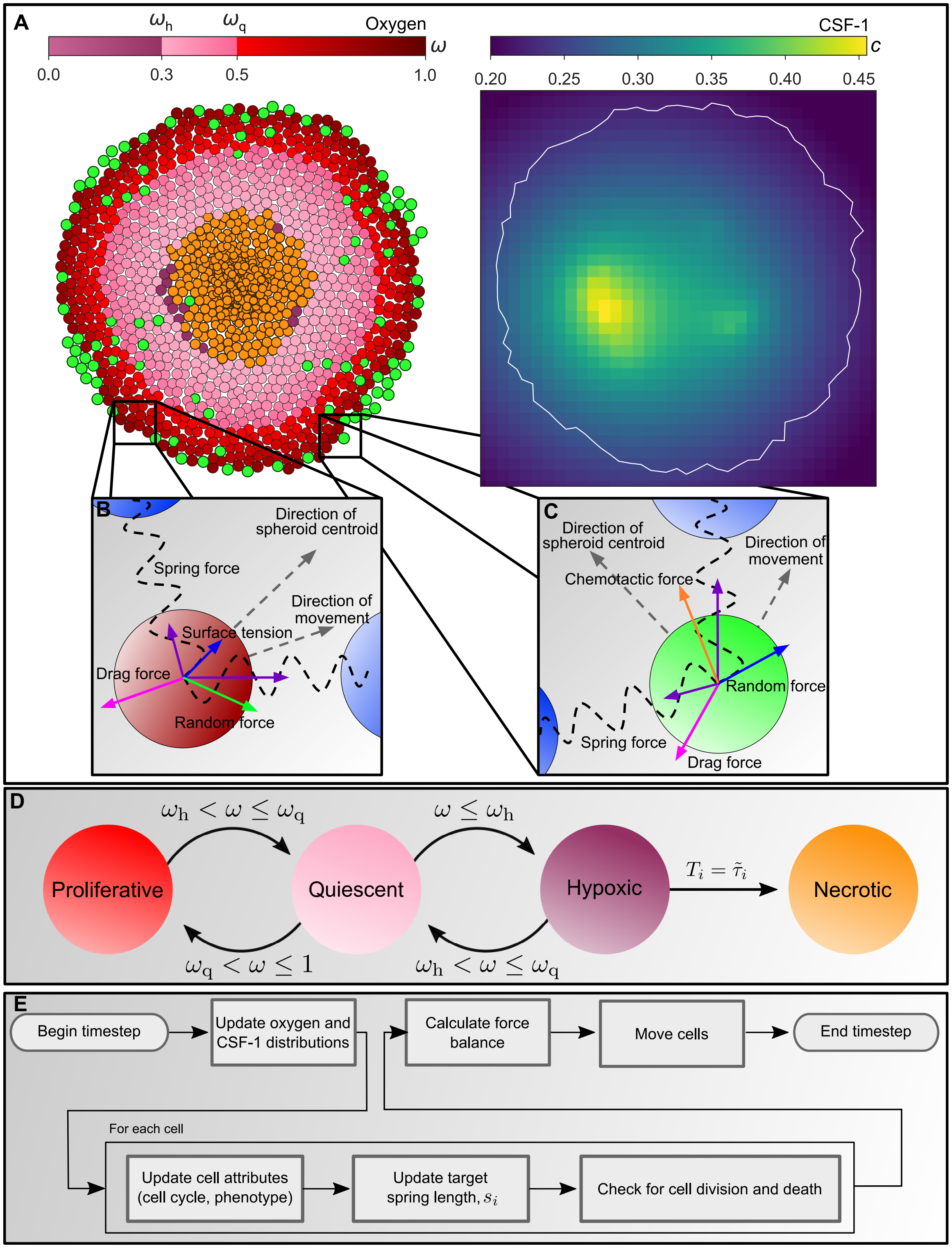

Fig. S1. Schematic of the multiscale, agent-based model (ABM) used to simulate the growth of multicellular tumor spheroids and their infiltration by macrophages.
A: Left - snapshot from an ABM simulation, Right - corresponding distribution of colony stimulating factor 1 (CSF-1) at this timestep. Tumor cells are characterized by their spatial location and local oxygen concentration, ω: proliferating cells (dark red) exist in oxygen-rich regions, where $\omega_q \leq \omega \leq 1$; quiescent cells (pink) are non-proliferating, viable cells which exist in moderate oxygen levels, where $\omega_h \leq \omega < \omega_q$; hypoxic cells (purple) are non-proliferating, viable cells that become necrotic if they remain in low oxygen regions where $\omega \leq \omega_h$, for longer than a prescribed time period; necrotic cells (orange) degrade over time. Macrophages (green) move by chemotaxis up spatial gradients of CSF-1, c, which is produced by hypoxic tumor cells (right, with spheroid outline shown in white). B&C: Schematic indicating the forces which act on individual tumor cells (B) and macrophages (C) and drive their movement. All cells experience: spring forces, due to cell-cell interactions with their neighbors; a random force, which represents fluctuations in the local environment; and a drag force, which resists cell movement. Tumor cells on the spheroid boundary also experience a surface tension force which is directed radially inwards, towards the spheroid centroid, and maintains spheroid compactness (see B). Macrophages experience a chemotactic force, which points in the direction of increasing levels of CSF-1, c (see C). D: Schematic showing how tumor cell phenotype changes in response to the local oxygen concentration, ω. E: Flowchart summarizing how the ABM is updated on each timestep. Pseudocode describing the tumor cell cycle can be found in Algorithm 1.

Algorithm 1 Pseudocode outlining the procedure used to update the cell cycle for tumor cells.

```
Input: All tumor cells, viable or necrotic
for All tumor cells do
    if Cell is alive then
        if $\omega_q < \omega \leq 1$ then
            // Cell is proliferative
            // Move cell through cell cycle by one timestep
            Set $T_i = T_i + dt$ // Ensure hypoxia timer is unset
            Set $\tilde{T}_i = 0$
            // If cell is less than one hour old, increase the cell radius
            if $T_i < 1$ then
                Set $s_i = s_i + R_{\text{Cell}} dt$
            end
            // If cell is at end of cell cycle, proliferate
            if $T_i = \tau_i$ then
                Choose random location within $R_{\text{int}}$ of cell $i$
                Place daughter cell $j$ in selected location
                Set $s_i = \frac{R_{\text{Cell}}}{2}$
                Set $s_j = \frac{R_{\text{Cell}}}{2}$
                Set $T_i = 0$ for cells $i$ and $j$
                Choose new cell cycle durations $\tau_i$ for cells $i$ and $j$
            end
        else if $\omega_h < \omega \leq \omega_q$ then
            // Cell is quiescent
            // Ensure hypoxia timer is unset
            Set $\tilde{T}_i = 0$
        else if $\omega \leq \omega_h$ then
            // Cell is hypoxic
            // Increment hypoxia timer by one timestep
            Set $\tilde{T}_i = \tilde{T}_i + dt$
            // Check for cell death
            if $\tilde{T}_i = \tilde{\tau}_i$ then
                Mark cell as dead
            end
        end
    else
        // Cell is necrotic
        // Reduce necrotic cell radius linearly over $\bar{\tau}$ hours to model decay
        Set $s_i = s_i - \frac{R_{\text{Cell}} dt}{\bar{\tau}}$ if $s_i = 0$ then
            Remove cell from simulation
        end
    end
end
```

At birth, the cell cycle time of tumor cell i is initialized so that $T_i = 0$, and the cell is assigned a cell cycle duration τ_i chosen from a uniform distribution $U(0.75\,\tau, 1.25\,\tau)$, where τ defines the average cell cycle length. Thereafter, T_i evolves as follows:

$$\frac{dT_i}{dt} = \mathcal{H}(\omega(\mathbf{x}_i, t) - \omega_{\text{q}}) \qquad [3]$$

where $\mathcal{H}$ is the Heaviside step function ($\mathcal{H}(\omega - \omega_{\text{q}}) = 1$ if $\omega > \omega_{\text{q}}$; $\mathcal{H}(\omega - \omega_{\text{q}}) = 0$ otherwise). When $T_i = \tau_i$, the cell divides. One daughter cell is located at the same site as its parent, the other is placed at a distance of half a cell diameter away from the parent cell center, in a randomly chosen direction. Both daughter cells are assigned new cell cycle durations, their cell cycle times are set to 0, and thereafter evolve according to Equation (3). The spring length of newborn cells is initially half that of other cells, and grows linearly over the course of 1 hour until it reaches the natural spring length of the parent cell (for details, see description of mechanical forces below).

Each cell has an internal hypoxia time, $\tilde{T}_i$, which evolves as follows:

$$\frac{d\tilde{T}_i}{dt} = \mathcal{H}(\omega_\mathrm{h} - \omega(\mathbf{x}_i, t)), \tag{4}$$

with $\tilde{T}_i = 0$ at the onset of hypoxia. If the local oxygen concentration increases so that $\omega(\mathbf{x}_i, t) > \omega_\mathrm{h}$ then we re-set $\tilde{T}_i = 0$. Tumor cells become necrotic if they remain hypoxic for longer than a threshold time $\tilde{\tau}_i$, where $\tilde{\tau}_i$ is drawn from a uniform distribution $U(0.75\,\tilde{\tau}, 1.25\,\tilde{\tau})$ when a cell is born, and $\tilde{\tau}$ represents the average time a cell can remain viable under hypoxia. Necrotic cells are not viable and do not progress through the cell cycle. They occupy space, but their size reduces over a period of $\bar{\tau}_i$ hours and then they are removed from the simulation. We explain below how size reduction is implemented.

Force balances for tumor cells and macrophages. As indicated in Figure S1, three forces act on the tumor cells: mechanical forces ($\mathbf{F}_i^m$) caused by cell-cell interactions, random forces ($\mathbf{F}_i^r$) which represent fluctuations in the local environment, and surface tension forces ($\mathbf{F}_i^s$) which maintain compactness of the spheroid. In addition to mechanical forces and random forces, macrophages are subject to a chemotactic force ($\mathbf{F}_i^\chi$), which biases their movement up spatial gradients in the chemoattractant CSF-1, c. We assume that macrophages are not subject to surface tension forces. The equations of motion for tumor cells and macrophages derive from Newton's second law, in the over-damped limit, when inertial effects are neglected. The force balances for cell i and macrophage j are given by:

$$\text{Tumor cells:} \quad \nu \frac{d\mathbf{x}_i}{dt} = \mathbf{F}_i^m + \mathbf{F}_i^r + \mathbf{F}_i^s. \tag{5}$$

$$\text{Macrophages:} \quad \nu \frac{d\mathbf{x}_j}{dt} = \mathbf{F}_j^m + \mathbf{F}_j^r + \mathbf{F}_i^\chi. \tag{6}$$

In Equation (5) and Equation (6), the drag force acting on tumor cell i (or macrophage j) is assumed to be proportional to its velocity, with constant of proportionality ν. Functional forms for $\mathbf{F}_i^m$, $\mathbf{F}_i^r$, $\mathbf{F}_i^s$ and $\mathbf{F}_i^\chi$ are defined below.

Mechanical forces, $\mathbf{F}_i^m$ (tumor cells and macrophages) Mechanical forces act on tumor cells and macrophages; for simplicity we refer to their cell centers as "nodes". Node j exerts a mechanical spring force on node i (and vice versa) if the distance between their centers is less than a fixed value, R_int. Following the overlapping spheres approach outlined in (3, 8–11), if $|\mathbf{x_i} - \mathbf{x_j}| < R_\mathrm{int}$ then the force acts in the direction of the vector between the nodes. The magnitude of the force depends on the distance between the associated cells and their sizes. Although cells in our ABM are represented as points, each cell has an associated size which is implicitly implemented by adjusting the resting spring length s_i for each node i. The resting spring length between two nodes, $s_{i,j}$, is the sum of the equilibrium spring lengths for each node ($s_{i,j} = s_i + s_j$). If the distance between two cell centers is larger than $s_{i,j}$ then the nodes experience an attractive force; otherwise, the force is repulsive. The mechanical force, $\mathbf{F}_{i,j}^m$, between nodes i and j, at locations $\mathbf{x}_i$ and $\mathbf{x}_j$, has the form:

$$\mathbf{F}_{i,j}^m = \begin{Bmatrix} \mu s_{i,j} \log\left(1 + \dfrac{x}{s_{i,j}}\right) \dfrac{\mathbf{x}_i - \mathbf{x}_j}{|\mathbf{x}_i - \mathbf{x}_j|} & \text{if } x < 0 \text{ (Repulsive)} \\ \mu x s_{i,j} \exp\left(-\lambda \dfrac{x}{s_{i,j}}\right) \dfrac{\mathbf{x}_i - \mathbf{x}_j}{|\mathbf{x}_i - \mathbf{x}_j|} & \text{if } x \geq 0 \text{ (Adhesive)} \end{Bmatrix} \tag{7}$$

where $x = |\mathbf{x}_i - \mathbf{x}_j| - s_{i,j}$ is the overlap between cells i and j, the parameter $\mu > 0$ represents the spring stiffness and the parameter $\lambda > 0$ determines the strength of cell-cell adhesion. Following Bull *et al.* (3), the net mechanical force acting on a node i at location $\mathbf{x}_i$ is the sum of the contributions of all nodes j within radius R_int:

$$\mathbf{F}_i^m = \sum_{\{j \;|\; |\mathbf{x}_i - \mathbf{x}_j| \leq R_\mathrm{int}\}} \mathbf{F}_{i,j}^m. \tag{8}$$

With the exception of newborn and necrotic cells, we assume that $s_i = R_\mathrm{Cell}$ for tumor cells and macrophages. For convenience, all lengths in our ABM are scaled with respect to this lengthscale, assuming that 1 cell diameter $= 2R_\mathrm{Cell} = 20\mu$m.

Following division, daughter cells are initially assumed to be smaller than their parent cells and, so, we set $s_i = \frac{R_\mathrm{Cell}}{2}$ for both daughter cells. Their spring lengths increase linearly over a period of one hour until $s_i = R_\mathrm{Cell}$. Similarly, we represent the gradual decay of necrotic cells by decreasing their spring lengths linearly to zero over a period of $\bar{\tau}_i$ hours and then remove them from the simulation.

Random forces, $\mathbf{F}_i^r$ (tumor cells and macrophages) The random force acting during the timestep dt is given by:

$$\mathbf{F}_i^r = \sqrt{2Ddt}\,\xi. \qquad [9]$$

In Equation (9), $D > 0$ is a random motility coefficient and $\xi = (\xi_x, \xi_y)$ where ξ_x and ξ_y are random variables drawn from a standard normal distribution.

Surface tension forces, $\mathbf{F}_i^s$ (boundary tumor cells) The surface tension force, $\mathbf{F}_i^s$, experienced by tumor cells on the spheroid boundary has the form:

$$\mathbf{F}_i^s = -\beta\hat{\mathbf{x}}_i, \qquad [10]$$

where the unit vector $\hat{\mathbf{x}}_i$ points from boundary cell i to the spheroid centroid, and parameter $\beta > 0$ determines the strength of the surface tension force. Boundary cells are those belonging to the α-shape of the set of tumor cell centers, where $\alpha = R_{\text{Cell}}$ (12).

Chemotactic forces, $\mathbf{F}_i^\chi$ (macrophages) Following (8), the chemotactic force experienced by macrophage i is given by:

$$\mathbf{F}_i^\chi = \chi\nabla c(\mathbf{x}_i, t), \qquad [11]$$

where the parameter $\chi > 0$ determines the macrophage sensitivity to the chemotactic gradient of CSF-1.

Simulation protocol. Following Bull *et al.* (3), simulations are initialized by uniformly distributing 300 tumor cells within a circle of radius 5 cell diameters. All tumor cells are initially assigned a cell cycle time T_i from a uniform distribution $U(0, 0.75\,\tau)$ to ensure that cell cycles are not synchronized. After 300 hours of spheroid growth, 100 macrophages are distributed randomly around the spheroid edge and the simulation continued for a further 100 hours.

Table S1. Parameter values and ranges used for ABM simulations.

Symbol	Parameter	Dimensionless value	Dimensional range	Refs
dt	Timestep	1/120	1/200 - 1/100 (hours)	(13)
R_{Cell}	Radius of a cell	0.5	7 - 12 (μm)	(14)
R_{int}	Radius of interaction	1.5	21 - 36 (μm)	(13)
α	Radius used to determine α-shape	0.5	7 - 12 (μm)	$*$
ω_∞	Oxygen boundary value	1.0	100 - 150 (mm Hg)	(15, 16)
ω_{h}	Hypoxia threshold	0.1 - 0.7	10 (mm Hg)	(16)
ω_{q}	Quiescence threshold	0.3 - 0.7	30 - 70 (mm Hg)	(3), $*$
τ	Average cell cycle length	8 - 32	13 - 32 (hours)	(17, 18)
τ_i	Cell cycle duration for cell i	0.75τ- 1.25τ	9.75 - 40 (hours)	(3), $*$
$\tilde{\tau}$	Average critical hypoxic duration	8 - 16	Assumed (hours)	(3), $*$
$\tilde{\tau}_i$	Critical hypoxic duration for cell i	0.75$\tilde{\tau}$- 1.25$\tilde{\tau}$	Assumed (hours)	(3), $*$
$\bar{\tau}$	Average necrosis duration	48	Assumed (hours)	(3), $*$
$\bar{\tau}_i$	Necrosis duration for cell i	0.75$\bar{\tau}$- 1.25$\bar{\tau}$	Assumed (hours)	(3), $*$
ν	Damping coefficient	1	0.4 ($N\,\text{s}^{-1}\text{m}^{-1}$)	(13, 19)
μ	Spring constant	45.0	3 - 50 (μg $\text{Cell diameter}^{-1}\,\text{hours}^{-2}$)	(13, 20)
μ_{bead}	Spring constant for macrophages	45.0	3 - 50 (μg $\text{Cell diameter}^{-1}\,\text{hours}^{-2}$)	(3), $*$
λ	Intercellular adhesion scaling coefficient	5.0	Assumed (-)	(13)
s_i	Radius of cell i at equilibrium	R_{Cell}	7 - 12 (μm)	(14)
$s_{i,j}$	Resting spring length between cells i and j	s_i + s_j	0 - 24 (μm)	(3), $*$
D	Random motility coefficient	0.01	Assumed ($\text{Cell diameter}^2\,\text{hours}^{-1}$)	(3), $*$
κ	Oxygen consumption rate	0.03	20×10^{-18} (mol/(cell s))	(21)
D_ω	Oxygen diffusion coefficient	1	1,750 ($\mu\text{m}^2\,\text{seconds}^{-1}$)	(21)
β	Surface tension coefficient	5	Assumed ($\mu\text{g}\,\text{hours}^{-2}$)	(3), $*$
χ	Chemotaxis sensitivity coefficient	0-10	Assumed	$*$
D_c	CSF-1 diffusion coefficient	1	1,750 ($\mu\text{m}^2\,\text{seconds}^{-1}$)	$*$
κ_c	CSF-1 production rate	0.03	0.01-0.1 (nM min^{-1})	(22), $*$

*Estimated to maintain realistic model behaviour.

Parameter values. Table S1 contains dimensionless parameter values used in the ABM simulations and ranges of their dimensional counterparts. Parameter values with $*$ are based on estimates which have been chosen in order to produce biologically reasonable behavior, where no suitable reference value can be identified. In particular, parameter values were chosen to ensure that spheroids remained compact, exhibited logistic growth patterns characteristic of

diffusion-limited spheroid growth and, where possible, were consistent with previous modeling studies. Following previous work of (3), (23) and (16), dimensional values for parameters relating to oxygen thresholds are stated in terms of partial pressures, p. These can be converted to concentrations, ω, using Henry's Law $p = \Omega\omega$, with $\Omega = 3.0318 \times 10^7$mmHg kg m^{-3}.

B. Head and Neck Clinical Histology Images.

IHC data collection protocol. As previously reported (24), all patients gave informed consent for use of their tissue in research. Access to the tissue samples analyzed in this study was approved under Oxford Radcliffe Biobank (ORB) research tissue bank ethics, reference 09/H0606/5+5 (approved by the National Research Ethics Service [NRES] Committee South Central – Oxford C). All experimental protocols were approved prospectively by the ORB committee and subsequently conducted in accordance with its conditions and those of NRES. 4 μm sections were cut from formalin-fixed paraffin embedded tissue blocks of 16 cases of head and neck squamous cell carcinoma (HNSCC). These (near) serial sections underwent IHC staining on a Leica BOND-MAX automated staining machine (Leica Biosystems). Briefly, sections were deparaffinized, underwent epitope retrieval and endogenous peroxidase activity was blocked with 3% hydrogen peroxide (5 minutes). Subsequently, sections were incubated with the primary antibody (30 minutes) followed by post-primary and polymer reagents (8 minutes each). Next, 3,3'-Diaminobenzidine (DAB) chromogen was applied (10 minutes) (all reagents contained within the BOND Polymer Refine Detection kit, Leica Biosystems, catalog no. DS9800). The following primary antibodies were used to stain individual sections:

- CD8 – mouse monoclonal clone C8/144B, Agilent Technologies (catalog reference: M710301-2), 1:100 concentration;
- FoxP3 – mouse monoclonal clone 236A/E7, Abcam (catalog reference: ab20034), 1:100 concentration;
- CD68 – mouse monoclonal clone PG-M1, Agilent Technologies (catalog reference: M087601-2), 1:200 concentration;
- Pimonidazole – mouse monoclonal clone 4.3.11.3, Hypoxyprobe Inc. (catalog reference: Mouse-Mab), 1:1000 concentration;
- CAIX – rabbit polyclonal, Abcam (catalog reference: ab15086), 1:2000 concentration;
- Pancytokeratin – rabbit polyclonal, Abcam (catalog reference: ab9377), 1:200 concentration.

Stained slides were scanned at x200 magnification using the NanoZoomer S210 digital slide scanner (Hamamatsu) and co-registered to allow comparison of labeled cells and regions.

Image analysis of clinical data. We use a bespoke image analysis pipeline (25), implemented in MATLAB (MathWorks), to extract CD8$^+$, FoxP3$^+$and CD68$^+$cell locations from IHC slides as (x,y)-coordinates for downstream analysis (Figure S2). Initially, whole slide IHC images were reviewed by a trained pathologist (PSM) who annotated tumor regions, areas of necrosis and any artifactual changes for exclusion from analysis. For the dataset presented in Figure 3, 1.5 mm $\times$ 1.5 mm regions of interest were then selected to saturate as much of the tumor tissue as possible whilst avoiding any artifacts present on the stained slides. Each region was then extracted at 100x effective magnification (resolution of 0.882 μm per pixel) for analysis. For the dataset presented in Figure 4, larger regions of interest ($\approx$ 4.75 mm $\times$ 4 mm) were extracted from the CD8, FoxP3, CD68, pimonidazole, CAIX and pancytokeratin labelled slides, computationally aligned and merged into a single multi-labeled image. Following the process described by Bull *et al.* in (25), we then applied the Simple Linear Iterative Clustering (SLIC) superpixellation algorithm (26) to obtain an oversegmented image in which each cell is represented by multiple superpixels. We collected 26 summary features from each superpixel: the mean and standard deviation of values of the red, green and blue color channels of constituent pixels; the mean and standard deviation of these color channels in neighboring superpixels; the means and standard deviation of these color channels in neighbors of neighboring superpixels, and morphological properties of the superpixel (e.g., height, width, aspect ratio, number of constituent pixels).

We applied a support vector machine (SVM) classifier (27) to identify positively stained superpixels and to obtain a mask of positive pixels. For CD8, FoxP3 and CD68 labeled images, watershed segmentation was used to split the corresponding mask into connected components. Using stain-specific parameters identified by PSM, connected components below a threshold were classed as noise and excluded from the mask. Connected components larger than a second threshold were deemed too large to represent a single cell and were bisected midway along the longest axis. This process was repeated until all connected components had an area within the target range. The centroids of the

connected components were converted into (x, y)-coordinates for analysis. For pimonidazole, CAIX and pancytokeratin images, positively stained regions were identified and combined with manual annotations of regions of necrosis to derive overlay masks that were used to segment tumor cell nests from tumor stroma and to define gradients of oxygen availability. In keeping with previous studies (28), we report 'hypoxic fractions' for each marker, defined as the hypoxia marker positive surface area divided by the total tumor surface area (including both epithelial and stromal components).

Performance of image analysis pipeline. Results validating the performance of our image analysis pipeline can be found in the Supplementary Information of (25). We also include here Figure S3, which reproduces the IHC region associated with Figure 4 in the main text. We show magnifications of three representative regions of the $CD8^+$IHC slide, together with the corresponding magnified regions of the aligned $FoxP3^+$and $CD68^+$slides. Immune cells detected by our pipeline are circled in gold ($CD8^+$), teal ($FoxP3^+$) and purple ($CD68^+$) in the relevant images.

Hypoxia markers. Well-vascularized stromal regions are the best oxygenated areas within tumors, with the perivascular partial pressure of oxygen reported to be approximately 30 mmHg (29). Endogenous (genetic) hypoxia markers, such as carbonic anhydrase 9 (CAIX), are activated within tumor cells via the hypoxia-inducible factor (HIF) system at an oxygen partial pressure of approximately 20 mmHg (30). By comparison, exogenous (chemical) hypoxia markers, such as pimonidazole, label more profoundly hypoxic tumor regions with a partial pressure of oxygen below approximately 10 mmHg (31). Regions of necrosis, identified by manual annotations by a pathologist, are virtually anoxic (32). Figure S4 provides exemplar images of these hypoxia markers in a human head and neck tumor.

2. Analysis Techniques.

A. Spatial Statistics.

Pair-correlation function. The pair-correlation function (PCF), $g(r)$, is a second-order spatial statistic which can identify clustering and dispersion in point datasets.

We calculate the PCF as follows. For each point in the dataset, we calculate the ratio of the number of points observed in an annulus of width dr and inner radius r to the number of points expected to be in the annulus if points were distributed according to complete spatial randomness (CSR). We average these values across all points to determine the PCF, $g(r)$ (see Figure S5). Under CSR, the expected number of points within an annulus of area A is $n = Ad$, where d is the density of points in the domain.

If $g(r) > 1$ then more points are separated by radius r than would be expected under CSR. Similarly, $g(r) < 1$ indicates that fewer points are separated by distance r than expected by CSR. Thus, the PCF describes clustering and dispersal of points over different length scales. We consider the maximum observed value of the PCF, denoted $g_{\max}$. This summary statistic can be interpreted as describing how densely clustered the points are in comparison to CSR at any length scale.

B. Topological Techniques.

Introduction to Persistent Homology. In this section we introduce persistent homology (PH), a technique from the field of Topological Data Analysis which is used to extract topological features of data. PH extracts non-linear features of a dataset and can be applied to a range of data types (33–39). Interactive examples demonstrating single parameter persistent homology (1-PH), multiparameter persistent homology (MPH) and persistence landscapes are available online at (40) and a video tutorial is available at https://drive.google.com/drive/folders/1X20C1RYZyk6cmkcRX9NZ9MGdrE41kRqu?usp=sharing.

PH enjoys a number of desirable properties which make it a viable technique for use with biological datasets. The topological summary produced by PH provides a multiscale description of a dataset. A multiscale descriptor is particularly useful for biological datasets since the length scales over which biological phenomena occur may not be known a priori. By contrast, traditional data analysis techniques such as machine learning and various statistical tests, focus on a single length scale. The extraction of topological features through PH satisfies a stability result (Theorem 2), which guarantees that similar datasets produce similar topological summaries. Biological datasets are susceptible to the introduction of noise in various stages of data collection and processing, as well as the noise derived from the inherent stochasticity of biological processes. The robustness that derives from the stability result for PH means that the topological summaries produced by PH theory are not very sensitive to small perturbations of datasets. However, outliers can disrupt topological features. We demonstrate the sensitivity to moderate levels of outlier noise in our analysis.

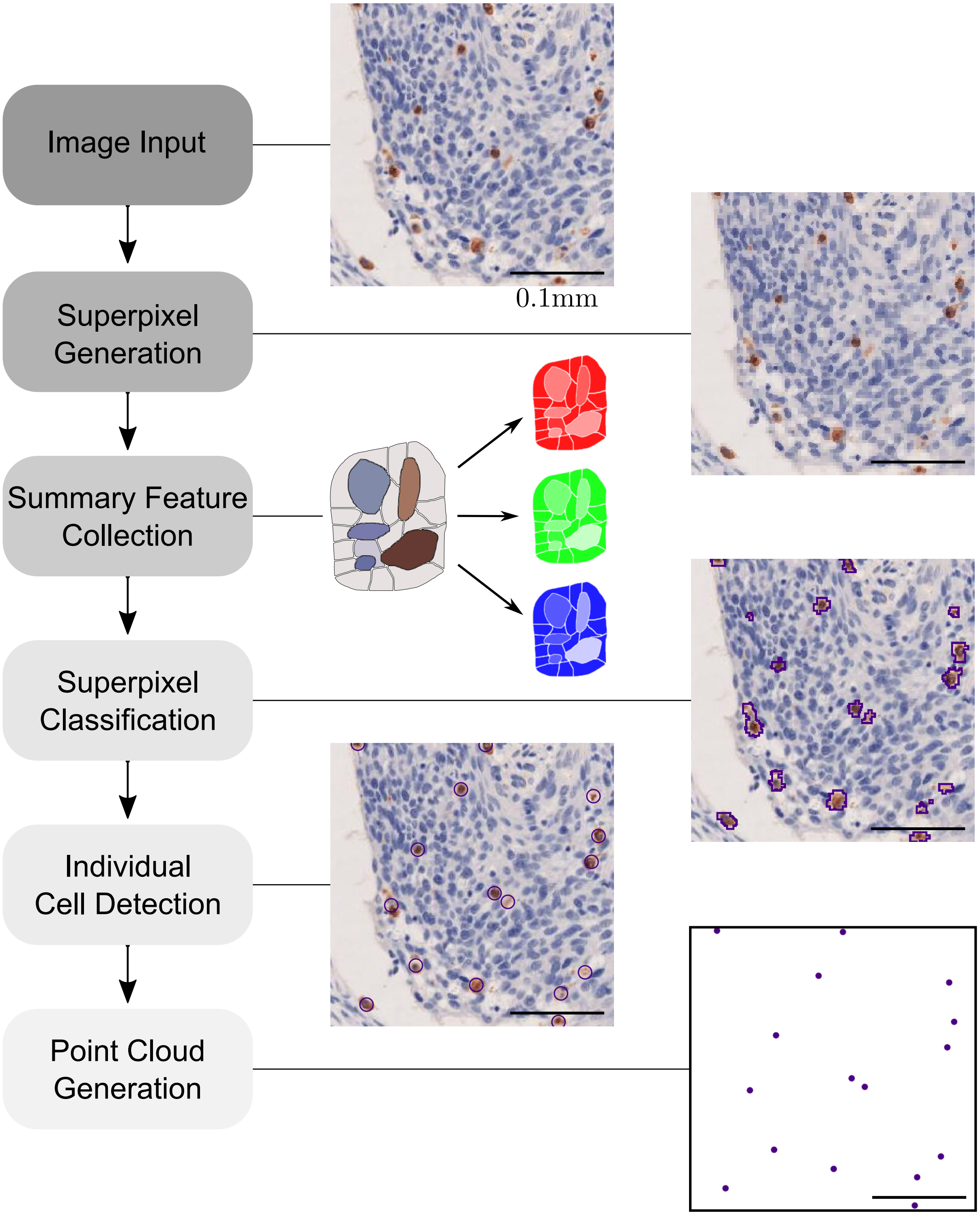

Fig. S2. **Workflow for extracting point clouds from immunohistochemistry (IHC) images**
Workflow used to extract (x, y)-coordinates of cell centroids from IHC slides, demonstrated here on a $300\ \mu m \times 300\ \mu m$ region extracted from a head and neck tumor IHC slide stained to show CD68^{+} macrophages. The input image is converted into superpixels, and the summary features of each superpixel are calculated. These features are used to classify each superpixel using a support vector machine classifier. Individual cells are identified via watershedding.

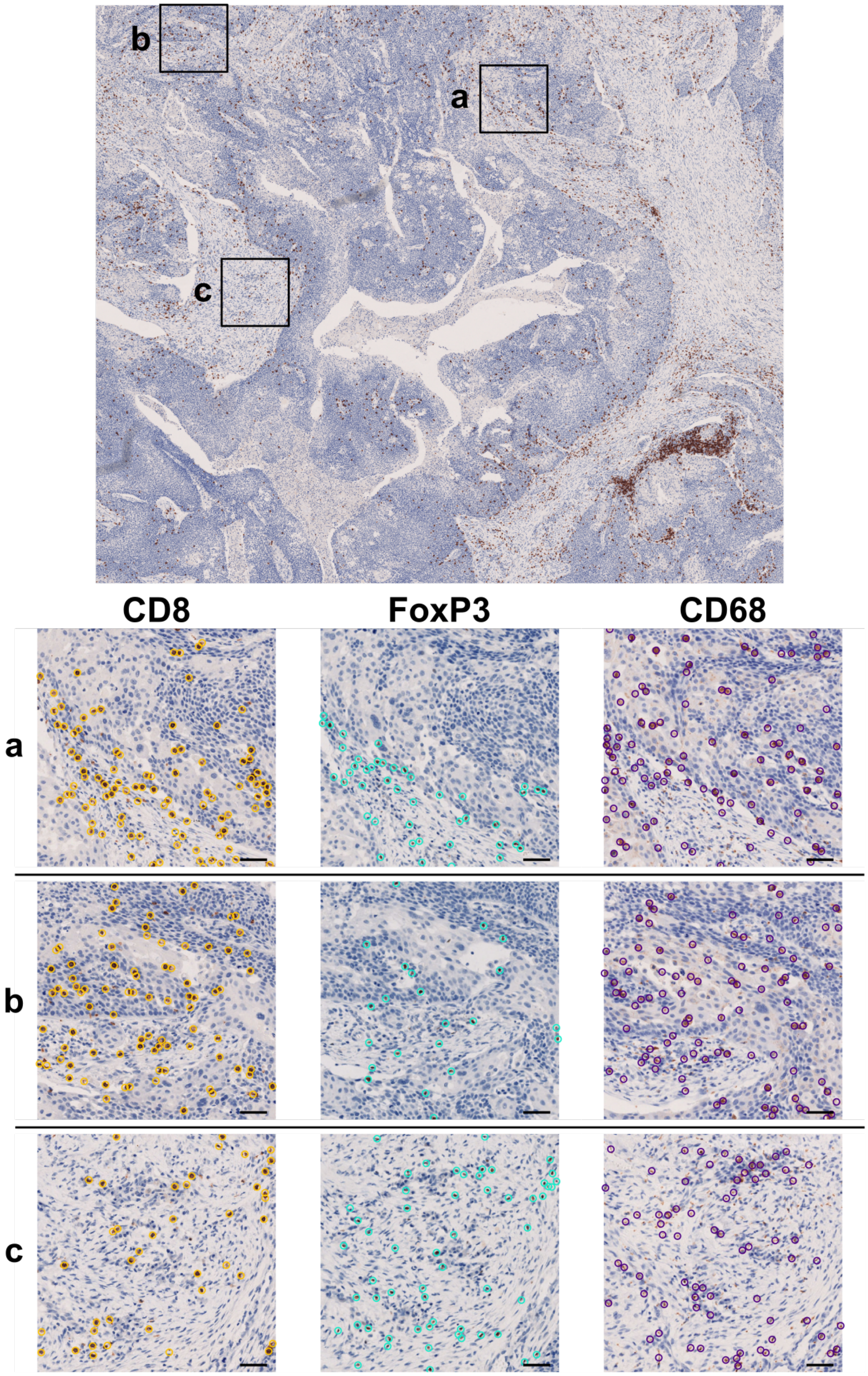

Fig. S3. Examples of cell detection in the region analyzed in Figure 4
Magnified immunohistochemistry (IHC) regions showing points used in the analysis for Figure 4. The magnified regions shown in (a)-(c) come from the $CD8^+$, $FoxP3^+$ and $CD68^+$ IHC slides respectively. Scale bars are 50μm.

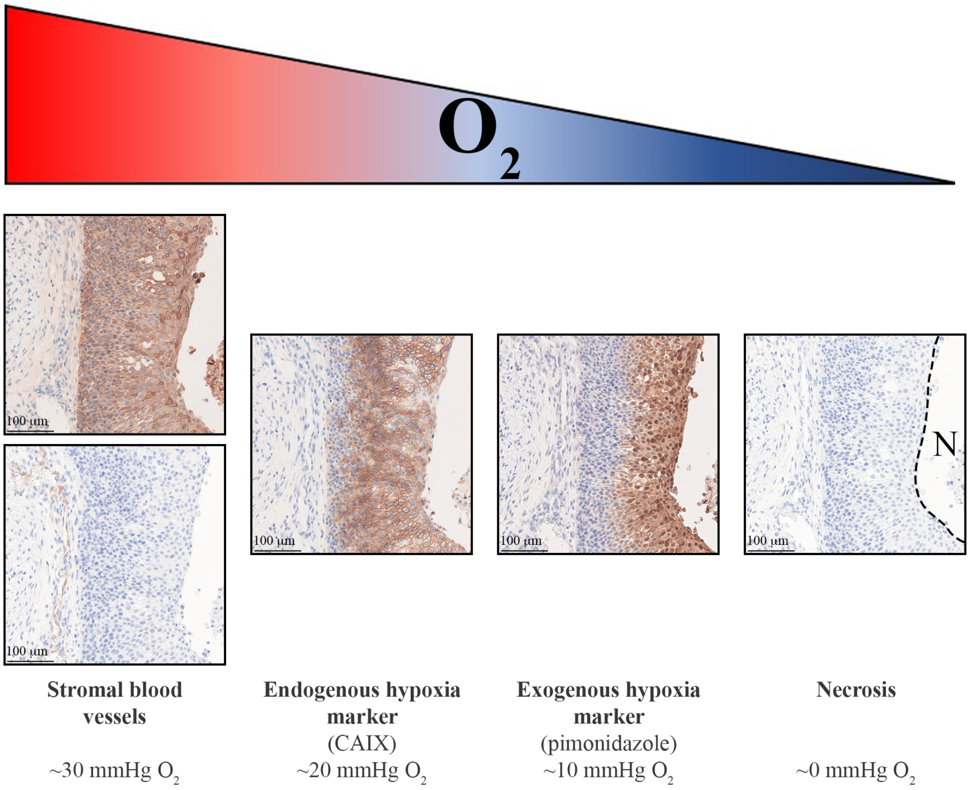

Fig. S4. Mapping oxygen (O_2) availability in solid tumors by immunohistochemistry.
Oxygen gradients exist within solid tumors as a result of complex interaction between factors that include the blood's oxygen carrying capacity, the integrity and function of tumor blood vessels and the metabolic demands of tumor and stromal cells. These gradients can be mapped by combining a panel of different immunohistochemical markers. In this example, the histological images, from left to right, illustrate: (1) differentiation of stroma (pancytokeratin negative) and tumor cell nests (pancytokeratin positive) (upper panel) and labeling of stromal blood vessels with the endothelial marker CD31 (lower panel), (2) tumor cell expression of the endogenous hypoxia marker carbonic anhydrase 9 (CAIX) and (3) the adducts formed by the exogenous hypoxia marker pimonidazole and (4) an area of necrosis (N).

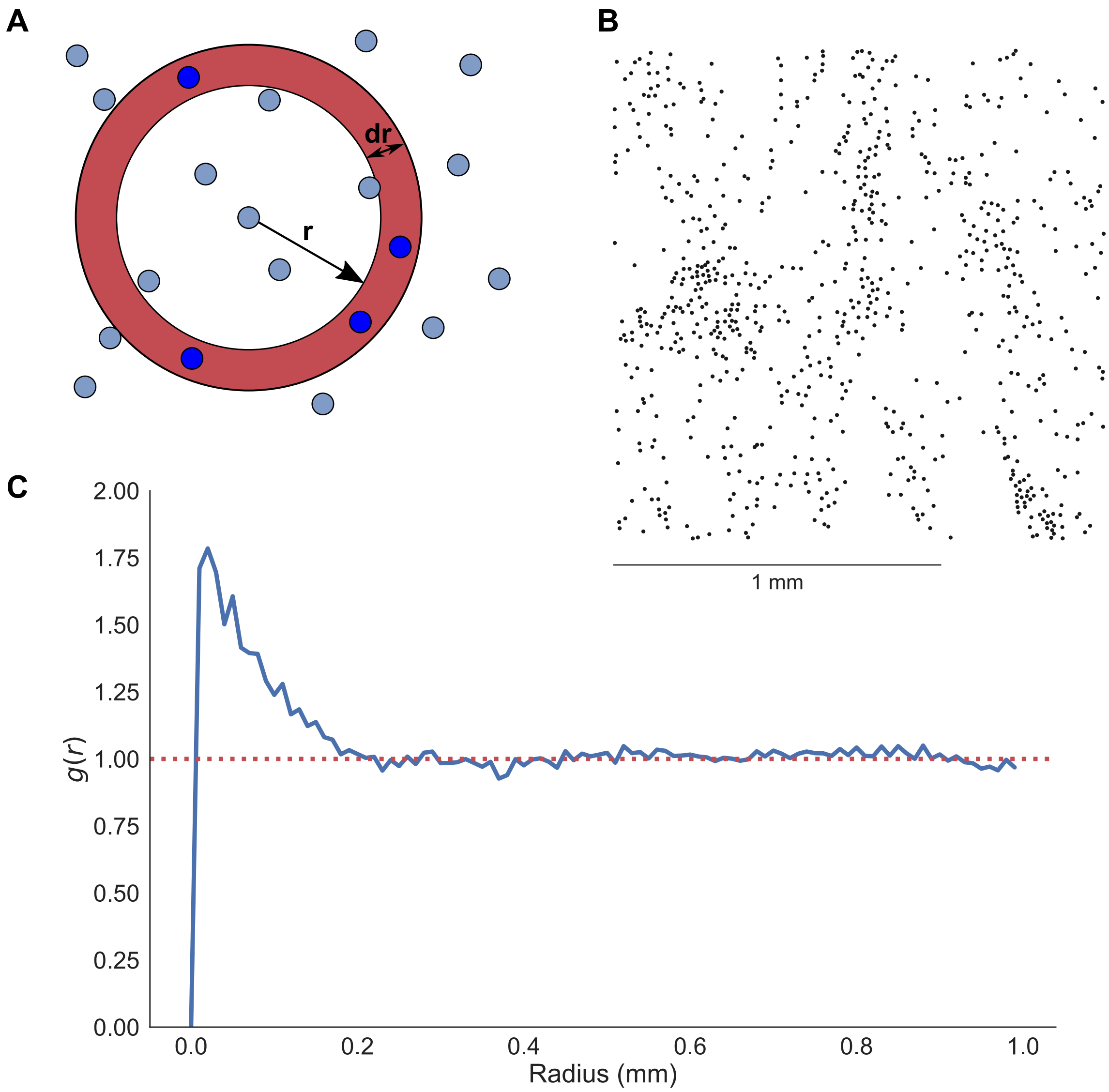

Fig. S5. Example calculation of the pair-correlation function (PCF)
A: Schematic showing how the the pair-correlation function (PCF), $g(r)$, is calculated. An annulus of width dr and inner radius r is centered at each point in turn, and the number of points observed within the annulus is recorded. This number is then averaged over all points in the point cloud, and compared with the number of points expected to lie within the annulus under complete spatial randomness. B: Points derived from macrophage ($CD68^+$) locations in a $1.5\,\mathrm{mm} \times 1.5\,\mathrm{mm}$ immunohistochemistry image of a sample of human head and neck cancer. C: PCF calculated for the point cloud in B. When the radius is less than approximately 20 μm, $g(r) < 1$, suggesting dispersion of points; this length scale corresponds to the approximate size of a macrophage and indicates that identified macrophage centers are at least one cell diameter apart. The PCF, $g(r)$, peaks at $r \approx 0.06$mm, indicating that macrophages are approximately 1.75 times more likely to be found within 0.06mm of another macrophage than if they were randomly distributed in the domain.

Single Parameter Persistent Homology (1-PH). In this section we introduce the basic concepts from 1-PH required to describe our analysis techniques. See (41) and (42) for a more detailed exposition of 1-PH.

Definition 1 *(1-Parameter Filtration) Let X be a topological space and $\{X_t\}_{t\in\mathbb{R}}$ a collection of subspaces of X such that $X_s \subset X_t$ for all $s \leq t$. We say that $\{X_t\}_{t\in\mathbb{R}}$ is a 1-parameter filtration of the topological space X if $X = \bigcup_{t\in\mathbb{R}} X_t$.*

Definition 2 *(Single Parameter Persistence Module) Let $\{V_t\}_{t\in\mathbb{R}}$ be a collection of vector spaces and $\{\iota_{s,t} : V_s \to V_t\}_{s\leq t}$ a collection of linear maps such that $\iota_{t,t} = id_{V_t}$ and $\iota_{s,t} \circ \iota_{r,s} = \iota_{r,t}$. We say that the collection of this data is a single parameter persistence module which we shall simply denote by V. This data can be thought of as an $\mathbb{R}$-graded module over the monoid ring $([0,\infty), +)$ with the action given by the linear maps, that is to say for all $v \in V_t$ and $a \in [0,\infty)$ we have $a \cdot v = \iota_{t,t+a}(v)$.*

Example 1 *(Sublevel Set Single Parameter Persistent Homology) Let X be a topological space equipped with a filtering function $f : X \to \mathbb{R}$, inducing a $\mathbb{R}$-indexed collection of sublevel sets $\{X_t = f^{-1}((-\infty, t])\}_{t\in\mathbb{R}}$ and a collection of inclusion maps $\{i_{s,t} : X_s \to X_t\}_{s\leq t}$. Let H denote a homology functor with coefficients in a field. Applying the homology functor H to the collection of sublevel sets and inclusion maps gives rise to a single parameter persistence module, $\{V_t = H(X_t)\}_{t\in\mathbb{R}}$, $\{\iota_{s,t} = H(i_{s,t}) : V_s \to V_t\}_{s\leq t}$, called persistent homology (1-PH).*

The choice of filtering function is crucial in the formation of a persistence module. A filtering function can be tailored to the specific application one has in mind for a dataset. The filtering function may be chosen to track the spatial distribution of data.

Example 2 *(Čech Filtration) Let (M, d) be a metric space and $P \subset M$ a collection of points in the metric space. Consider the filtering function $dist_P : M \to \mathbb{R}$, defined to be the distance function from the collection of points: $dist_P(x) = \min_{p\in P} d(x, p)$. The induced $\mathbb{R}_{\geq 0}$-indexed collection of sublevel sets $\{X_r = {dist_P}^{-1}((-\infty, r])\}_{r\in\mathbb{R}_{\geq 0}}$ and collection of inclusion maps $\{i_{r,s} : X_r \to X_s\}_{r\leq s}$ is known as the Čech filtration.*

The Čech filtration is commonly used in applications of persistent homology. For well-behaved metric spaces such as Euclidean space, one can encode the topology of the filtration in a combinatorial object called a filtered simplicial complex. Moreover, the Čech filtration may be approximated by a simpler filtration known as the Vietoris-Rips filtration. The Vietoris-Rips filtration is more efficiently computable and will be used in our computations. An example Čech filtration for a point cloud is illustrated in Figure S8c.

Theorem 1 *(Decomposition Theorem)(43) Let $I \subset \mathbb{R}$ be an interval and let V^I denote the single parameter persistence module such that $\dim V^I_t = \mathbf{1}_I(t)$ and such that the linear maps $\iota_{s,t}$ are isomorphisms for all $s, t \in I$. If V is a single parameter persistence module such that $\dim V_t < \infty$ for all $t \in \mathbb{R}$ then V admits a unique decomposition $V \cong \bigoplus_{I\in\mathcal{B}} V^I$ for some multiset of intervals $\mathcal{B}$. The multiset $\mathcal{B}$ is known as the barcode of the persistence module V.*

Definition 3 *(Interleaving Distance) Let V and W be single parameter persistence modules. An ϵ-interleaving between modules V and W is specified by a collection of linear maps $\{\phi_t : V_t \to W_{t+\epsilon}\}_{t\in\mathbb{R}}$,$\{\psi_t : W_t \to V_{t+\epsilon}\}_{t\in\mathbb{R}}$ such that for all $t \in \mathbb{R}$ these linear maps satisfy $\psi_{t+\epsilon} \circ \phi_t = \iota^V_{t,t+2\epsilon}$ and $\phi_{t+\epsilon} \circ \psi_t = \iota^W_{t,t+2\epsilon}$. If an ϵ interleaving between V and W exists we say that V and W are ϵ-interleaved. The interleaving distance between a pair of modules is denoted by d_I and given by:*

$$d_I(V, W) = \inf\{\epsilon \geq 0 : V \text{ and } W \text{ are } \epsilon - \text{interleaved}\}$$

where we take the infimum of the empty set to be ∞.

The interleaving distance is an extended pseudo-metric on the collection of persistence modules and so satisfies the intuitive properties one would want from a distance function to compare these algebraic objects. For well-behaved persistence modules that arise in data analysis the interleaving distance is computable.

Theorem 2 *(Stability Theorem) Let X be a topological space and $f, g : X \to \mathbb{R}$ be a pair of filtering functions, and $V(f), V(g)$ be the associated sublevel set single parameter persistence modules. Then*

$$d_I(V(f), V(g)) \leq \|f - g\|_\infty$$

Consider the case that f and g are the distance functions associated to point clouds P and P' in a metric space, that is $f = \mathrm{dist}_P$ and $g = \mathrm{dist}_{P'}$. It is straightforward to show that the infinity norm between f and g is the Hausdorff distance between the point clouds P and P'. If P' is a perturbation of the collection of points P then the Hausdorff distance between the two point clouds will be small, and, thus, by Theorem 2, the resulting sublevel set single parameter persistence modules will be close in the interleaving distance. However, if one of the points in P' is distant from all points in P, the Hausdorff distance between the point clouds is large and there is no guarantee that the resulting modules will be close in the interleaving distance. In this sense, single parameter persistent homology is stable to perturbations of point clouds but sensitive to the introduction of outliers.

Multiparameter Persistent Homology. Multiparameter persistent homology is a topic of considerable research interest for the Topological Data Analysis community (44–46). The algebraic objects which arise in the study of multiparameter persistence are significantly more complex than their single parameter counterparts. Multiparameter persistence facilitates the study of richer topological properties of data inaccessible by single parameter persistence. In return for richer topological summaries, one has to pay the price of increased complexity in the computation of multiparameter persistence (47). As a result, multiparameter persistence has largely remained a topic of theoretical interest and has not been applied as a data analysis technique as widely as single parameter persistence. The techniques used in this work provide a framework for the application of multiparameter persistence, applicable to a wide variety of datasets. In this section we shall outline the theory of multiparameter persistence required to exposit our novel multiparameter persistence techniques.

Throughout this section we shall consider $\mathbb{R}^n$ equipped with the following partial order: $(s_1, ..., s_n) = \mathbf{s} \leq \mathbf{t} = (t_1, ..., t_n)$ if and only if $s_i \leq t_i$ for all $i = 1, ..., n$.

Definition 4 *(Multiparameter Filtration) Let X be a topological space and $\{X_\mathbf{t}\}_{\mathbf{t}\in\mathbb{R}^n}$ a collection of subspaces of X such that $X_\mathbf{s} \subset X_\mathbf{t}$ for all $\mathbf{s} \leq \mathbf{t}$. We say that $\{X_\mathbf{t}\}_{\mathbf{t}\in\mathbb{R}^n}$ is a multiparameter filtration of the topological space X if $X = \bigcup_{\mathbf{t}\in\mathbb{R}^n} X_\mathbf{t}$.*

Definition 5 *(Multiparameter Persistence Module) Let $\{V_\mathbf{t}\}_{\mathbf{t}\in\mathbb{R}^n}$ be a collection of vector spaces and $\{\iota_{\mathbf{s},\mathbf{t}} : V_s \to V_t\}_{\mathbf{s}\leq\mathbf{t}}$ a collection of linear maps such that $\iota_{\mathbf{t},\mathbf{t}} = id_{V_\mathbf{t}}$ and $\iota_{\mathbf{s},\mathbf{t}} \circ \iota_{\mathbf{r},\mathbf{s}} = \iota_{\mathbf{r},\mathbf{t}}$. We say that the collection of this data is a multiparameter persistence module which we shall simply denote by V. This data can be thought of as an $\mathbb{R}^n$-graded module over the monoid ring $([0,\infty)^n, +)$ with the action given by the linear maps, that is to say for all $v \in V_\mathbf{t}$ and $\mathbf{a} \in [0,\infty)^n$ we have $\mathbf{a} \cdot v = \iota_{\mathbf{t},\mathbf{t}+\mathbf{a}}(v)$.*

Example 3 *(Sublevel Set Multiparameter Persistent Homology) Let X be a topological space equipped with a filtering function $f : X \to \mathbb{R}^n$, inducing an $\mathbb{R}^n$-indexed collection of sublevel sets $\{X_\mathbf{t} = f^{-1}(\{\leq \mathbf{t}\})\}_{\mathbf{t}\in\mathbb{R}^n}$ and a collection of inclusion maps $\{i_{\mathbf{s},\mathbf{t}} : X_\mathbf{s} \to X_\mathbf{t}\}_{\mathbf{s}\leq\mathbf{t}}$. Let H denote a homology functor with coefficients in a field. Applying the homology functor H to the collection of sublevel sets and inclusion maps gives rise to a multiparameter persistence module, $\{V_\mathbf{t} = H(X_\mathbf{t})\}_{\mathbf{t}\in\mathbb{R}^n}$, $\{\iota_{\mathbf{s},\mathbf{t}} = H(i_{\mathbf{s},\mathbf{t}}) : V_\mathbf{s} \to V_\mathbf{t}\}_{\mathbf{s}\leq\mathbf{t}}$, called multiparameter persistent homology (MPH).*

Multiparameter persistence modules enable a richer choice of filtering function than single parameter persistence. The filtering function may be chosen to track both the spatial distribution of data together with its interdependence with other parameters of interest (48). The other parameters of interest can include further spatial parameters such as density or eccentricity, as well as parameters independent of the spatial distribution such as charge or oxygen concentration or some other chemical marker.

Example 4 *(Čech-Codensity Filtration) Let (M, d) be a metric space and $P \subset M$ a collection of points in the metric space. Let $dist_Q : M \to \mathbb{R}$, denote the distance function from the collection of points Q: $dist_Q(x) = \min_{q\in Q} d(x,q)$ and let $f : P \to \mathbb{R}$ be a codensity function. Let $P_\rho = f^{-1}((-\infty, \rho])$. Consider the multiparameter filtration $\{X_{(r,\rho)} = {dist_{P_\rho}}^{-1}((-\infty, r])\}$. We call this filtration the Čech-Codensity filtration.*

We illustrate an example Čech-Codensity filtration in Figure S11b. As in the single parameter case, we will approximate the Čech-Codensity filtration using a computationally cheaper filtration which computes the Vietoris-Rips complex rather than the Čech complex, which we shall refer to as the radius-codensity filtration.

Definition 6 *(Interleaving Distance) Let V and W be multiparameter persistence modules. Let ϵ denote the diagonal vector $(\epsilon, ..., \epsilon)$. An ϵ-interleaving between modules V and W is specified by a collection of linear maps $\{\phi_\mathbf{t} : V_\mathbf{t} \to W_{\mathbf{t}+\epsilon}\}_{\mathbf{t}\in\mathbb{R}^n}$,$\{\psi_\mathbf{t} : W_\mathbf{t} \to V_{\mathbf{t}+\epsilon}\}_{\mathbf{t}\in\mathbb{R}^n}$ such that for all $t \in \mathbb{R}^n$ these linear maps satisfy $\psi_{\mathbf{t}+\epsilon} \circ \phi_\mathbf{t} = \iota^V_{\mathbf{t},t+2\epsilon}$ and $\phi_{\mathbf{t}+\epsilon} \circ \psi_\mathbf{t} = \iota^W_{\mathbf{t},t+2\epsilon}$. If an ϵ interleaving between V and W exists we say that V and W are ϵ-interleaved. The interleaving distance between a pair of modules is denoted by d_I and given by:*

$$d_I(V, W) = \inf\{\epsilon \geq 0 : V \text{ and } W \text{ are } \epsilon - \text{interleaved}\}$$

where we take the infimum of the empty set to be ∞.

The interleaving distance is an extended pseudo-metric on the collection of multiparameter persistence modules (49) and so satisfies the intuitive properties one would want from a sensible distance function to compare multiparameter persistence modules. However, in distinct contrast to the single parameter setting, the interleaving distance is NP-hard to compute and approximate for multiparameter persistence modules (47). The difficulty in computing the interleaving distance in the multiparameter setting points towards the increased complexity of multiparameter persistence modules. Nevertheless the interleaving distance for multiparameter modules still satisfies the stability theorem.

Theorem 3 *(Stability Theorem) Let X be a topological space and $f, g : X \to \mathbb{R}^n$ be a pair of filtering functions, and $V(f), V(g)$ be the associated sublevel set multiparameter persistence modules:*

$$d_I(V(f), V(g)) \leq \|f - g\|_\infty$$

Persistence Landscapes and Statistics. In this section we shall introduce a vectorization technique for single parameter and multiparameter persistence modules. A principal advantage of vectorizing persistence modules is that one can leverage traditional data analysis techniques and statistical techniques on the resulting topological feature vectors. There are a wide range of vectorization techniques for single parameter persistence (50–54). Recent work has seen the development of a couple of vectorization techniques for multiparameter persistence (48, 55, 56). We choose to use the multiparameter persistence landscapes (48) due to the computational feasibility and interpretability of this vectorization technique, as well as the readily available statistical tools.

Persistence landscapes were first introduced in (51) and have subsequently been widely used as a vectorization technique for single parameter persistent homology (57–59). The article (48) extends the persistence landscape to the multiparameter setting of $\mathbb{R}^n$ indexed modules. This extension coincides with the single parameter persistence landscape in the case $n = 1$. We recall the definition of the multiparameter persistence landscape and some of the statistical properties enjoyed by the persistence landscape. Further properties of the single parameter persistence landscape are explored in (60–62) and further properties of the multiparameter persistence landscape are explored in (48).

Definition 7 *(Multiparameter Persistence Landscape) Let V be a multiparameter persistence module, the associated multiparameter persistence module is a function $\lambda : \mathbb{N} \times \mathbb{R}^n \to \mathbb{R}_{\geq 0}$ given by:*

$$\lambda(k, \mathbf{x}) = \sup\{\epsilon \geq 0 : \mathit{rank}(\iota_{\mathbf{x}-\epsilon\mathbf{1},\mathbf{x}+\epsilon\mathbf{1}}) \geq k\}$$

where the supremum of the empty set is taken to be 0.

The multiparameter persistence landscape is computable, interpretable and amenable to statistical analysis. The multiparameter persistence landscape associated to a multiparameter persistence module lies in a Banach space $L^p(\mathbb{N} \times \mathbb{R}^n)$. We shall refer to the p-norm distance between a pair of landscapes associated to persistence modules as the p-landscape distance between these modules. This distance is readily computed and is stable with respect to the interleaving distance.

Theorem 4 *(Multiparameter Persistence Landscape Stability) Let V, V' be multiparameter persistence modules with associated landscapes λ, λ'. Let $E \subset \mathbb{N} \times \mathbb{R}^n$ be a Lebesgue measurable subset with measure $|E|$ and characteristic function χ_E. The multiparameter persistence landscapes satisfy the following stability property:*

$$\|\lambda - \lambda'\|_\infty \leq d_I(V, V')$$

$$\|(\lambda - \lambda')\chi_E\|_p \leq |E| d_I(V, V') \textit{ for all } p \in [1, \infty).$$

We can view the multiparameter persistence landscape associated to a dataset as a Banach space valued random variable. Suppose X is a Borel measurable random variable on some probability space $(\Omega, \mathcal{F}, \mathbb{P})$ thought of as sampling data from some distribution. Further let $\Lambda = \Lambda(X)$ denote the multiparameter persistence landscape associated to some filtration of the data X, so that in summary $\Lambda : (\Omega, \mathcal{F}, \mathbb{P}) \to L^p(\mathbb{N} \times \mathbb{R}^n)$ for $1 \leq p < \infty$ is a random variable taking values in a real, separable Banach Space. Let $\{X_i\}$ be i.i.d copies of X and $\{\Lambda_i\}$ their associated landscapes. Denoting the pointwise mean of the first n landscapes by $\overline{\Lambda}^n$ and applying the general theory of probability in Banach spaces we attain several results. Associated to a well-behaved Banach space valued random variable $\Lambda : (\Omega, \mathcal{F}, \mathbb{P}) \to L^p(\mathbb{N} \times \mathbb{R}^n)$ is a set function $I_\Lambda : \mathcal{F} \to L^p(\mathbb{N} \times \mathbb{R}^n)$ called the Pettis Integral of Λ. This can be thought of as the expectation of a Banach space valued random variable. For more details see

Theorem 5 *(Strong Law of Large Numbers) With our notation as in the above discussion $\overline{\Lambda}^n \to I_\Lambda(\Omega)$ almost surely if and only if $\mathbb{E}[\|\Lambda\|] < \infty$.*

Theorem 6 *(Central Limit Theorem) Let us consider the landscapes endowed with the p-landscape distance for $p \geq 2$. If $\mathbb{E}[\|\Lambda\|] < \infty$ and $\mathbb{E}[\|\Lambda^2\|] < \infty$, then $\sqrt{n}(\overline{\Lambda}^n - I_\Lambda(\Omega))$ converges weakly to a Gaussian random variable $G(\Lambda)$ with the same covariance structure as Λ.*

The central limit theorem for multiparameter persistence landscapes induces a central limit theorem for associated real valued random variables and facilitates the computation of approximate confidence intervals.

Corollary 1 *Let us consider the landscapes endowed with the p-landscape distance for $p \geq 2$. Suppose $\mathbb{E}[\|\Lambda\|] < \infty$ and $\mathbb{E}[\|\Lambda^2\|] < \infty$. If $f \in L^p(\mathbb{N} \times \mathbb{R}^n)^*$, so that $Y = f(\Lambda)$ is a real valued random variable, then $\sqrt{n}(\overline{Y}^n - \mathbb{E}[Y]) \to \mathcal{N}(0, Var(Y))$ converges in distribution.*

Corollary 2 *(Approximate Confidence Intervals) Suppose Y is a real-valued random variable attained from a functional applied to the multiparameter landscape Λ satisfying the conditions of Corollary 1. Let $\{Y_i\}_{i=1}^n$ be i.i.d. instances of this random variable and $S_n^2 = \frac{1}{n-1}\sum_{i=1}^n (Y_i - \overline{Y}_n)^2$ the sample variance. An approximate $(1-\alpha)$ confidence interval for $\mathbb{E}[Y]$ is given by: $[\overline{Y}_n - z_{\frac{\alpha}{2}}\frac{S_n}{\sqrt{n}}, \overline{Y}_n + z_{\frac{\alpha}{2}}\frac{S_n}{\sqrt{n}}]$, where $z_{\frac{\alpha}{2}}$ is the $\frac{\alpha}{2}$ critical value for the normal distribution.*

In practice, a functional of interest could be given by integrating the landscapes over a subset R of the parameter domain, $f_R(\Lambda) = \int_R \Lambda \, d\mu$. These functionals can be used to establish the significance of homological features in different regions of the parameter space.

3. Data Analysis.

A. Multiparameter Persistence Examples. In this section we demonstrate, with a couple of examples, the topological features which may be extracted from a dataset using multiparameter persistence. In particular, we look at radius-codensity filtrations and their associated multiparameter persistence modules. Further examples may be found online (40).

First, we show that we can detect clustering in noisy samples using H_0-modules and their associated multiparameter persistence landscapes. We produce two groups of point clouds: a group we call *One Cluster* consisting of 200 points uniformly sampled from a unit disc (see Figure S6a), and a group we call *Two Clusters* consisting of 200 points uniformly sampled from a unit disc together with two dense clusters of 30 points centered at coordinates $(0, \frac{1}{2}), (0, -\frac{1}{2})$ within the unit disc (see Figure S6b).

We plot the average persistence landscapes $\lambda(k, \mathbf{x})$ in the parameter range $[0,1]^2$ for $k \in \{1, ..., 5\}$ for the H_0-modules of the radius-codensity filtrations of each group (see Figures S6c and S6d). Recall that the kth landscape detects the parameter values for which the associated space has at least k-homological features together with the persistence of those features. In this case, the H_0-functor detects connected components. Thus the persistence landscape $\lambda(k, \mathbf{x})$ is non-zero at the parameter value $\mathbf{x}$ if the space $X_{\mathbf{x}}$ has at least k-connected components, and the height of the landscape at parameter value $\mathbf{x}$ corresponds to the persistence of these components. The first landscapes $\lambda(1, \mathbf{x})$ are thus identical for both groups of point clouds, since the radius-codensity filtration of such point clouds support 1 connected component for all parameter values. The second landscapes of the two groups differ since the point clouds with two clusters support 2 connected components across a wide range of parameter values, whereas the one cluster point clouds do not. The remaining landscapes have support only for small Rips filtration parameters, indicating that both groups of point clouds support many connected components when the Rips parameter is small.

In Figure S6e we plot the distributions of the 1-norms of the H_0-landscapes for the two groups of point clouds. We observe that there is a significant difference in the 1-norms of the second H_0-landscapes ($k = 2$). The second H_0-landscapes from the Two Clusters group have larger norm than those from the One Cluster groups and the large drop off in norm between the second and third landscapes indicates that there are two distinct clusters present in the Two Clusters group.

The presence of background noise in the Two Cluster point clouds would render single linkage clustering methods (such as the Rips-filtration for single parameter persistence) unable to detect the two clusters in these point clouds. One could identify that there are two clusters in these samples if we only considered the points from the point cloud in areas of high density. This however, requires us to identify an appropriate density hyperparameter to threshold our point cloud. A significant advantage of our multiparameter approach is that we are able to detect two clusters in this example and, moreover, we are not required to choose a density hyperparameter. This feature of our technique is

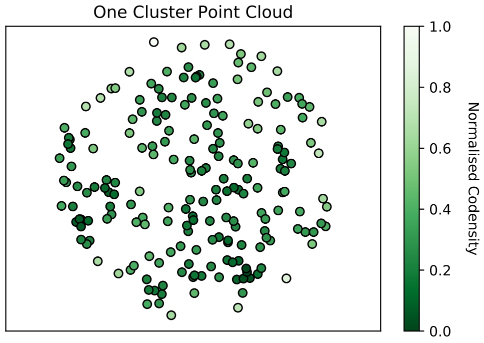

(a) An Example One Cluster Point Cloud.

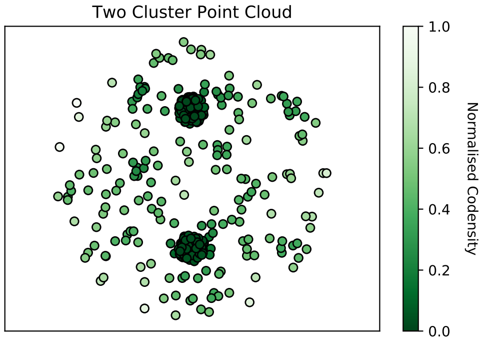

(b) An Example Two Cluster Point Cloud.

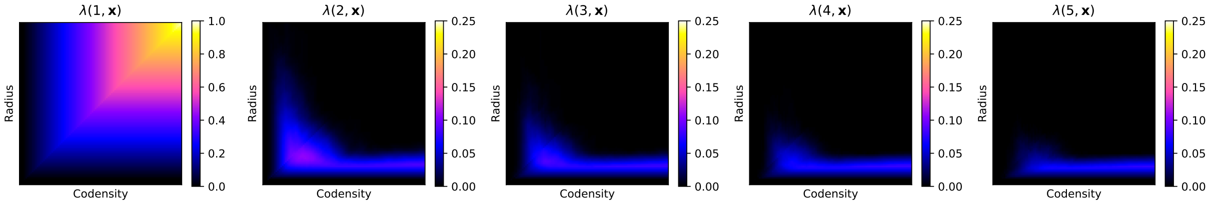

(c) Average Persistence Landscapes for One Cluster Point Clouds (H_0).

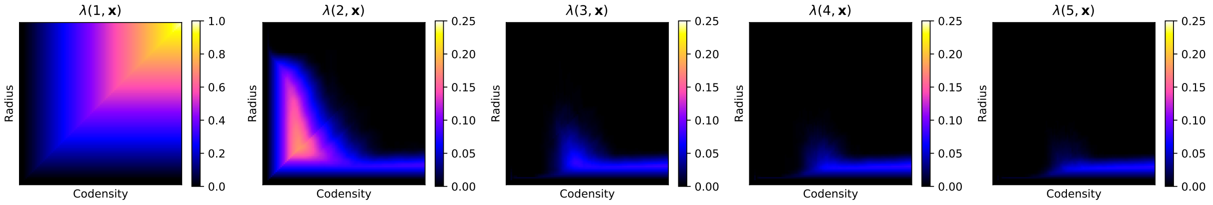

(d) Average Persistence Landscapes for Two Cluster Point Clouds (H_0).

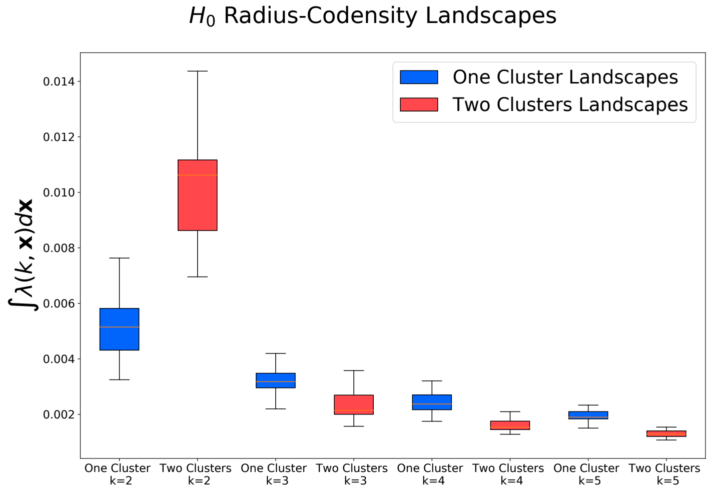

(e) Boxplots comparing the distribution of the multiparameter H_0-landscape 1-norms across the 30 samples.

Fig. S6. MPH-landscapes of data sampled from one or two cluster point clouds.

particularly useful for heterogeneous data where it may not be clear how to identify an appropriate hyperparameter and the appropriate hyperparameter value may differ between samples.

Our second set of examples demonstrates the topological features which may be extracted using H_1-modules and their associated multiparameter persistence landscapes. Figure S7a displays an example point cloud sampled from two discs of different radius and colored by the codensity value of the points. More precisely, the large and small rings have radii $1, \frac{1}{2}$ and centers $(-1, 0), (0.5, 0)$ respectively and 80 points are sampled uniformly from the rings with 20 points uniformly sampled from the discs they enclose. The codensity value for point p is given by $\rho_5(p) = \frac{1}{5}\sum_{i=1}^{5} \|p - p_{(i)}\|_2$ where $p_{(i)}$ is the ith nearest neighbor of p. The point clouds are standardized to have unit variance and the codensity parameter is linearly rescaled so that 95% of values lie in the range $[0, 1]$ and 2.5% lie above and below this range.

Figure S7c displays the first three average persistence landscapes associated to this point cloud distribution. We detect the two rings in the point cloud in the first landscape $\lambda(1, \mathbf{x})$. The smaller ring produces a peak in the first landscape for small codensity and Rips parameter values, whilst the larger ring induces a peak in the first landscape for larger codensity and Rips parameter values. The second landscape does not contain a significant peak, indicating that the range of parameter values for which both rings are detected simultaneously is small.

Figure S7b displays an example point cloud sampled from three discs of the same radius and colored by the codensity value of the points. More precisely, the rings have radius $\frac{1}{2}$ and centers $(1, 0), (0, 0), (\frac{1}{2}, \frac{\sqrt{3}}{2})$. As before, 80 points are sampled uniformly from each of the rings and 20 points uniformly sampled from the discs they enclose. We use the ρ_5 codensity function as before, and once again normalize the point cloud and codensity parameter. Figure S7d displays the first four average persistence landscapes associated to this point cloud distribution. The third landscape peaks in the range of parameter values for which all three rings are detected. The three rings are detected simultaneously since the rings are of the same scale and density of sampling. The fourth landscape contains no significant peak.

This pair of examples demonstrates how one can interpret the persistence landscape for radius-codensity filtrations. We can deduce information about the scale, density and number of loops within a point cloud from the multiparameter persistence landscapes. Multiparameter persistence is able to quantify the structure of these point clouds in ways that traditional spatial statistics cannot (examples of traditional spatial statistics applied to the point patterns in Figure S7 can be seen at https://github.com/JABull1066/SyntheticDataSpatialStats).

B. Single Parameter Persistence for Simulation Data. In this section we apply persistent homology to simulated histology data. This example demonstrates the viability of applying persistent homology techniques to histology data and the types of insight that it affords. The simulated data are generated from the ABM described in Section A.

We consider 5 simulations, with chemotaxis parameter values $\chi = \{0, 2.5, 5, 7.5, 10\}$. Each simulation consists of 25 snapshots. At the start of the simulation, macrophages are introduced to the boundary of a disc of tumor cells (spheroid). Over time, the macrophages are attracted to chemoattractants released by tumor cells under low oxygen (or hypoxia). Thus, initially the macrophages form an annulus and then migrate into the core of the spheroid as the simulation proceeds. Snapshots from a typical simulation are presented in Figure S8a.

At each snapshot we compute the H_1 persistence module for the Vietoris-Rips filtration built upon the point cloud of macrophages (thought of as lying in the metric space $\mathbb{R}^2$). We then compute the ∞-norm of the resulting barcode and trace how this norm evolves throughout the simulation. The larger the norm of the barcode, the larger the inner radius of the annulus formed by the macrophages. Hence, we can track the rate at which the macrophages migrate into the core of the spheroid by tracking the decay of the norm of the barcode.

We plot *decay curves* for each value of the chemotaxis parameter χ in Figure S8d, each curve obtained by averaging over 5 simulations. Several qualitative observations about the behavior of the macrophages can be drawn from the decay curves. First, the value of the chemotaxis parameter χ affects the time at which the macrophages begin to penetrate the spheroid boundary: the larger the chemotaxis parameter, the sooner the macrophages begin to enter into the spheroid. Secondly, the gradient of the linear portion of the decay curves are approximately identical. This indicates that once the macrophages have traversed the spheroid boundary, the rate at which they proceed to its core is independent of the value of the chemotaxis parameter. These observations were attained solely using topological techniques and are consistent with the independent observations of (63).

This simple example application of single parameter persistent homology demonstrates the utility of applying a topological approach to histology-like data. Analyzing *real world* data, rather than simulated data, requires more sophisticated techniques. Indeed, in a *real world* setting data may be corrupted by multiple, unknown, sources of noise. We note that the Čech filtration and the Vietoris-Rips filtration are sensitive to even a single outlier. We use multiparameter persistence to produce topological summaries which are robust to such outliers.

To demonstrate the sensitivity of single parameter persistence to outliers, we rerun the single parameter topological analysis on the simulated ABM data where we artificially introduce measurement error by incorrectly registering 1%

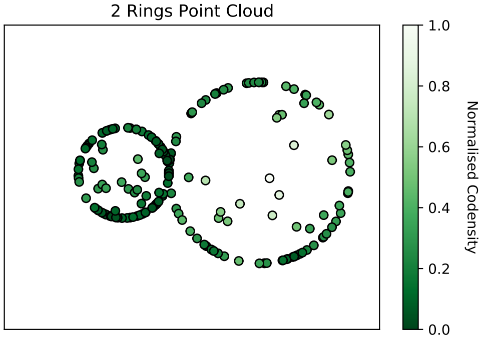

(a) An Example 2 Rings Point Cloud.

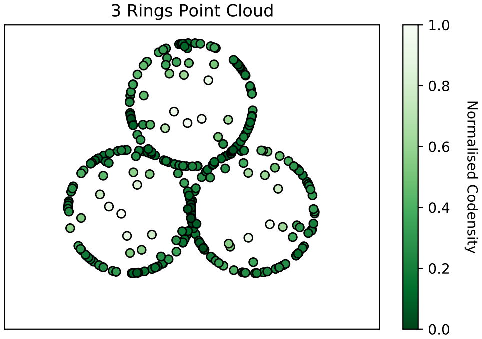

(b) An Example 3 Rings Point Cloud.

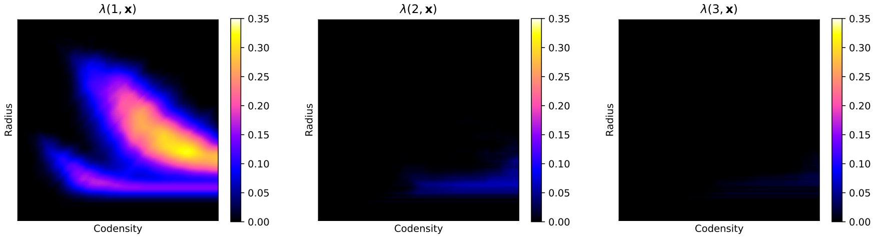

(c) Average Persistence Landscapes for 2 Rings Point Clouds taken over 30 samples.

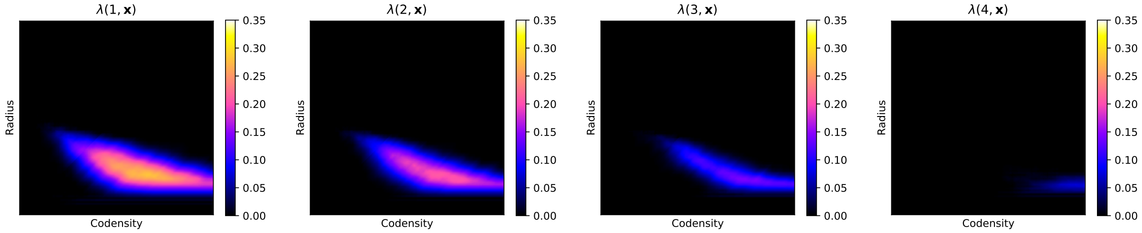

(d) Average Persistence Landscapes for 3 Rings Point Clouds taken over 30 samples.

Fig. S7. MPH-landscapes of two point clouds sampled from either two discs of different radius or three discs of the same radius and colored by the codensity value of the points. We note that this Figure is identical to Figure 2A,C from the main text; it is repeated here so that the supplementary information can be read as a self-contained document.

of tumor cells as macrophages at each timestep of the simulation. There are ∼2000 tumor cells and ∼100 macrophages at each timestep. Thus the 1% misregistering introduces ∼20 false macrophages cells at each timestep.

See Figure S8b for an example of an ABM simulation with this measurement error. The resulting decay curves in Figure S8e display the sensitivity of single parameter persistence to the incorrectly registered tumor cells. Using multiparameter persistence techniques we produce decay curves for the simulation data; this analysis is more robust to the measurement error since filtering by codensity reduces the impact of outliers on our topological summaries. At each timestep we produce the radius-codensity filtration on the point cloud of macrophages, compute the H_1-landscape for this bifiltration and integrate the square of the landscape function over the region R for which we have computed $\lambda(1, \mathbf{x})$: $\left(\int_R \lambda(1, \mathbf{x})^2 d\mathbf{x}\right)^{\frac{1}{2}}$. This can be thought of as approximately taking the norm of the H_1-landscape function since we consider the landscape functions living in $L^2(\mathbb{N} \times \mathbb{R}^n)$. The resulting decay curves are displayed in Figure S8g. The decay curves from the multiparameter analysis more closely resemble the decay curves without noise (Figure S8d), particularly at later times in the simulation, when the infiltration is significant. Other traditional spatial statistics are also unable to adequately describe macrophage structure in these simulations (in addition to the PCF examples shown in Figure 1, other traditional spatial statistics applied to the simulations in Figure S8 can be seen at https://github.com/JABull1066/SyntheticDataSpatialStats).

C. Comparison to 1PH Noise Reduction Techniques. The challenge of increasing the robustness of the persistent homology of a point cloud P has been previously addressed with a number of techniques. Broadly, these techniques involve either subsampling the point cloud with landmarks, or using the point cloud to induce a filtration on the ambient space in which the point cloud lies. We summarize several of these techniques in Table S2, and illustrate the result of these techniques on an example point cloud in Table S3.

The example point cloud used in Table S3 consists of rings with radii $\frac{1}{2}, 1$ and centers $(-1, 0), (0.5, 0)$ respectively, with 80 points sampled uniformly from the rings and a further 20 points uniformly sampled from the discs they enclose representing noise.

We see in Table S3 that MPH-landscapes capture the two predominant H_1 features in the point cloud. Using the Rips Filtration directly on the point cloud the resulting barcode does not detect the large ring, since all bars die before radius 0.5 and neither does the barcode have two clear features. The max-min sampling (64) preferentially samples outlier points and again we do not recover the two features of the point cloud.

The random sampling technique (65) requires a choice of the number of points k to sample. If we choose too few points the features have a late birth time and small persistence as the rings are not sampled densely enough, and if we choose too many points the chance of selecting a disruptive outlier point increases. For a well chosen number of points, in this case $k = 20$, the two features are recovered.

The power distance to measure (66) technique is sensitive to the choice of the mass parameter m. Tuning this parameter we found that $m = 0.1$ results in a barcode which detects both features. To compute the sublevel set persistent homology we use the lower-star filtration of the function $d^P_{\mu,m}$ on the Freudenthal triangulation on a meshgrid of 500×500 points in the region $[-2, 2] \times [-1.5, 1.5]$ using the Dionysus2 software package (https://mrzv.org/software/dionysus2/).

Whilst the refinements of 1PH have their merits, in this work we use MPH-landscapes, motivated by exploring the descriptive capability of this new technique, and the desire to compare bifiltrations with codensity and oxygen environment as a second parameter.

D. Quantifying Immune Cell Infiltration.

Spatial Distributions of CD8⁺, FoxP3⁺ and CD68⁺ Immune Cells. In this section we use multiparameter persistent homology techniques to analyze the spatial distribution of $CD8^+$, $FoxP3^+$ and $CD68^+$ cells within head and neck tumors; the behavior of these cells is biologically interesting and may be of prognostic significance. By looking at infiltration across multiple (>50) small 1.5mm × 1.5mm regions of interest we can make statistically evidenced qualitative observations about the comparative behaviors of the three types of immune cells. Taking this approach avoids confounding effects that may arise if individual sample regions are compared. Such confounding effects can arise because the H_1-landscape will fail to detect the largest potential immune cell annulus if this cuts the boundary of the region of interest or if the size of the tumor specimen on the slide (itself dependent on the position and orientation of each individual section within the 3D tissue) is limiting.

As noted by (48), in applications of multiparameter persistence it is important that the filtration function is appropriately standardized so that the parameters occur at similar scales. We apply two procedures to our point

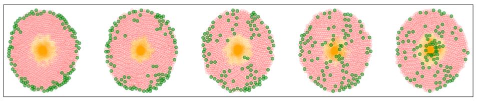

(a) Snapshots of an ABM simulation. Immune cells (green) are introduced on the boundary of a disc of tumor cells (red) and dying tumor cells (orange). The immune cells are drawn to the center of the tumor as the simulation progresses.

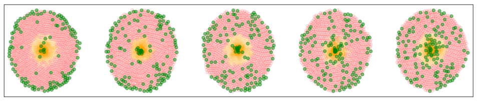

(b) Snapshots of an ABM simulation with measurement error. We misregister 1% of the tumor cells as macrophages (green) independently at each timestep.

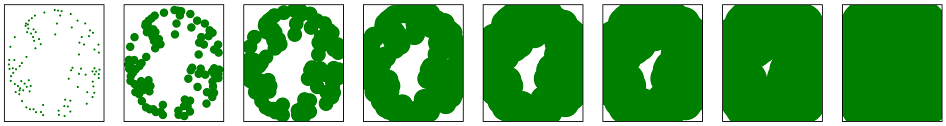

(c) The Čech filtration for the immune cell point cloud at a fixed time witnesses that the immune cells form an annulus for a wide range of parameter values.

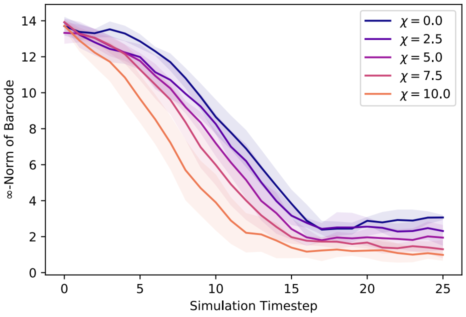

(d) Decay curves tracing the ∞-norm of the H_1-barcode of the macrophages against time.

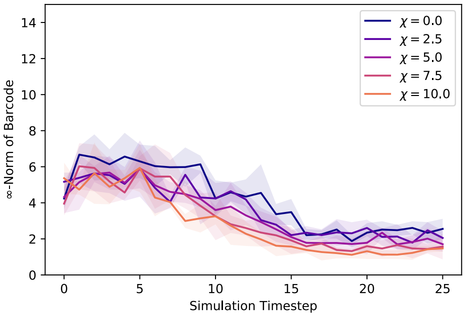

(e) Decay curves tracing the ∞-norm of the H_1-barcode of the macrophages with measurement error against time. The prominence of the detected annulus is severely diminished by the outliers introduced.

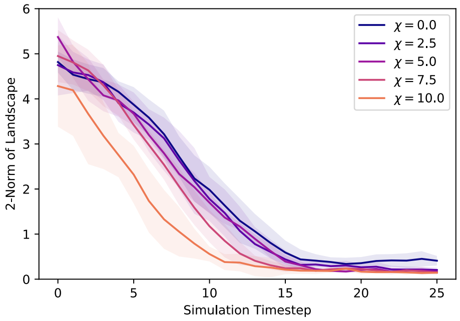

(f) Each decay curve traces the 2-norm of the H_1 MPH-landscape for the radius-codensity bifiltration of the macrophages against time.

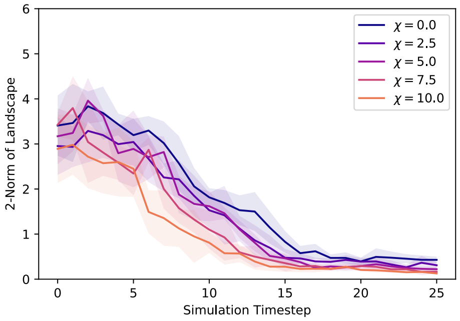

(g) Each decay curve traces the 2-norm of the H_1 MPH-landscape for the radius-codensity bifiltration of the macrophages with measurement error against time.

Fig. S8. Persistent homology analysis of ABM data with and without measurement error. We plot decay curves for 5 different values of the chemotaxis parameter $\chi \in \{0, 2.5, 5, 7.5, 10\}$ for simulations with no measurement error (d,f) and with measurement error (e,g). Each curve is averaged over 5 simulations for each chemotaxis parameter value with standard deviation bands depicted. We apply single parameter and multiparameter persistent homology to the ABM data with and without measurement error and note that the multiparameter methodology is more robust than the single parameter methodology to the introduction of measurement error. The 'simulation timesteps' presented here represent observations of cell locations at four hourly intervals over the 100 hour period which starts when the macrophages are introduced into the simulation. We note that panels (d), (e), (f) and (g) are identical to Figure 1C, F from the main text; they are repeated here so that the supplementary information can be read as a self-contained document.

Technique	Description	Advantages	Disadvantages
Max-min Sampling (64)	A set of k landmark points are chosen sequentially from the point cloud P. The first point is chosen uniformly at random from the set of points P and successive points are chosen to maximize the minimum distance of the next chosen point to all of the previously chosen points. This results in k landmark points well spread in the point cloud P. We then compute the persistent homology of this set of landmark points.	• Reduces point cloud size resulting in cheaper persistent homology computations.<br>• Sampling method is easy to compute.<br>• Produces more dispersed landmarks than random sampling.	• Unsuitable if outliers affect the persistent homology.<br>• Sensitive to the choice of the number of landmarks to draw.
Random Sampling (65)	A set of k landmark points are chosen from the point cloud P with respect to the empirical measure on P (that is points are chosen uniformly with replacement). The persistent homology of this set of landmarks is computed.	• Reduces point cloud size resulting in cheaper persistent homology computations.<br>• Sampling method is easy to compute.<br>• Outliers are unlikely to be selected as landmarks.<br>• The distribution of barcodes attained by this sampling is "uniformly robust" (65)	• Landmarks are less dispersed than max-min sampling.<br>• Sensitive to the choice of the number of landmarks to draw.
Power Distance to Measure (66)	Let (X, d_X) be a metric space with point cloud $P \subset X$, μ a measure on X, and $m \in (0, 1]$ a mass parameter. Define functions $X \to \mathbb{R}$:<br>• $\delta_{\mu,m}(x) = \inf\{r \geq 0 : \mu(B(x, r)) > m\}$<br>• $d_{\mu,m}(x) = \sqrt{\frac{1}{m}\int_0^m \delta_{\mu,l}(x)^2 dl}$<br>• $d^P_{\mu,m}(x) = \min_{p \in P} \sqrt{d_{\mu,m}(p)^2 + d_X(x, p)^2}$<br>Compute the sublevel set persistent homology of the function $d^P_{\mu,m} : X \to \mathbb{R}$.	• Satisfies stability results (66).<br>• Efficient to compute.<br>• Approximates distance to measure function which can be difficult to compute.	• Sensitive to choice of mass parameter m.<br>• Requires a triangulation of underlying metric space X for the sublevel set computation.

Table S2. Advantages and disadvantages of techniques used to improve robustness of persistent homology to noise.

Rips-Codensity Bifiltration H_1 MPH-landscapes (48)

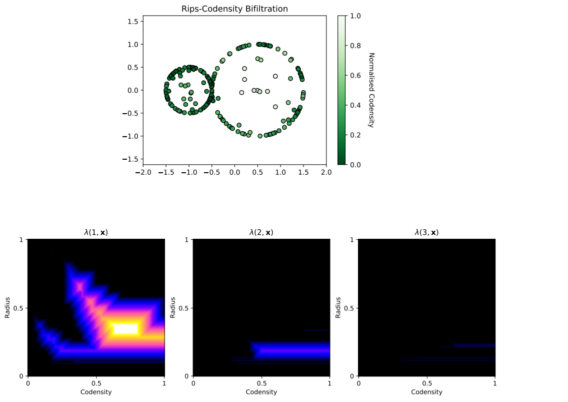

Rips Filtration

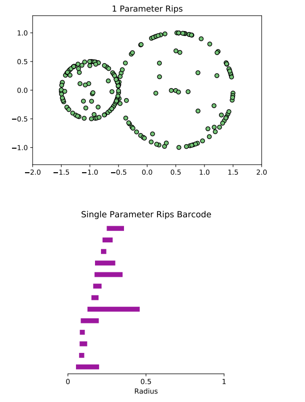

Max-min Sampling (64)

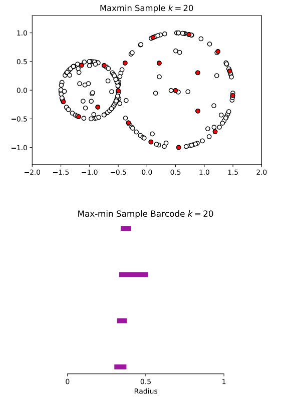

Random Sampling (65)

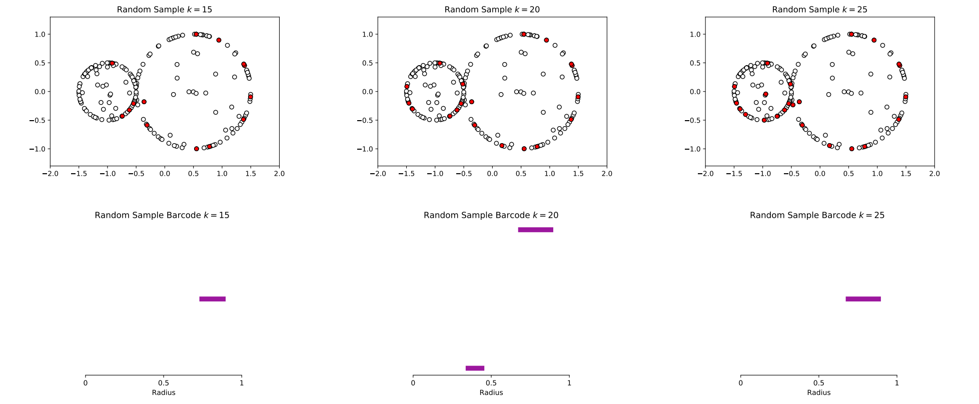

Power Distance to Measure (66)

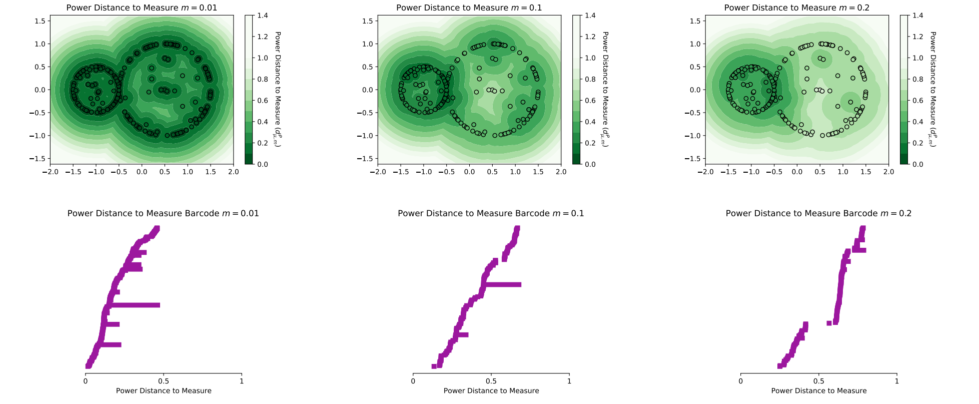

Table S3. Summary of persistent homology noise reduction techniques.

clouds so that the radius and codensity parameters occur at similar scales. We outline the procedures and their relative merits:

Region Standardization: We rescale each immune cell point cloud to have unit variance. We label each point $p \in \mathbb{R}^2$ with codensity function $\rho_{10}(p) = \frac{1}{10}\sum_{i=1}^{10} \|p - p_{(i)}\|_2$ where $p_{(i)}$ is the ith nearest neighbor of p. We standardize the codensity parameter by linearly rescaling the parameter so that 95% of values lie in the range $[0, 1]$ and 2.5% lie above and below this range. This standardization procedure allows us to compare heterogeneous samples which may have vastly different numbers of cells. Without this standardization, the comparison of samples containing vastly different numbers of cells would be dominated by the different ranges of the codensity parameter values. A disadvantage of this approach is that each point cloud is standardized with a different rescaling factor dependent on the density of cells and distribution of codensity parameter values.

Global Standardization: We rescale all immune cell point clouds by a scale factor of $\frac{1}{600}$ to have approximately unit variance. We label each point $p \in \mathbb{R}^2$ with codensity function $\rho_{10}(p) = \frac{1}{10}\sum_{i=1}^{10} \|p - p_{(i)}\|_2$ where $p_{(i)}$ is the ith nearest neighbor of p. We standardize the codensity parameter by rescaling by a factor of $\frac{1}{360}$ so that the majority of the values lie in the range $[0, 1]$. This standardization procedure preserves a real world interpretation of the filtration parameter values and is consistent across samples. A disadvantage of this approach is that we are required to set a global rescaling factor for the point clouds and codensity parameter.

After standardization we then compute the multiparameter persistence module in the region $[0, 1]^2$ for the standardized point cloud samples. We next integrate the multiparameter persistence landscapes over the parameter range $R_{>0.4} = \{(x_{\text{codensity}}, y_{\text{radius}}) : y_{\text{radius}} > 0.4\}$ so that each sample produces an $\mathbb{R}$-valued statistic: $\int_{R_{>0.4}} \lambda(1, \mathbf{x}) d\mathbf{x}$ which we call the *large loop statistic*. We then use traditional statistical techniques to compare the $\mathbb{R}$-valued statistics for the samples for each group.

We display the point clouds for each cell type (Figure S9 a-c) and the output of this analysis for a particular tumor (T_C from our cohort of 16 tumors). Examining the persistence landscapes from the different cell types (Figure S9d-h) , we observe that the persistence landscapes for the immune cell types have supports in different parts of the parameter space. We observe that the $CD8^+$ and $FoxP3^+$ cell samples contain loops with large persistence in both the radius and codensity parameters. In contrast, the $CD68^+$ cell samples form comparatively smaller loops supported on a smaller range of radius parameters.

The boxplots in Figure S9h display the distributions of the large loop statistic for the H_1-landscapes for the samples from this tumor. We analyze the statistical significance of the difference between the large loop statistics for the cell types. We apply pairwise two-sided permutation tests for the groups of CD8, FoxP3 and CD68 samples, with null hypothesis that the mean of the large loop statistics coincide.

In Tables S4–S9 we display the output of the same analysis applied to all of the tumors in the cohort using each standardization method. The results of our analysis are broadly similar for both the region and global standardization techniques. Table S4 and S7 contain the tumors from which we could derive > 50 regions of interest and Tables S5, S6 S8 and S9 contain the tumors from which fewer samples could be drawn. The analysis of many of the tumors is confounded by the large variance due to the small number of regions available.

The columns of the tables contain the following information:

- Hypoxia (% 1 d.p.): Percentage across all regions of interest labeled with the respective hypoxia marker
- $CD8^+$ Cells, $FoxP3^+$ Cells, $CD68^+$ Cells: The extracted point clouds for each immune cell type.
- CD8, FoxP3, CD68 $\bar{\lambda}(1, \mathbf{x})$: The mean first MPH-landscape for each immune cell type.
- Radius Profile: The mean first MPH-landscape summed over the codensity parameter.
- Large Loop Statistic Box Plots: The distributions of the large loop statistics for each cell type for this tumor.
- p-values: The approximate p-values for the pairwise two-sided permutation tests applied to the large loop statistics computed over $20,000$ iterations.

Examination of the summaries in Table S4 reveals an interesting change in behavior of the spatial patterning as the oxygenation of the tumor changes. For $FoxP3^+$ cells, as the oxygenation of the tumor decreases the radius of the loops that these cells support increases (see the shift in the peak of the radius profiles). In contrast, the radius profile for the CD68 labelled samples remains unchanged as the oxygenation varies. This behavior is consistent with the idea that T cells are excluded from hypoxic regions of the tumor whereas $CD68^+$ cells are not.

Moreover we notice that the relative height of the peak in the radius profile for the FoxP3 cells diminishes for the hypoxic tumors. The average landscapes reveal that this is due to an increase in the codensity parameter at which

the FoxP3$^+$ loops form. That is to say, the loops are supported over a smaller range of codensity parameters. Thus, the radius profile which is the sum of the landscape over the codensity parameters, has smaller size.

Of course, for such a small cohort these observations cannot be statistically verified, however they are consistent with a patterning phenomenon of potential biological interest which merits further exploration in a larger dataset.

We apply PCA to the collection of H_1 landscape vectors $\lambda(1, \mathbf{x})$ for tumors T_A–T_E (Figure S10a,b). Our PCA projections add further evidence that the spatial patterning of the CD8$^+$ and FoxP3$^+$ cells are more similar than the CD68$^+$ cells. We also apply LDA (Figure S10c,d) which clearly discriminates between the three cell types.

We test the ability of the MPH-landscape to distinguish the cell types in each tumor. For each pair of cell types we make a randomized 80/20 training/test split, and evaluate the classification accuracy of 3 classifiers (Linear Discriminant Analysis, LDA, Regularised Linear Discriminant Analysis, rLDA, and regularised Quadratic Discriminant Analysis, rQDA) on the test data. Repeating this process 100 times we attain average pairwise classification accuracies (Table S10). Our results indicate that the classifiers are most significant when used to compare CD68$^+$vs FoxP3$^+$and CD8$^+$vs FoxP3$^+$immune cells. Using both the first and second MPH-landscapes, $(\lambda(1, \mathbf{x}), \lambda(2, \mathbf{x}))$, marginally improves these classification accuracies.

Codensity and Oxygen Environment. In this section, we supplement the data presented in Figure 4 of the main text. We again use a bootstrapping technique to compare two bifiltrations on a large area of tumor in order to investigate the validity of using codensity as a proxy for hypoxia in topological analysis.

One way to quantify immune cell infiltration is by counting the number of immune cells in each oxygen environment and their distance to that region's boundary. This methodology requires oxygen staining data which may not be available. In contrast, the radius-codensity filtration only uses the spatial distribution of the immune cells and the assumption that immune cells are more densely packed in stromal regions compared to tumor regions to infer the degree of immune cell infiltration.

We test the use of codensity as a proxy for hypoxia by comparing the radius-codensity and radius-hypoxia bifiltrations on the same regions of tissue (see Figure S11). We analyze the H_1-multiparameter persistence landscapes associated to the two bifiltrations for the three different cell types: CD8$^+$, FoxP3$^+$ and CD68$^+$. The cell locations are displayed in Figure S11a together with labels on each cell marking increasingly hypoxic oxygen levels (Stroma, PanCK, CAIX, Pimo and Necrosis).

The region of interest contains too many cells for direct application of our multiparameter persistence techniques: the point clouds are too large for the multiparameter persistence computations to be tractable. However, we can overcome the large point cloud size ($\sim$8000 cells) using the Central Limit Theorem (Theorem 6) for multiparameter persistence landscapes. We repeatedly subsample the large point cloud and use the fact that the mean of the distribution will converge to the mean of the empirical distribution. In particular, for each cell type we take 50 subsamples of size 1500 and compute the distribution of 1-norms for the multiparameter persistence landscapes for the radius-codensity (Figure S11b) and radius-hypoxia (Figure S11c) bifiltrations. The distributions of the 1-norms of the landscapes for each cell type are summarized in Figures S11e and S11d.

Explicitly, the two bifiltrations we use are constructed as follows. First we rescale each immune cell point cloud subsample to have unit variance. For the radius-codensity bifiltration, we label each point $p \in \mathbb{R}^2$ with codensity function $\rho_{10}(p) = \frac{1}{10}\sum_{i=1}^{10} \|p - p_{(i)}\|_2$ where $p_{(i)}$ is the ith nearest neighbor of p. We standardize the codensity parameter by linearly rescaling the parameter so that 95% of values lie in the range $[0, \frac{1}{2}]$ and 2.5% lie above and below this range. For the radius-hypoxia filtration we label each point with the hypoxia stain indicating the strongest hypoxia. We convert the hypoxia labels to hypoxia parameter values by uniformly distributing the values in the range $[0, \frac{1}{2}]$: Stroma $= 0$, PanCK $= 0.1$, CAIX $= 0.2$, Pimo $= 0.3$ and Necrosis $= 0.4$. For both bifiltrations we take the maximum radius parameter to be $\frac{1}{2}$, and we compute the multiparameter persistence module and landscape in the parameter region $[0, \frac{1}{2}]^2$.

We plot the radius-codensity (Figure S11b) and radius-hypoxia (Figure S11c) bifiltrations for the FoxP3 cells. Note the similarity between the filtrations. In the hypoxia filtration we first introduce cells in the stromal region (blue) and gradually introduce cells closer to the center of the tumor region with their increasingly severe hypoxic environments. Introducing the more hypoxic cells gradually fills in the loop of unstained cells that surrounds the tumor region. Similarly, in the codensity filtration we first introduce densely packed cells in the stromal region and gradually introduce the more sparsely packed cells closer to the center of the tumor.

Both the radius-codensity (Figure S11b) and radius-hypoxia (Figure S11c) bifiltrations identify the fact that CD68 cells infiltrate the hypoxic region to a greater extent than the CD8$^+$ and FoxP3$^+$ cells, as witnessed by the smaller norms of the CD68 H_1-landscapes. The same phenomenon is observed when we repeat the analysis for another hypoxic region using the same techniques (Figure S12).

To supplement our topological analysis we examine the interaction between the oxygen environment and codensity

of each cell type. We label each cell with a hypoxia score given by the most severe hypoxia condition determined by the staining taken up by that cell: stroma: 0, PanCK: 1, CAIX: 2, Pimo: 3 and necrosis: 4.

We extend the hypoxia score to any point in the image by taking a weighted average of the 10 nearest cells. Suppose $c_1, ..., c_{10}$ are the 10 closest cell location to point p in the tumor image with hypoxia scores $h_1, ..., h_{10}$ we define the hypoxia score of p to be $\frac{1}{\sum_{j=1}^{10} e^{-\sigma\|(p-c_j\|_2}} \sum_{i=1}^{10} h_i e^{-\sigma\|(p-c_i\|_2}$.

We sample a meshgrid of points across the region (clipped away from the edge of the image to mitigte edge effects on the codensity function) and record the codensity score ρ_{10} and hypoxia score for these points. We use locally weighted scatterplot smoothing (a non-parametric regression method) to extract the change in codensity across hypoxic conditions for each cell type and two large regions of tissue with diverse hypoxic conditions (Figure S13).

Our observations are consistent with the topological analysis, showing that CD68$^+$cells infiltrate the hypoxic regions of the tissue to a greater extent than the T-cells, as seen by the lower codensity (higher density) of the CD68$^+$cells in regions of the tissue with hypoxia scores indicating Pimo staining (3.0) and necrosis (4.0).

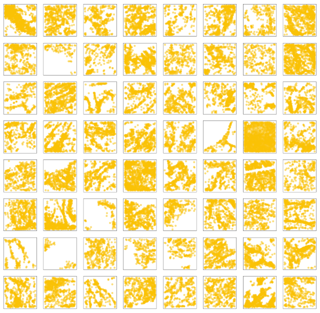

(a) Point clouds of CD8⁺ cells for 64 sample regions.

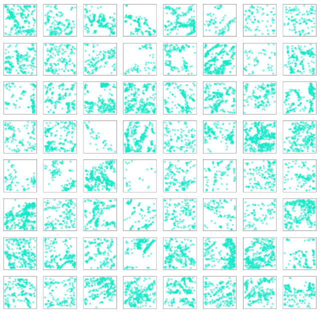

(b) Point clouds of FoxP3⁺ cells for 64 sample regions.

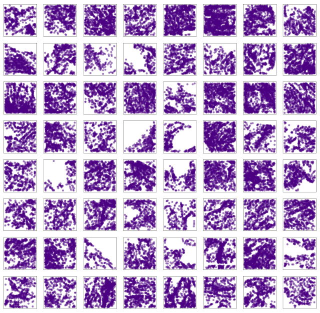

(c) Point clouds of CD68⁺ cells for 64 sample regions.

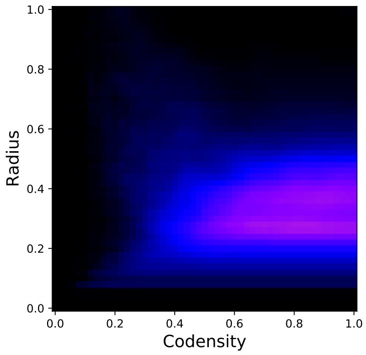

(d) The mean of the first H_1 multiparameter persistence landscape for the CD8⁺ cell sample regions.

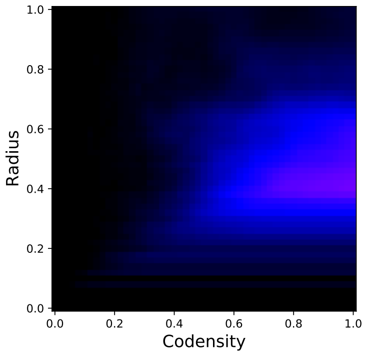

(e) The mean of the first H_1 multiparameter persistence landscape for the FoxP3⁺ cell sample regions.

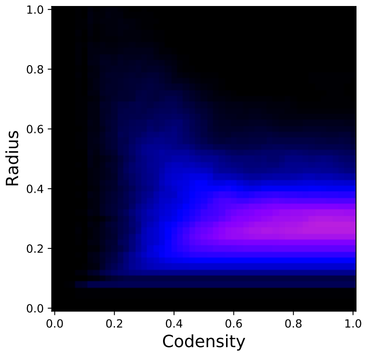

(f) The mean of the first H_1 multiparameter persistence landscape for the CD68⁺ cell sample regions.

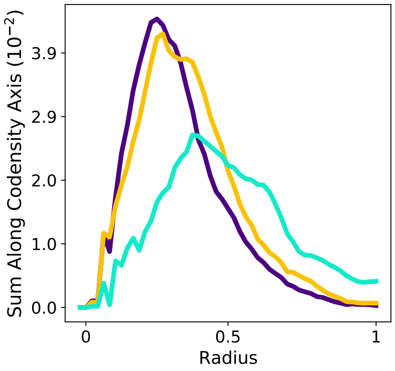

(g) The mean H_1 landscapes summed over the codensity parameter. We see that on average the CD8⁺ cells (yellow) and FoxP3⁺ cells (teal) support loops of larger radius than the CD68⁺ cells (purple).

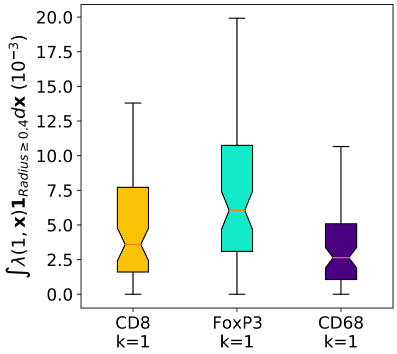

(h) Box plots comparing the large loop statistics (derived from the H_1 landscapes) of the samples of the three cell types.

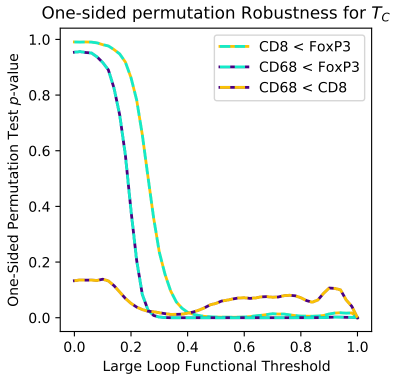

(i) One-sided permutation test p-values for a range of choices of radius threshold indicating the significant difference in the large loop functional distributions was not sensitive to the choice of 0.4 as the threshold.

Fig. S9. Comparison of immune cell spatial patterning within head and neck cancer tissue.

Table S4. Table of topological analysis for cohort of tumors using region standardization.

Tumor	Hypoxia (% 1 d.p.) Necrosis|Pimo|CAIX	$CD8^+$ Cells	$FoxP3^+$ Cells	$CD68^+$ Cells	CD8 $\bar{\lambda}(1, \mathbf{x})$	FoxP3 $\bar{\lambda}(1, \mathbf{x})$	CD68 $\bar{\lambda}(1, \mathbf{x})$	Radius Profile	Large Loop Statistic Box-plots	p-values $CD8^+$ vs $FoxP3^+$, $CD8^+$ vs $CD68^+$, $CD68^+$ vs $FoxP3^+$
T_A	0.1 | 1.9 | 0.1	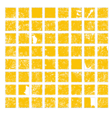	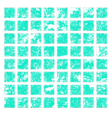	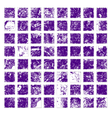	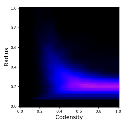	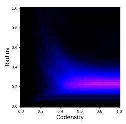	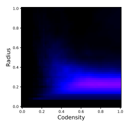	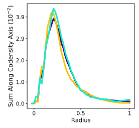	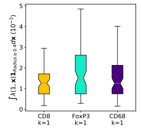	0.63600, 0.59370, 0.95240
T_B	0.8 | 3.5 | 24.6	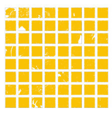	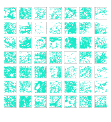	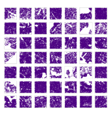	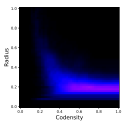	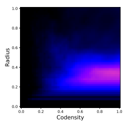	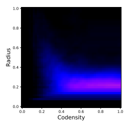	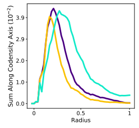	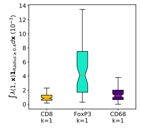	0.00000, 0.01490, 0.00000
T_C	3.9 | 9.3 | 12.6	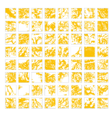	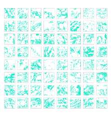	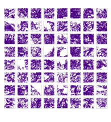	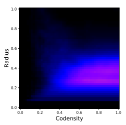	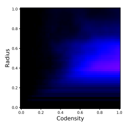	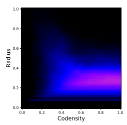	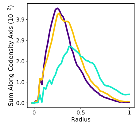	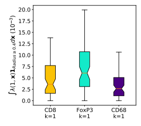	0.04190, 0.03315, 0.00000
T_D	6.0 | 5.8 | 16.0	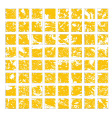	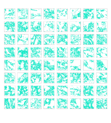	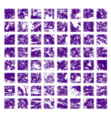	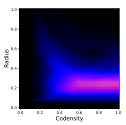	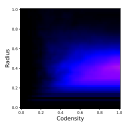	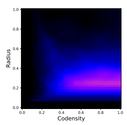	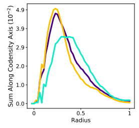	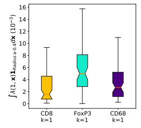	0.00930, 0.26075, 0.15135
T_E	6.3 | 13.3 | 23.5	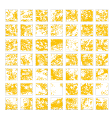	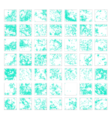	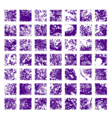	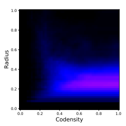	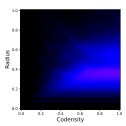	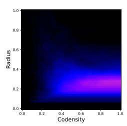	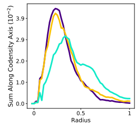	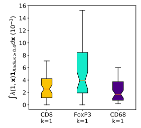	0.00750, 0.28495, 0.00065

Table S5. Table of topological analysis for cohort of tumors using region standardization.

Tumor	Hypoxia (% 1 d.p.) Necrosis|Pimo|CAIX	CD8+ Cells	FoxP3+ Cells	CD68+ Cells	CD8 $\bar{\lambda}(1,\mathbf{x})$	FoxP3 $\bar{\lambda}(1,\mathbf{x})$	CD68 $\bar{\lambda}(1,\mathbf{x})$	Radius Profile	Large Loop Statistic Box-plots	p-values CD8+ vs FoxP3+, CD8+ vs CD68+, CD68+ vs FoxP3+
T_F	1.4 | 6.6 | 8.0	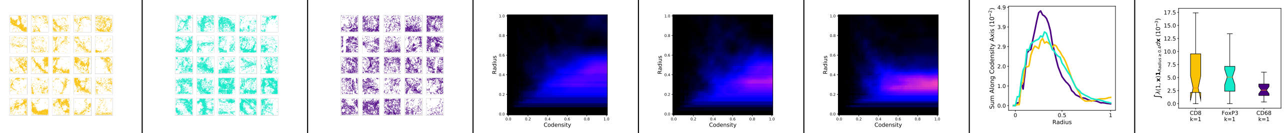								0.80785, 0.23545, 0.23130
T_G	5.8 | 2.2 | 12.6	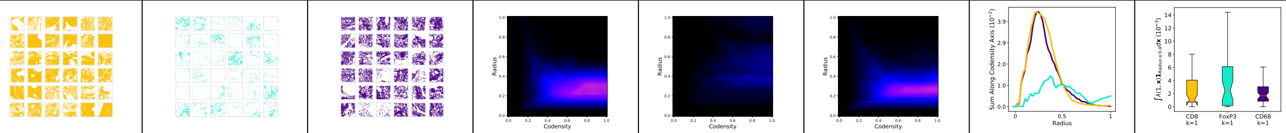								0.27740, 0.74280, 0.41820
T_H	1.5 | 1.6 | 1.7	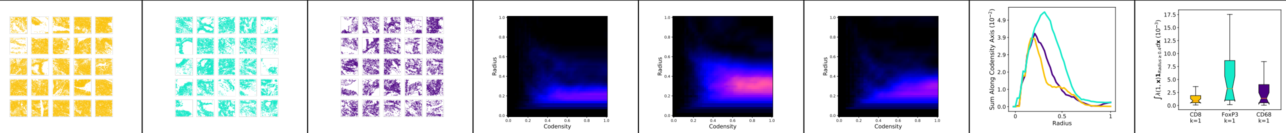								0.03505, 0.93485, 0.02525
T_I	0.0 | 14.2 | 15.2	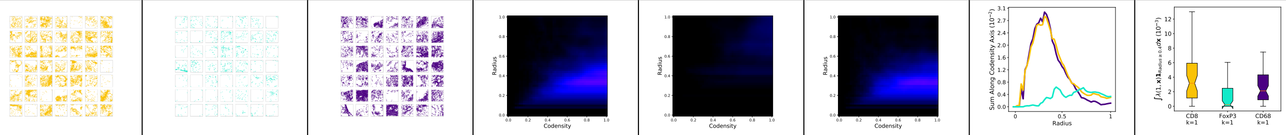								0.09825, 0.24965, 0.47935
T_J	3.8 | 4.8 | 1.9	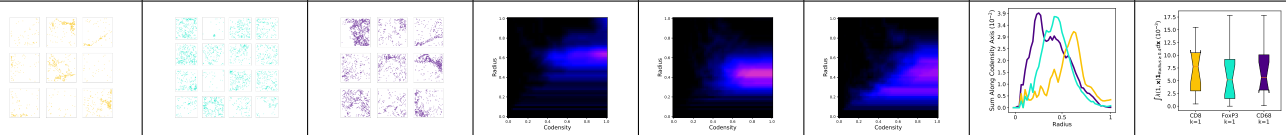								0.74750, 0.59330, 0.85650
T_K	0.1 | 2.1 | 0.7	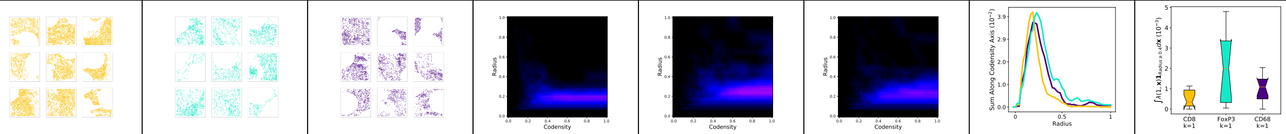								0.00950, 0.04325, 0.15435
T_L	0.0 | 3.8 | 3.6	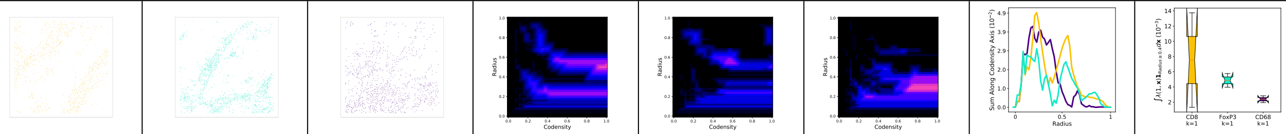								0.65825, 0.66680, 0.00000
T_M	8.6 | 2.2 | 0.8	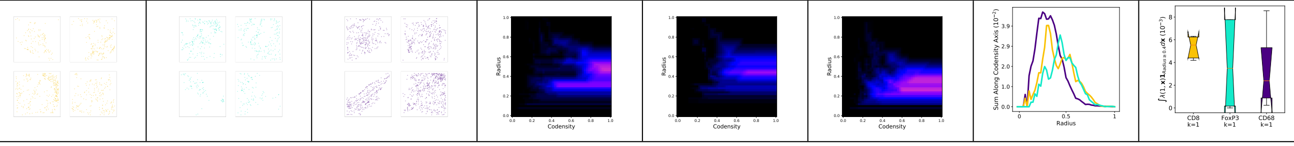								0.85565, 0.30695, 0.52855

Table S6. Table of topological analysis for cohort of tumors using region standardization.

Tumor	Hypoxia (% 1 d.p.) Necrosis|Pimo|CAIX	CD8$^+$ Cells	FoxP3$^+$ Cells	CD68$^+$ Cells	CD8 $\bar{\lambda}(1, \mathbf{x})$	FoxP3 $\bar{\lambda}(1, \mathbf{x})$	CD68 $\bar{\lambda}(1, \mathbf{x})$	Radius Profile	Large Loop Statistic Box-plots	p-values CD8$^+$ vs FoxP3$^+$, CD8$^+$ vs CD68$^+$, CD68$^+$ vs FoxP3$^+$
T_N	0.1 | 2.0 | 6.3		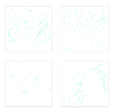	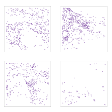	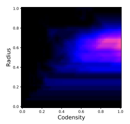	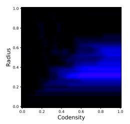	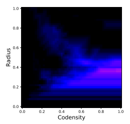	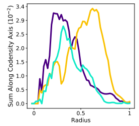	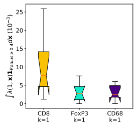	0.02225, 0.09050, 0.65065
T_O	0.9 | 8.1 | 1.4	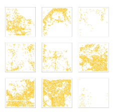	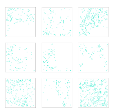	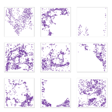	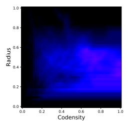	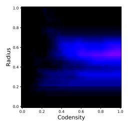	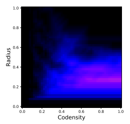	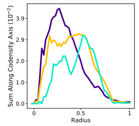	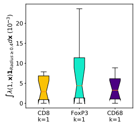	0.91855, 0.72825, 0.45240
T_P	0.0 | 1.1 | 0.4		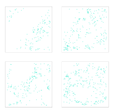	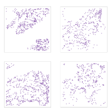	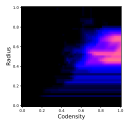	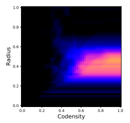	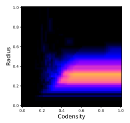	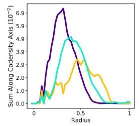	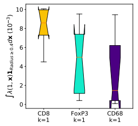	0.97505, 0.12090, 0.57065

Table S7. Table of topological analysis for cohort of tumors using global standardization.

Tumor	Hypoxia (% 1 d.p.) Necrosis|Pimo|CAIX	$CD8^+$ Cells	$FoxP3^+$ Cells	$CD68^+$ Cells	CD8 $\bar{\lambda}(1, \mathbf{x})$	FoxP3 $\bar{\lambda}(1, \mathbf{x})$	CD68 $\bar{\lambda}(1, \mathbf{x})$	Radius Profile	Large Loop Statistic Box-plots	p-values $CD8^+$ vs $FoxP3^+$, $CD8^+$ vs $CD68^+$, $CD68^+$ vs $FoxP3^+$
T_A	0.1 | 1.9 | 0.1	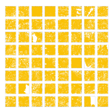	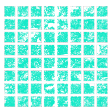	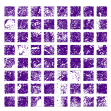	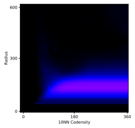	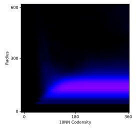	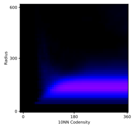	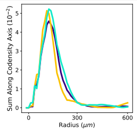	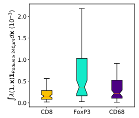	0.48385, 0.83185, 0.55905
T_B	0.8 | 3.5 | 24.6	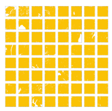	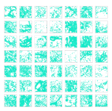	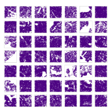	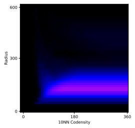	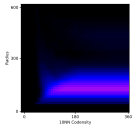	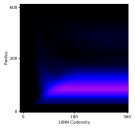	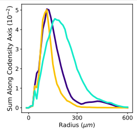	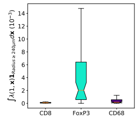	0.00000, 0.01515, 0.00160
T_C	3.9 | 9.3 | 12.6	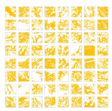	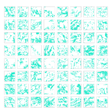	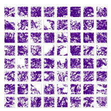	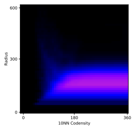	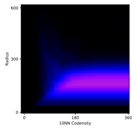	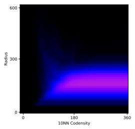	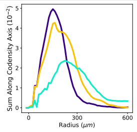	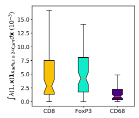	0.20840, 0.00000, 0.00000
T_D	6.0 | 5.8 | 16.0	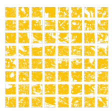	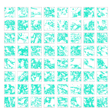	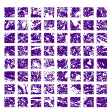	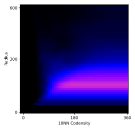	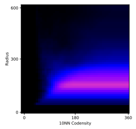	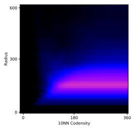	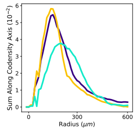	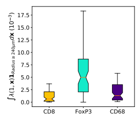	0.04550, 0.37665, 0.40100
T_E	6.3 | 13.3 | 23.5	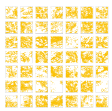	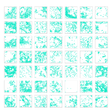	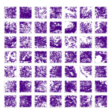	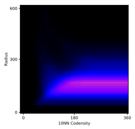	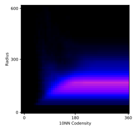	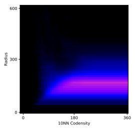	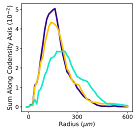	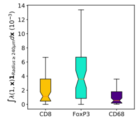	0.01230, 0.48800, 0.00430

Table S8. Table of topological analysis for cohort of tumors using global standardization.

Tumor	Hypoxia (% 1 d.p.) Necrosis|Pimo|CAIX	$CD8^+$ Cells	$FoxP3^+$ Cells	$CD68^+$ Cells	CD8 $\bar{\lambda}(1,\mathbf{x})$	FoxP3 $\bar{\lambda}(1,\mathbf{x})$	CD68 $\bar{\lambda}(1,\mathbf{x})$	Radius Profile	Large Loop Statistic Box-plots	p-values $CD8^+$ vs $FoxP3^+$, $CD8^+$ vs $CD68^+$, $CD68^+$ vs $FoxP3^+$
T_F	1.4 | 6.6 | 8.0	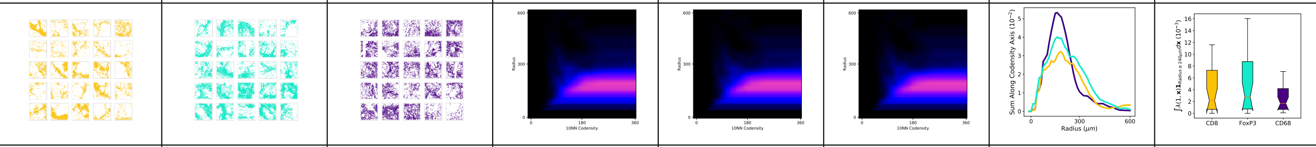								0.40800, 0.47015, 0.15735
T_G	5.8 | 2.2 | 12.6	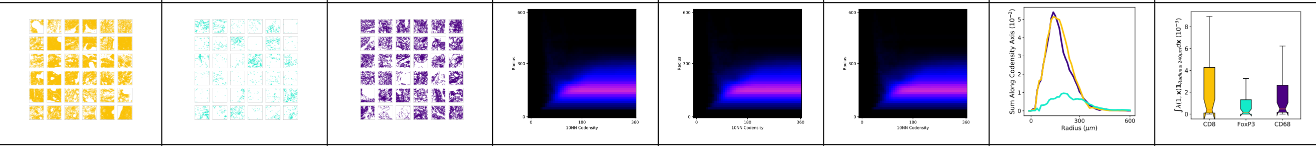								0.18290, 0.81250, 0.29160
T_H	1.5 | 1.6 | 1.7	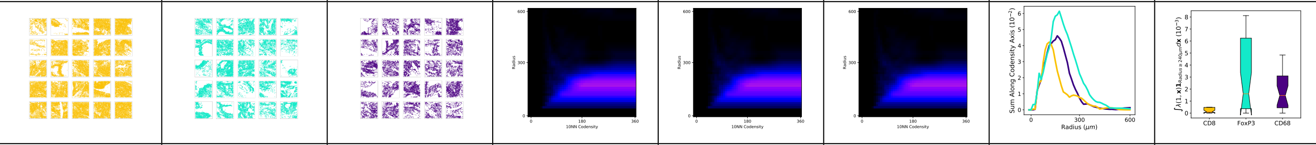								0.03580, 0.73770, 0.03530
T_I	0.0 | 14.2 | 15.2	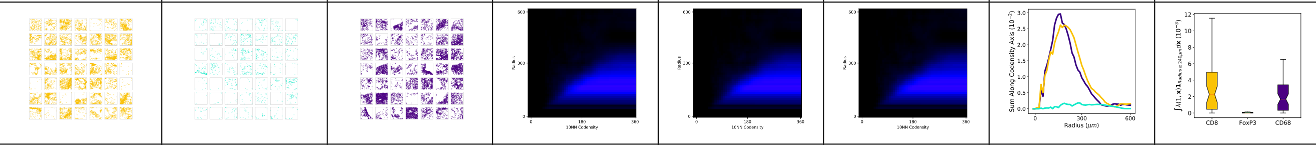								0.00000, 0.14400, 0.00005
T_J	3.8 | 4.8 | 1.9	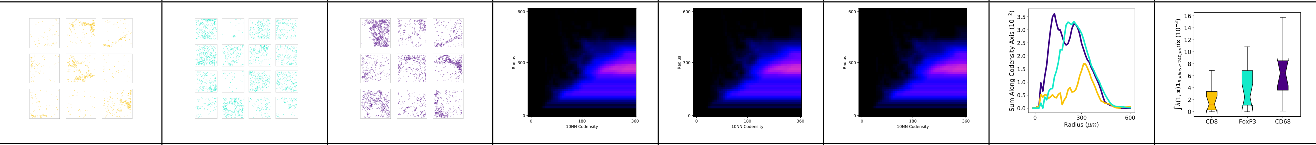								0.26695, 0.14245, 0.82960
T_K	0.1 | 2.1 | 0.7	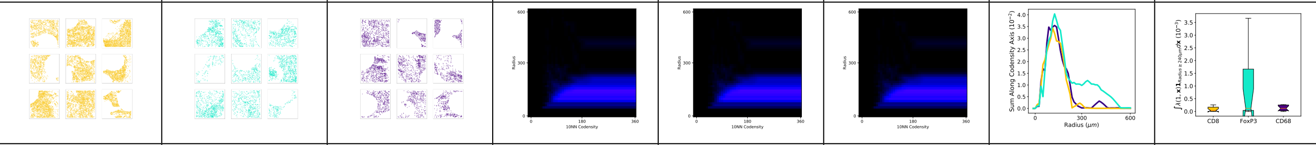								0.02620, 0.45205, 0.14625
T_L	0.0 | 3.8 | 3.6	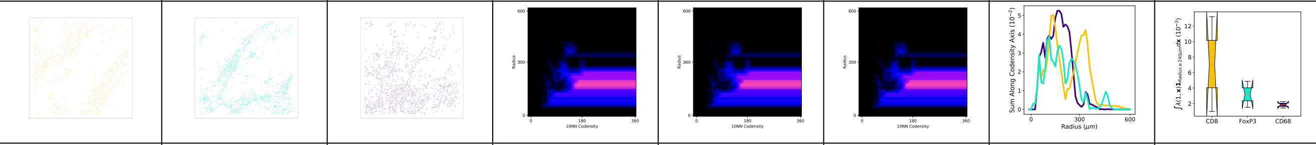								0.66370, 0.66995, 0.32860
T_M	8.6 | 2.2 | 0.8	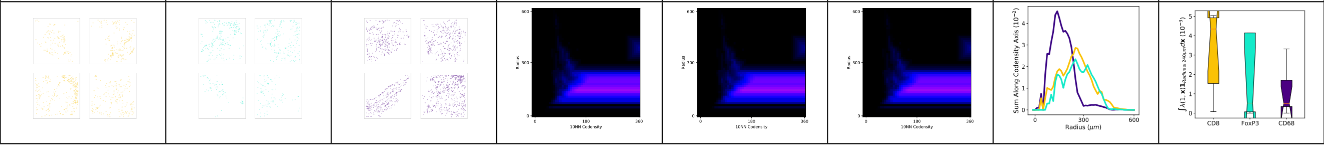								0.85020, 0.07005, 0.33850

Table S9. Table of topological analysis for cohort of tumors using global standardization.

Tumor	Hypoxia (% 1 d.p.) Necrosis|Pimo|CAIX	CD8⁺ Cells	FoxP3⁺ Cells	CD68⁺ Cells	CD8 $\bar{\lambda}(1, \mathbf{x})$	FoxP3 $\bar{\lambda}(1, \mathbf{x})$	CD68 $\bar{\lambda}(1, \mathbf{x})$	Radius Profile	Large Loop Statistic Box-plots	p-values $CD8^+$ vs $FoxP3^+$, $CD8^+$ vs $CD68^+$, $CD68^+$ vs $FoxP3^+$
T_N	0.1 | 2.0 | 6.3		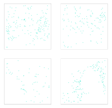	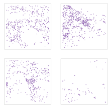	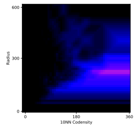	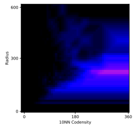	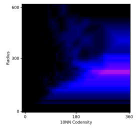	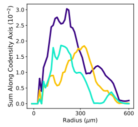	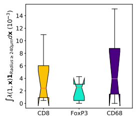	0.26995, 0.70175, 0.10160
T_O	0.9 | 8.1 | 1.4	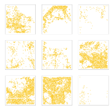	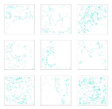	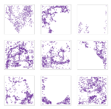	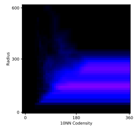	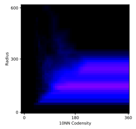	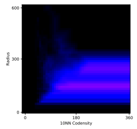	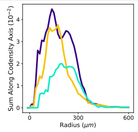	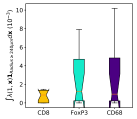	0.50550, 0.23115, 0.48980
T_P	0.0 | 1.1 | 0.4		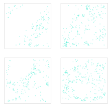	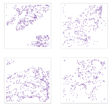	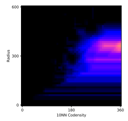	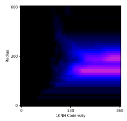	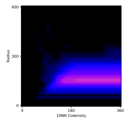	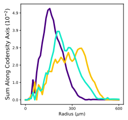	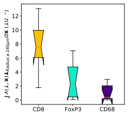	0.77680, 0.01055, 0.38795

	CD68$^+$ vs FoxP3$^+$			CD8$^+$ vs FoxP3$^+$			CD8$^+$ vs CD68$^+$			CD8$^+$ vs CD68$^+$ vs FoxP3$^+$		
Tumor	LDA	rLDA	rQDA	LDA	rLDA	rQDA	LDA	rLDA	rQDA	LDA	rLDA	rQDA
T_A	0.584	0.491	0.503	0.672	0.642	0.626	0.669	0.630	0.598	0.486	0.427	0.380
T_B	0.794	0.760	0.738	0.880	0.787	0.828	0.540	0.671	0.588	0.568	0.604	0.531
T_C	0.723	0.863	0.855	0.700	0.747	0.624	0.605	0.653	0.673	0.505	0.640	0.614
T_D	0.811	0.683	0.554	0.899	0.829	0.817	0.644	0.476	0.494	0.613	0.492	0.482
T_E	0.732	0.820	0.826	0.644	0.736	0.738	0.593	0.534	0.548	0.511	0.502	0.499
T_F	0.738	0.623	0.646	0.644	0.476	0.409	0.730	0.765	0.666	0.511	0.408	0.412
T_G	0.771	0.886	0.882	0.782	0.897	0.948	0.675	0.446	0.485	0.599	0.581	0.583
T_H	0.710	0.524	0.621	0.682	0.735	0.677	0.628	0.714	0.699	0.555	0.502	0.550
T_I	0.733	0.859	0.919	0.758	0.883	0.879	0.540	0.484	0.493	0.548	0.597	0.626
T_J	0.727	0.608	0.588	0.535	0.750	0.658	0.602	0.850	0.860	0.449	0.536	0.541
T_K	0.510	0.376	0.468	0.570	0.523	0.632	0.502	0.455	0.567	0.404	0.261	0.352
T_N	0.493	0.410	0.353	0.512	0.432	0.265	0.577	0.643	0.510	0.342	0.294	0.334
T_O	0.948	0.702	0.724	0.788	0.644	0.626	0.532	0.546	0.524	0.550	0.429	0.440

Table S10. Classification accuracy of three classifiers (LDA, rLDA, rQDA) in classifying the immune cell type of a sample using its first multiparameter persistence landscape (computed using global standardization). The classifiers are Linear Discriminant Analysis (LDA), Regularized Linear Discriminant Analysis (rLDA), and Regularized Quadratic Discriminant Analysis (rQDA). LDA and QDA are regularized by first projecting the high-dimensional landscape vectors to their first three principal components. The landscape vectors for each tumor are split into 80/20 train-test splits (unstratified) and classification accuracy is reported as the mean over 100 repetitions of splitting, training and testing. The tumors for which there were too few samples to produce a train-test split have been excluded.

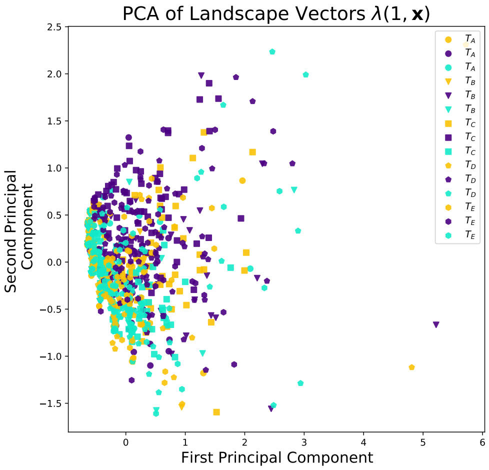

(a) PCA of H_1 landscape vectors $\lambda(1, \mathbf{x})$ computed with global standardization.

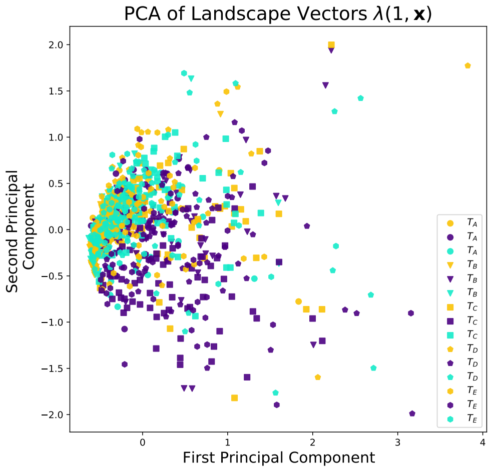

(b) PCA of H_1 landscape vectors $\lambda(1, \mathbf{x})$ computed with region standardization.

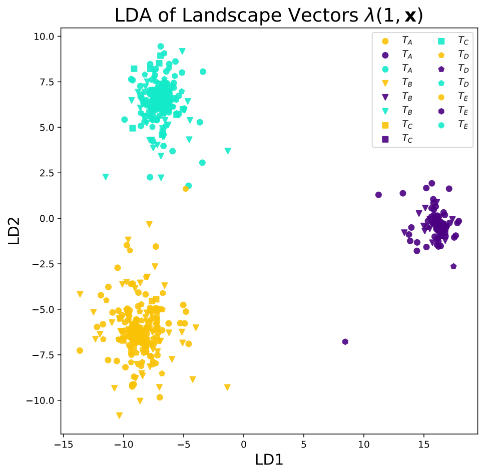

(c) LDA of H_1 landscape vectors $\lambda(1, \mathbf{x})$ computed with global standardization.

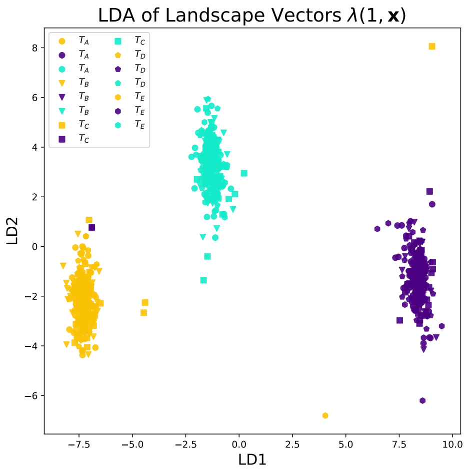

(d) LDA of H_1 landscape vectors $\lambda(1, \mathbf{x})$ computed region standardization.

Fig. S10. PCA and LDA plots of the radius-codensity landscape vectors $\lambda(1, \mathbf{x})$ for the CD8$^+$ (gold), FoxP3$^+$ (teal) and CD68$^+$ (purple) cell samples from tumors T_A, T_B, T_C, T_D, T_E. We compute the landscape vectors both with global standardization (left column) and with region standardization (right column).

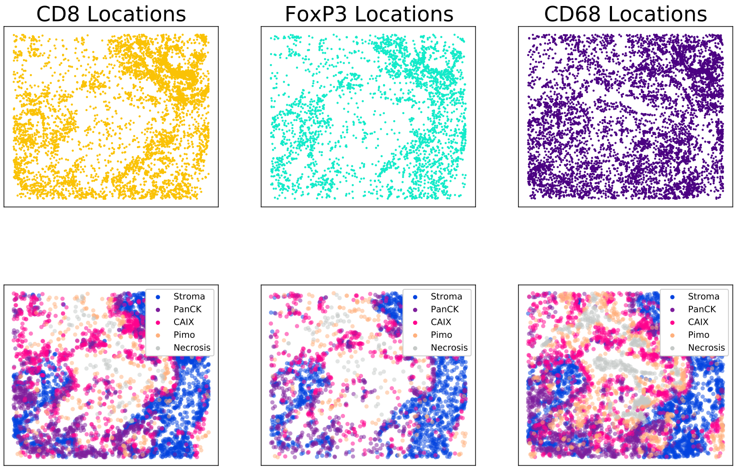

(a) Spatial distribution of CD8 (gold), CD68 (purple) and FoxP3 (teal) cells around a necrotic region. The immune cell point clouds labeled with oxygen environment: Stroma, PanCK, CAIX, Pimo and necrosis.

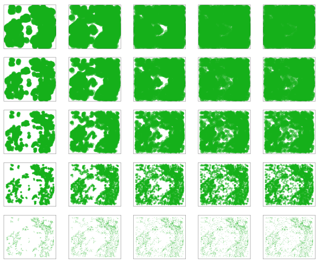

(b) The radius-codensity bifiltration associated to the FoxP3 point cloud.

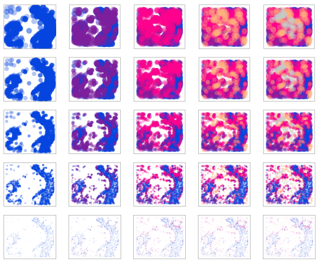

(c) The radius-hypoxia bifiltration associated to the FoxP3 point cloud.

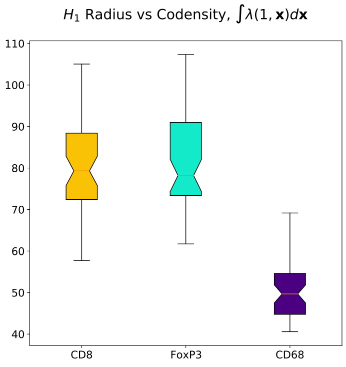

(d) The distribution of the 1-norms of the H_1 multi-parameter persistence landscapes associated to the radius-codensity bifiltration. We take 50 independent uniform subsamples of size 1500 from the original point clouds in Figure S11a.

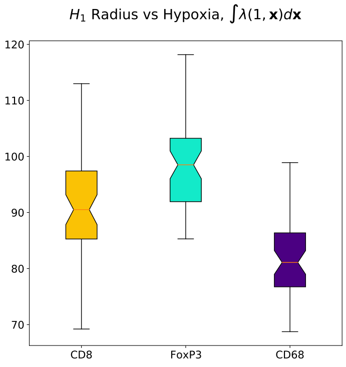

(e) The distribution of the 1-norms of the H_1 multi-parameter persistence landscapes associated to the radius-hypoxia bifiltration. We take 50 independent uniform subsamples of size 1500 from the original point clouds in Figure S11a

Fig. S11. Comparing radius-hypoxia and radius-codensity filtrations on a large region with central necrosis.

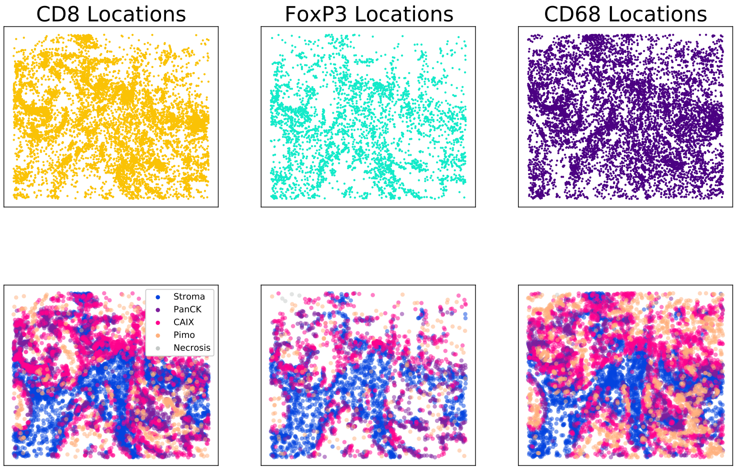

(a) Spatial distribution of CD8+ (gold), CD68+ (purple) and FoxP3+ (teal) cells. The immune cell point clouds labeled with oxygen environment: Stroma, PanCK, CAIX, Pimo and necrosis.

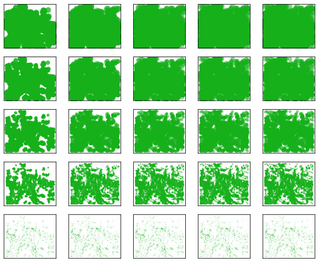

(b) The radius-codensity bifiltration associated to the FoxP3 point cloud.

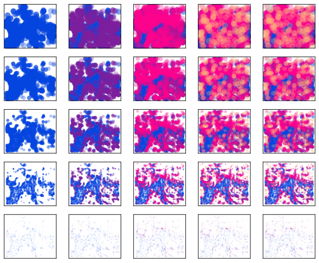

(c) The radius-hypoxia bifiltration associated to the FoxP3 point cloud.

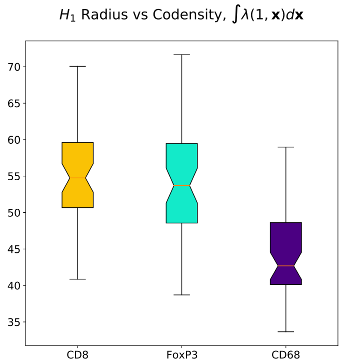

(d) The distribution of the 1-norms of the H_1 multi-parameter persistence landscapes associated to the radius-codensity bifiltration. We take 50 independent uniform subsamples of size 1500 from the original point clouds in Figure S12a.

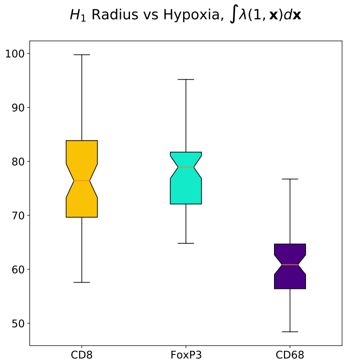

(e) The distribution of the 1-norms of the H_1 multi-parameter persistence landscapes associated to the radius-hypoxia bifiltration. We take 50 independent uniform subsamples of size 1500 from the original point clouds in Figure S12a

Fig. S12. Comparing radius-hypoxia and radius-codensity filtrations on a large region with hypoxia but little necrosis. The CD8+, FoxP3+and CD68+point cloud contains 9360, 3681, 8059 cells respectively. The dominant loop in both the radius-codensity and radius-hypoxia bifiltrations corresponds to the Pimo stained region in the top left of the cell point clouds.

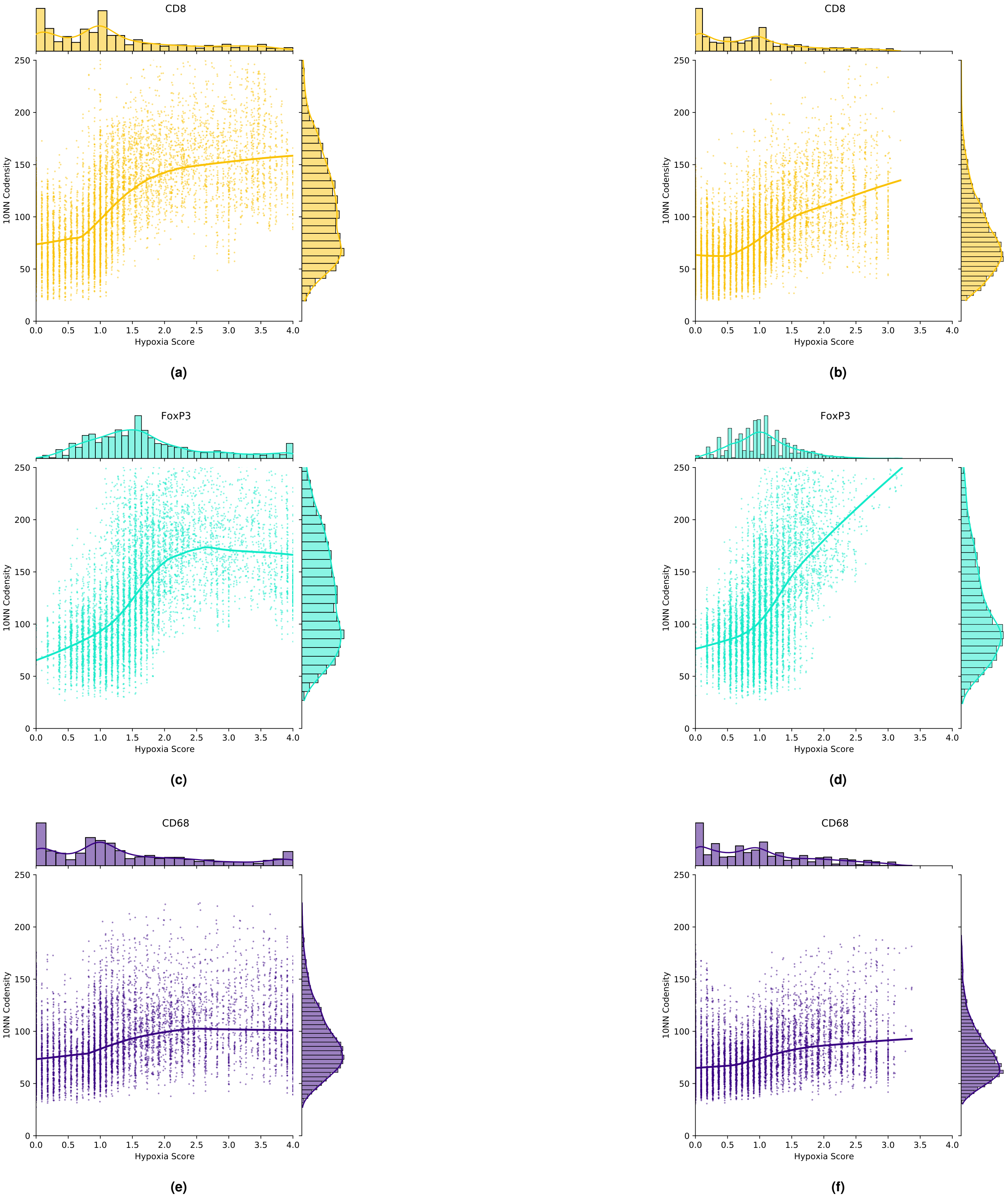

Fig. S13. Locally weighted scatterplot smoothing of cell codensity against hypoxia score for the poorly oxygenated tumor region depicted in Figure S11 (left column) and the better oxygenated region Figure S12 (right column). $CD68^+$ cells are present at higher density (lower codensity) than the T-cells ($CD8^+$ and $FoxP3^+$) in the hypoxic regions of the tissue with scores indicating Pimo staining (3.0) and necrosis (4.0).

References

1. G Mirams, et al., Chaste: An open source c++ library for computational physiology and biology. *PLoS Comput. Biol.* **9** (2013).
2. J Pitt-Francis, et al., Chaste: A test-driven approach to software development for biological modelling. *Comput. Phys. Commun.* **180**, 2452–2471 (2009).
3. JA Bull, F Mech, T Quaiser, SL Waters, HM Byrne, Mathematical modelling reveals cellular dynamics within tumour spheroids. *PLoS Comput. Biol.* **16** (2020).
4. R Leek, *The Role of Tumour Associated Macrophages in Breast Cancer Angiogenesis.* PhD Thesis, Oxford Brookes University (1999) https://oxfordbrookes.on.worldcat.org/oclc/53598498.
5. M Yang, D McKay, JW Pollard, CE Lewis, Diverse functions of macrophages in different tumor microenvironments. *Cancer Res.* **78** (2018).
6. RJ McMurtrey, Analytic models of oxygen and nutrient diffusion, metabolism dynamics, and architecture optimization in three-dimensional tissue constructs with applications and insights in cerebral organoids. *Tissue Eng. - Part C: Methods* **22**, 221–249 (2016).
7. A Greijer, E Van Der Wall, The role of hypoxia inducible factor 1 (HIF-1) in hypoxia induced apoptosis. *J. Clin. Pathol.* **57**, 1009–1014 (2004).
8. H Perfahl, et al., 3D hybrid modelling of vascular network formation. *J. Theor. Biol.* **414**, 254–268 (2017).
9. D Drasdo, S Höhme, A single-cell-based model of tumor growth in vitro: monolayers and spheroids. *J. Theor. Biol.* **2**, 133–147 (2005).
10. D Drasdo, M Loeffler, Individual-based models to growth and folding in one-layered tissues: intestinal crypts and early development. *Nonlinear Analysis* **47**, 245–256 (2001).
11. F Meineke, C Potten, M Loeffler, Cell migration and organization in the intestinal crypt using a lattice-free model. *Cell Prolif.* **34**, 253–266 (2001).
12. H Edelsbrunner, D Kirkpatrick, R Seidel, On the shape of a set of points. *IEEE Transactions on Inf. Theory* **29**, 551–559 (1983).
13. JM Osborne, AG Fletcher, JM Pitt-Francis, PK Maini, DJ Gavaghan, Comparing individual-based approaches to modelling the self-organization of multicellular tissues. *PLoS Comput. Biol.* **13**, 1–34 (2017).
14. S Laget, et al., Technical insights into highly sensitive isolation and molecular characterization of fixed and live circulating tumour cells for early detection of tumor invasion. *PLoS ONE* **12**, e0169427 (2017).
15. WF Mueller-Klieser, RM Sutherland, Oxygen tensions in multicell spheroids of two cell lines. *Br. J. Cancer* **45**, 256–264 (1982).
16. DR Grimes, C Kelly, K Bloch, M Partridge, A method for estimating the oxygen consumption rate in multicellular tumour spheroids. *J. Royal Soc. Interface* **11**, 20131124 (2014).
17. JL Landry, JP Freyer, RM Sutherland, Shedding of mitotic cells from the surface of multicell spheroids during growth. *J. Cell. Physiol.* **106**, 23–32 (1981).
18. N Jagiella, B Müller, M Müller, IE Vignon-Clementel, D Drasdo, Inferring growth control mechanisms in growing multi-cellular spheroids of NSCLC cells from spatial-temporal image data. *PLoS Comput. Biol.* **12** (2016).
19. P Pathmanathan, et al., A computational study of discrete mechanical tissue models. *Phys. Biol.* **6** (2009).
20. SJ Dunn, IS Näthke, JM Osborne, Computational models reveal a passive mechanism for cell migration in the crypt. *PLoS ONE* **8**, 1–18 (2013).
21. G Schaller, M Meyer-Hermann, Multicellular tumor spheroid in an off-lattice voronoi-delaunay cell model. *Phys. Rev. E* **71**, 1–16 (2005).
22. H Knutsdottir, JS Condeelis, E Palsson, 3-D individual cell based computational modeling of tumor cell-macrophage paracrine signaling mediated by EGF and CSF-1 gradients. *Integr. Biol.* **8** (2015).
23. TD Lewin, PK Maini, EG Moros, H Enderling, HM Byrne, The evolution of tumour composition during fractionated radiotherapy: implications for outcome. *Bull. Math. Biol.* **80**, 1207–1235 (2018).
24. GN Betts, et al., Prospective technical validation and assessment of intra-tumour heterogeneity of a low density array hypoxia gene profile in head and neck squamous cell carcinoma. *Eur. J. Cancer* **49**, 156–165 (2013).
25. JA Bull, et al., Combining multiple spatial statistics enhances the description of immune cell localisation within tumours. *Sci. Reports* **10** (2020).
26. R Achanta, et al., SLIC superpixels compared to state-of-the-art superpixel methods. *IEEE Transactions on Pattern Analysis Mach. Intell.* **34**, 2274–2281 (2012).
27. C Cortes, V Vapnik, Support-vector networks. *Mach. Learn.* **20**, 273–297 (1995).
28. K Wijffels, et al., Vascular architecture and hypoxic profiles in human head and neck squamous cell carcinomas. *Br. J. Cancer* **83**, 674–683 (2000).
29. MW Dewhirst, et al., Perivascular oxygen tensions in a transplantable mammary tumor growing in a dorsal flap

window chamber. *Radiat. Res.* **130**, 171–182 (1992).

30. CC Wykoff, et al., Hypoxia-inducible expression of tumor-associated carbonic anhydrases. *Cancer Res.* **60**, 7075–7083 (2000).
31. JA Raleigh, SC Chou, GE Arteel, MR Horsman, Comparisons among pimonidazole binding, oxygen electrode measurements, and radiation response in C3H mouse tumors. *Radiat. Res.* **151**, 580–589 (1999).
32. M Höckel, K Schlenger, C Knoop, P Vaupel, Oxygenation of carcinomas of the uterine cervix: evaluation by computerized O2 tension measurements. *Cancer Res.* **51**, 6098–6102 (1991).
33. M Gameiro, et al., A topological measurement of protein compressibility. *Jpn. J. Ind. Appl. Math.* **32**, 1–17 (2015).
34. M Nicolau, AJ Levine, G Carlsson, Topology based data analysis identifies a subgroup of breast cancers with a unique mutational profile and excellent survival. *Proc. Natl. Acad. Sci. United States Am.* **108**, 7265–7270 (2011).
35. B Keller, M Lesnick, TL Willke, PHoS: Persistent Homology for Virtual Screening. *ChemRxiv* (2018).
36. F Belchi, et al., Lung topology characteristics in patients with chronic obstructive pulmonary disease. *Sci. Reports* **8**, 5341 (2018).
37. L Kanari, et al., A topological representation of branching neuronal morphologies. *Neuroinformatics* **16**, 3–13 (2018).
38. N Singh, HD Couture, JS Marron, C Perou, M Niethammer, Topological descriptors of histology images in *Machine Learning in Medical Imaging*, eds. G Wu, D Zhang, L Zhou. (Springer International Publishing, Cham), pp. 231–239 (2014).
39. P Lawson, AB Sholl, JQ Brown, BT Fasy, C Wenk, Persistent homology for the quantitative evaluation of architectural features in prostate cancer histology. *Sci. Reports* **9** (2019).
40. O Vipond, *Multiparameter Persistence Examples* (2020) https://olivervipond.github.io/Multiparameter_Persistence_Examples/#/.
41. H Edelsbrunner, JL Harer, *Computational Topology: An Introduction.* (American Mathematical Society, Providence, R.I), (2010).
42. S Oudot, *Persistence theory : from quiver representations to data analysis.* (American Mathematical Society, Providence, Rhode Island), (2015).
43. W Crawley-Boevey, Decomposition of pointwise finite-dimensional persistence modules. *J. Algebr. Its Appl.* **14**, 1550066 (2015).
44. G Carlsson, A Zomorodian, The Theory of Multidimensional Persistence. *Discret. & Comput. Geom.* **42**, 71–93 (2009).
45. M Lesnick, M Wright, Interactive Visualization of 2-D Persistence Modules. *arXiv e-prints*, arXiv:1512.00180 (2015).
46. E Miller, Data structures for real multiparameter persistence modules. *arXiv e-prints*, arXiv:1709.08155 (2017).
47. HB Bjerkevik, MB Botnan, M Kerber, Computing the Interleaving Distance is NP-Hard. *Foundations Comput. Math.* (2019).
48. O Vipond, Multiparameter Persistence Landscapes. *J. Mach. Learn. Res.* **21**, 1–38 (2020).
49. M Lesnick, The theory of the interleaving distance on multidimensional persistence modules. *Foundations Comput. Math.* **15**, 613–650 (2015).
50. H Adams, et al., Persistence images: A stable vector representation of persistent homology. *J. Mach. Learn. Res.* **18**, 1–35 (2017).
51. P Bubenik, Statistical topological data analysis using persistence landscapes. *J. Mach. Learn. Res.* **16**, 77–102 (2015).
52. F Chazal, BT Fasy, F Lecci, A Rinaldo, LA Wasserman, Stochastic convergence of persistence landscapes and silhouettes. *J. Compuational Geom.* **6**, 140–161 (2014).
53. A Adcock, E Carlsson, G Carlsson, The ring of algebraic functions on persistence bar codes. *Homol. Homotopy Appl.* **18**, 381–402 (2016).
54. S Kališnik, Tropical coordinates on the space of persistence barcodes. *Foundations Comput. Math.* **19**, 101–129 (2019).
55. R Corbet, U Fugacci, M Kerber, C Landi, B Wang, A kernel for multi-parameter persistent homology. *Comput. & Graph. X* **2**, 100005 (2019).
56. M Carrière, A Blumberg, Multiparameter persistence image for topological machine learning. *Adv. Neural Inf. Process. Syst.* **33** (2020).
57. BJ Stolz, T Emerson, S Nahkuri, MA Porter, HA Harrington, Topological Data Analysis of Task-Based fMRI Data from Experiments on Schizophrenia. *J. Phys. Complex.* **2**, 035006 (2021).

58. V Kovacev-Nikolic, P Bubenik, D Nikolic, G Heo, Using persistent homology and dynamical distances to analyze protein binding. *Stat. Appl. Genet. Mol. Biol.* **15** (2016).
59. P Dłotko, T Wanner, Topological microstructure analysis using persistence landscapes. *Phys. D: Nonlinear Phenom.* **334**, 60 – 81 (2016).
60. P Bubenik, The persistence landscape and some of its properties in *Topological Data Analysis*, Abel Symposia, vol 15. (Springer, Cham), pp. 97–117 (2020).
61. P Bubenik, P Dłotko, A persistence landscapes toolbox for topological statistics. *J. Symb. Comput.* **78**, 91 – 114 (2017).
62. L Betthauser, P Bubenik, PB Edwards, Graded persistence diagrams and persistence landscapes. *arXiv e-prints*, arXiv:1904.12807 (2019).
63. JA Bull, *Characterisation of macrophage infiltration into solid tumours via image analysis and computational modelling.* DPhil Thesis, University of Oxford (2018) https://ora.ox.ac.uk/objects/uuid:6319ffed-8db3-4e10-924b-cfa677923d34.
64. V de Silva, G Carlsson, Topological estimation using witness complexes in *SPBG'04 Symposium on Point - Based Graphics 2004*, eds. M Gross, H Pfister, M Alexa, S Rusinkiewicz. (The Eurographics Association), (2004).
65. AJ Blumberg, I Gal, MA Mandell, M Pancia, Robust statistics, hypothesis testing, and confidence intervals for persistent homology on metric measure spaces. *Foundations Comput. Math.* **14**, 745–789 (2014).
66. M Buchet, F Chazal, SY Oudot, DR Sheehy, Efficient and Robust Persistent Homology for Measures in *ACM-SIAM Symposium on Discrete Algorithms.* (San Diego, United States), (2015).
